# Supplementary material for: Longer scans boost prediction and cut costs in brain-wide association studies
Source: Nature. 2025 Jul 16;644(8077):731–40. doi: 10.1038/s41586-025-09250-1 (PMC12367542; doi:10.1038/s41586-025-09250-1)
Supplement: Supplementary file 1 — This file includes Supplementary Figures, Tables, References and a list of Consortium Contributor Alzheimer’s Disease Neuroimaging Initiative [file 41586_2025_9250_MOESM1_ESM.docx]

**Longer scans boost prediction and cut costs in brain-wide association studies**

Leon Qi Rong Ooi^1-5♦^, Csaba Orban^2,3,5♦^, Shaoshi Zhang^1-5♦^, Thomas E. Nichols^6,7^, Trevor Wei Kiat Tan^1-5^, Ru Kong^2-5^_,_ Scott Marek^8,9^, Nico U.F. Dosenbach^8-13^_,_ Timothy O. Laumann^9,14^_,_ Evan M. Gordon^8,9^, Kwong Hsia Yap^15,16^, Fang Ji ^2,3^, Joanna Su Xian Chong^2,3^, Christopher Chen^15,16^, Lijun An^17^, Nicolai Franzmeier^18-20^, Sebastian N. Roemer-Cassiano^18,21^, Qingyu Hu^22^, Jianxun Ren^22^, Hesheng Liu^22,23^, Sidhant Chopra^24,25^, Carrisa V. Cocuzza^26,27^, Justin T. Baker^28,29^, Juan Helen Zhou^1-4^, Danilo Bzdok^30-32^, Simon B. Eickhoff^33,34^, Avram J. Holmes^27^, B. T. Thomas Yeo^1-5,35*^, Alzheimer’s Disease Neuroimaging Initiative

^1^Integrative Sciences and Engineering Programme (ISEP), National University of Singapore

^2^Centre for Sleep and Cognition & Centre for Translational MR Research, Yong Loo Lin School of Medicine, National University of Singapore, Singapore

^3^Department of Medicine, Healthy Longevity Translational Research Programme, Human Potential Translational Research Programme & Institute for Digital Medicine (WisDM), Yong Loo Lin School of Medicine, National University of Singapore, Singapore

^4^Department of Electrical and Computer Engineering, National University of Singapore, Singapore

^5^N.1 Institute for Health, National University of Singapore, Singapore

^6^Big Data Institute, Li Ka Shing Centre for Health Information and Discovery, Nuffield Department of Population Health, University of Oxford, Oxford, UK

^7^Centre for Integrative Neuroimaging (OxCIN), FMRIB, Nuffield Department of Clinical Neurosciences, University of Oxford, Oxford, UK

^8^Mallinckrodt Institute of Radiology, Washington University School of Medicine, St. Louis, USA

^9^Allied Labs for Imaging Guided Neurotherapies (ALIGN), Washington University School of Medicine, St. Louis, USA

^10^Department of Neurology, Washington University School of Medicine, St. Louis, USA

^11^Department of Paediatrics, Washington University School of Medicine, St. Louis, USA

^12^Department of Biomedical Engineering, Washington University, St. Louis, USA

^13^Department of Psychological and Brain Sciences, Washington University, St. Louis, USA

^14^Department of Psychiatry, Washington University, School of Medicine, St. Louis, USA

^15^Memory, Ageing and Cognition Centre, National University Health System, Singapore

^16^Department of Pharmacology, Yong Loo Lin School of Medicine, National University of Singapore, Singapore

^17^Department of Clinical Sciences, Malmö, SciLifeLab, Lund University, Lund, Sweden

^18^Institute for Stroke and Dementia Research, LMU Munich, Munich, Germany

^19^Munich Cluster for Systems Neurology (SyNergy), Munich, Germany

^20^Department of Psychiatry and Neurochemistry, Institute of Neuroscience and Physiology, University of Gothenburg, The Sahlgrenska Academy, Gothenburg, Sweden

^21^Department of Neurology, LMU Hospital, LMU Munich, Munich, Germany

^22^Changping Laboratory, Beijing, China

^23^Biomedical Pioneering Innovation Center (BIOPIC), Peking University, Beijing, China

^24^Orygen, Parkville, Australia

^25^Center for Youth Mental Health, University of Melbourne, Melbourne, Australia

^26^Department of Psychology, Yale University, New Haven, CT, USA

^27^Department of Psychiatry, Brain Health Institute, Rutgers University, Piscataway, NJ, USA

^28^Department of Psychiatry, Harvard Medical School, Boston, USA

^29^Institute for Technology in Psychiatry, McLean Hospital, Boston, USA

^30^The Neuro, McConnell Brain Imaging Centre, Department of Biomedical Engineering, Canada

^31^Faculty of Medicine, School of Computer Science, McGill University, Montreal, QC, Canada

^32^Mila - Quebec Artificial Intelligence Institute, Montreal, QC, Canada

^33^Institute of Neuroscience and Medicine, Brain & Behaviour (INM-7), Research Center Jülich, Jülich, Germany

^34^Institute for Systems Neuroscience, Medical Faculty, Heinrich-Heine University Düsseldorf, Düsseldorf, Germany

^35^Martinos Center for Biomedical Imaging, Massachusetts General Hospital, Charlestown, MA, USA

♦ Indicates that these authors contributed equally

* Address correspondence to:

B.T. Thomas Yeo

CSC, TMR, ECE, N.1, WISDM

National University of Singapore

Email: [thomas.yeo@nus.edu.sg](mailto:thomas.yeo@nus.edu.sg)

**Table of Contents**

[Supplementary methods 5](#_Toc199326771)

[1.1 Preliminaries: 1-edge results 5](#_Toc199326772)

[1.2 FC-phenotype prediction accuracy 7](#_Toc199326773)

[1.2.1 Empirical curve fitting for prediction accuracy 9](#_Toc199326774)

[1.2.2 Intuition under restrictive independence assumptions 10](#_Toc199326775)

[1.3 Edgewise reliability of FC-phenotype association 11](#_Toc199326776)

[1.3.1 Empirical curve fitting for edgewise reliability 12](#_Toc199326777)

[References 14](#_Toc199326778)

[Supplemental material 15](#_Toc199326779)

[Supplementary Tables 15](#_Toc199326780)

[Supplementary Table 1. Statistical analyses. 15](#_Toc199326781)

[Supplementary Table 2. Summary of prediction accuracy analyses in the ABCD and HCP datasets. 27](#_Toc199326782)

[Supplementary Table 3. Summary of prediction accuracy analyses for all datasets. 29](#_Toc199326783)

[Supplementary Tables 4.1-4.6. Phenotypic Measures in each dataset. 30](#_Toc199326784)

[Supplementary Tables 5.1-5.3. Distribution details for each dataset. 39](#_Toc199326785)

[Supplementary Tables 6.1-6.3. Site clusters used for each dataset. 41](#_Toc199326786)

[Supplementary Figures 45](#_Toc199326787)

[Supplementary Fig. 1 | Contour plot of HCP prediction accuracy. 45](#_Toc199326788)

[Supplementary Fig. 2 | Contour plot of ABCD & HCP prediction accuracy in terms of COD. 46](#_Toc199326789)

[Supplementary Fig. 3 | Contour plot of ABCD & HCP prediction after censoring. 47](#_Toc199326790)

[Supplementary Fig. 4 | Contour plot of ABCD & HCP prediction accuracy using data without censoring. 48](#_Toc199326791)

[Supplementary Fig. 5 | Contour plot of ABCD & HCP prediction accuracy (Pearson’s Correlation) using LRR. 49](#_Toc199326792)

[Supplementary Fig. 6 | Contour plot of ABCD & HCP prediction accuracy (COD) using LRR. 50](#_Toc199326793)

[Supplementary Fig. 7.1-7.3 | Scatter plots for 17 phenotypic measures in the ABCD dataset. 51](#_Toc199326794)

[Supplementary Fig. 8.1-8.7 | Scatter plots for 19 phenotypic measures in the HCP dataset. 56](#_Toc199326795)

[Supplementary Fig. 9 | Consistent logarithmic relationship across algorithms and metrics 67](#_Toc199326796)

[Supplementary Fig. 10.1-10.3 | Theoretical model fit for 17 phenotypic measures in the ABCD dataset. 68](#_Toc199326797)

[Supplementary Fig. 11.1-11.4 | Theoretical model fit for 19 phenotypic measures in the HCP dataset. 73](#_Toc199326798)

[Supplementary Fig. 12 | Theoretical model outperforms logarithmic model for longer scan times. 77](#_Toc199326799)

[Supplementary Fig. 13.1-13.3 | Theoretical model fit for 14 phenotypic measures in the SINGER dataset. 78](#_Toc199326800)

[Supplementary Fig. 14.1-14.2 | Theoretical model fit for 7 phenotypic measures in the TCP dataset. 82](#_Toc199326801)

[Supplementary Fig. 15.1-15.2 | Theoretical model fit for 7 phenotypic measures in the MDD dataset. 84](#_Toc199326802)

[Supplementary Fig. 16 | Theoretical model fit for 6 phenotypic measures in the ADNI dataset. 87](#_Toc199326803)

[Supplementary Fig. 17.1-17.3 | Theoretical model fit for 16 phenotypic measures using the ABCD MID task data. 88](#_Toc199326804)

[Supplementary Fig. 18.1-18.4 | Theoretical model fit for 19 phenotypic measures using the ABCD N-back task data. 92](#_Toc199326805)

[Supplementary Fig. 19.1-19.3 | Theoretical model fit for 18 phenotypic measures using the ABCD SST task data. 96](#_Toc199326806)

[Supplementary Fig. 20 | Reliability split-half procedure. 101](#_Toc199326807)

[Supplementary Fig. 21 | Correlation between univariate BWAS reliability of ABCD and HCP cognition factor scores. 102](#_Toc199326808)

[Supplementary Fig. 22 | Logarithmic relationship to univariate BWAS. 104](#_Toc199326809)

[Supplementary Fig. 23 | Theoretical model fit to univariate brain-wide reliability. 105](#_Toc199326810)

[Supplementary Fig. 24 | Interchangeability of sample size and scan time for reliability. 106](#_Toc199326811)

[Supplementary Fig. 25.1-25.3 | Reliability theoretical model fit for 17 phenotypic measures in the ABCD dataset. 107](#_Toc199326812)

[Supplementary Fig. 26.1-26.4 | Reliability theoretical model fit for 19 phenotypic measures in the HCP dataset. 113](#_Toc199326813)

[Supplementary Fig. 27 | Contour plot of ABCD & HCP multivariate BWAS reliability. 119](#_Toc199326814)

[Supplementary Fig. 28 | Correlation between multivariate BWAS reliability of ABCD and HCP cognition factor scores. 120](#_Toc199326815)

[Supplementary Fig. 29 | Logarithmic relationship to multivariate BWAS. 122](#_Toc199326816)

[List of Consortium Contributors Alzheimer’s Disease Neuroimaging Initiative 123](#_Toc199326817)

[Author affiliations for the Alzheimer’s Disease Neuroimaging Initiative 126](#_Toc199326818)

# Supplementary methods

This section provides approximations that justify the form of key outcomes as a function of sample size $N$ and scan time $T$. We consider two types of outcomes, the correlation between a linear prediction of non-brain-imaging phenotype (henceforth referred to as phenotype) using functional connectivity (FC), and the reliability of edge-wise FC-phenotypic correlation after participant-wise data splitting.

It is important to note that the following derivations are general and not limited to functional connectivity, and are in fact applicable to the relationship between any phenotype with measurements from any sensor (not necessarily MRI).

## Preliminaries: 1-edge results

Define the target phenotype variable for participant $i$ as

$Y_{i}=\psi_{i}+\xi_{i}$ (S1)

for $i=1,\ldots,N$, where $\psi_{i}$ is the noise-free, latent phenotype that $Y$ attempts to measure and $\xi_{i}$ is the random error. For a typical phenotypic trait, $\psi_{i}$ could be thought of as a measurement obtainable if we had multiple days and endless tests to acquire for each participant; $\xi_{i}$ is the divergence between that ideal value and $Y_{i}$. Let the variance of the true phenotype be $\sigma_{\psi}^{2}$, and for the measurement error $\sigma_{\xi}^{2}$. The intraclass correlation reliability of the phenotype is given by $R\left( Y \right)=\frac{\sigma_{\psi}^{2}}{(\sigma_{\psi}^{2}+\sigma_{\xi}^{2})}$.

Let the FC measure for participant $i$, edge $j$ based on $T$ scans be $X_{Tij}$, formed into a length-$J$ row vector $X_{Ti}$. The observable FC is also a noisy measure of some ideal FC; for scan length $T$, participant $i$ and edge $j$, this can be written as

$X_{Tij}=\theta_{ij}+\epsilon_{\mathrm{Tij}}$ (S2)

where $\theta_{ij}$ is the noise-free FC. The values $\theta_{ij}$ can be considered as the FC value we would obtain if we leave the participant in the scanner for so long that measurement error becomes negligible. The measurement error $\epsilon_{Tij}$ is specific to the acquisition time $T$, as precision will increase with longer scan time. Let the variance of true FC be $\sigma_{\theta_{j}}^{2}$ (i.e. the participant-to-participant variability in true FC at edge $j$). The measurement error for FC is more involved.

If fMRI time series had no temporal autocorrelation, the sampling variance of Pearson’s correlation would be well approximated by

$\frac{1}{T}\left( 1-\theta_{ij}^{2} \right)^{2}$. (S3)

However, as covered in detail in Afyouni et al., 2019, the distinct temporal autocorrelation in each node and the cross-correlation (at all lags) influence the sampling variance of Pearson’s correlation in a complex fashion. For simplicity, we just assume that there is some normalized variance $\tau_{j}^{2}$ such that $V\left( \epsilon_{Tij} \right)=\frac{\tau_{j}^{2}}{T}$ (strictly, we should keep track of participant-specific variance $\frac{\tau_{ij}^{2}}{T}$ since it depends on each participant’s correlation $\theta_{ij}$ and autocorrelation; however, this is the first of many simplifications we make to obtain tractable results). Thus the intraclass correlation reliability of FC is then $R\left( X_{Tj} \right)=\frac{\sigma_{\theta_{j}}^{2}}{\sigma_{\theta_{j}}^{2}+\frac{\tau_{j}^{2}}{T}}$. The ideal FC-phenotype correlation for edge $j$ is

$\rho_{j}=\text{corr}(\theta_{ij},\psi_{i})$. (S4)

However, we cannot directly observe these noise-free measurements, as each is corrupted by measurement noise. Due to a classic result by Spearman, 1904, we know that when corrupted measures are used to compute $\hat{\rho}_{j}=\hat{\text{corr}}(X_{Tij},Y_{i})$ the result is biased, with

$E\left( \hat{\rho}_{j} \right) = \rho_{j}\sqrt{R(X_{Tj})}\sqrt{R(Y)}$ (S5)

$= \rho_{j}\sqrt{\frac{1}{1+\frac{(\tau_{j}^{2}/\sigma_{\theta_{j}}^{2})}{T}}}\sqrt{\frac{1}{1+\frac{\sigma_{\xi}^{2}}{\sigma_{\psi}^{2}}}}.$ (S6)

This shows the dependence of edge-wise FC-phenotype association on scan length *T*. If no information is available on the reliability of the phenotype, we can simply act as if there is no measurement error $(\sigma_{\xi}^{2}=0)$ and then there is no dependence on phenotype variability $\sigma_{\psi}^{2}$.

Below we will also need the variance of $\hat{\rho}_{j}$ , which is simply the variance for Pearson’s correlation for variables where the true correlation is $\rho_{j}\sqrt{R\left( X_{Tj} \right)R(Y)}$,

$V\left( \hat{\rho}_{j} \right)=\frac{1}{N}\left( 1-\rho_{j}^{2}R\left( X_{Tj} \right)R\left( Y \right) \right)^{2}$ (S7)

which is the same as Equation (S3) except applied over participants.

Note that it will be useful to approximate this with a 2^nd^ order Taylor series approximation for $f\left( t \right)=\left( 1-t^{2} \right)^{2}$ about $t=0$, $1-2t^{2}$, here

$V\left( \hat{\rho}_{j} \right)\approx\frac{1}{N}\left( 1-2\rho_{j}^{2}R\left( X_{Tj} \right)R\left( Y \right) \right)$ (S8)

which we find to be fairly accurate up to $t=\rho_{j}\sqrt{R\left( X_{Tj} \right)R(Y)}=0.5$; recall that, $\rho_{j}\sqrt{R\left( X_{Tj} \right)R(Y)}$ is the true correlation between FC edge $j$ and a phenotype for a fMRI acquisition of length $T$. We will use this approximation for the reliability analysis (Supplementary Methods S1.3). Obviously, we do not know the true correlation between FC edge $j$ and a phenotype. However, we can compute the measured correlation between FC and a phenotype to check the quality of the approximation. The phenotype with the strongest correlation with FC is the cognitive factor score. In the case of the HCP dataset, across all edges, the largest absolute correlation between FC and the cognitive factor was 0.27, while in the case of the ABCD dataset, across all edges, the largest absolute correlation between FC and the cognitive factor was 0.22. As the strongest correlation is much smaller than 0.5, we believe that this is a satisfactory approximation.

## FC-phenotype prediction accuracy

While the body of the paper presents results for kernel ridge regression, we found that linear ridge regression gave very similar results. The analysis here is only for linear regression and is provided to motivate the role that $N$ and $T$ might play in the FC-phenotype prediction accuracy measured with correlation.

First, for subject $i$, we can write the linear predictor of the noise-free phenotype $\psi_{i}$ using noise-free FC $\theta_{i}$ as $\theta_{i}\beta_{i}$, where $\beta_{i}$ is the ideal length-$J$ vector of regression weights. Define the true, ideal correlation as

$\rho=\text{corr}(\theta_{i}\beta_{i},\psi_{i})$. (S9)

In practice, we can at best compute $\hat{\rho}=\hat{\text{corr}}(x_{Ti}\hat{\beta},Y_{i})$, for which stated results in the previous section give us

$E\left( \hat{\rho} \right)=\rho\sqrt{R\left( X_{T}\hat{\beta} \right)}\sqrt{R\left( Y \right)}$ (S10)

With slight abuse of notation, here we use $\rho$ for the regression-based noise free FC-phenotype association over edges, and $\hat{\rho}$ as the noise-corrupted association; these are distinct from the previous $\rho_{j}$ and $\hat{\rho}_{j}$, the edgewise noise-free and noise-corrupted FC-phenotype associations respectively.

Calculating the sample variance of $X_{Ti}\hat{\beta}$ to compute $R(X_{T}\hat{\beta})$ is challenging: standard regression results don’t apply since they neglect using the noisy FC $X_{Ti}$ instead of the ideal $\theta_{i}$, in what is known as an “Errors-in-Variables” problem.

To find the sample variance of $X_{Ti}\hat{\beta}$ when OLS is used to estimate a errors-in-variables model we rely on the results from Gleser et al., 1987 (GCG). Following GCG, the regression is partitioned into known (error-free) variables $F_{1}(N\times P)$ and unobserved variables $F_{2}(N\times J)$, for which we observe a noisy version $X$:

$Y=F_{1}\beta_{1}+F_{2}\beta_{2}+e$ (S11)

$X=F_{2}+U.$ (S12)

All useful results require normality of the errors $e$ and the corrupting noise $U$, with the joint distribution of these stochastic components having $\left( 1+J \right)\times(1+J)$ covariance

$\text{Cov}\left( \left[ e_{i},u_{i} \right] \right)=\Sigma= \left[ \begin{matrix} \sigma_{11}^{2} & \sigma_{12} \\ \sigma_{12}^{\top} & \Sigma_{22} \end{matrix} \right]$ (S13)

where $u_{i}$ is the $J$-vector of covariate errors, $\sigma_{11}^{2}$ is the variance of the residual error (in the ideal model with $F_{1}$ and $F_{2}$), $\sigma_{12}$ the $1\times J$ covariance between the residual error and the $F_{2}$ corrupting noise, and $\Sigma_{22}$ the $J\times J$ covariance of the corrupting noise.

GCG gives the properties of the OLS regression with $\beta^{\top}=(\beta_{1}^{\top},\beta_{2}^{\top})$ when the design matrix $[F_{1} X]$ is used. The results depend on the limiting mean-squares of the covariates

$\lim_{N\to\infty} \frac{1}{N}\left[ \begin{matrix} F_{1}^{\top}F_{1} & F_{1}^{\top}F_{2} \\ F_{2}^{\top}F_{1} & F_{2}^{\top}F_{2} \end{matrix} \right]= \left[ \begin{matrix} \Delta_{11} & \Delta_{12} \\ \Delta_{12}^{\top} & \Delta_{22} \end{matrix} \right]$ (S14)

With errors-in-variables OLS is biased, with $\hat{\beta}$ having asymptomatic expectation

$\beta+\left[ \begin{matrix} \Delta_{11} & \Delta_{12} \\ \Delta_{12}^{\top} & \Delta_{22} \end{matrix} \right]^{-1}\left( \begin{matrix} 0 \\ \gamma\end{matrix} \right)=\beta+\left[ \begin{matrix} -\Delta_{11}^{-1}\Delta_{12}\left( \Delta_{22}-\Delta_{12}^{\top}\Delta_{11}^{-1}\Delta_{12} \right)^{-1}\gamma\\ \left( \Delta_{22}-\Delta_{12}^{\top}\Delta_{11}^{-1}\Delta_{12} \right)^{-1}\gamma\end{matrix} \right]$ (S15)

where

$\gamma=\sigma_{12}^{\top}-\Sigma_{22}\beta_{2}.$ (S16)

We use GCG’s Theorem 2 that unfortunately is narrowly stated for contrasts $C\hat{\beta}$ where OLS is unbiased, i.e. where $C$ has a certain form, $C=[I_{P},\Delta_{11}^{-1}\Delta_{12}]$. Theorem 2 states that the asymptotic variance of $C\hat{\beta}$ is

$\text{Cov}\left( C\hat{\beta} \right)=\frac{1}{N}\eta^{\top}\Sigma\eta\Delta_{11}^{-1}$ (S17)

where

$\eta= \left[ \begin{matrix} 1 \\ -(\beta_{2}+Q\gamma) \end{matrix} \right]$ (S18)

$Q = \left( \Delta_{22.1}+\Sigma_{22} \right)^{-1}$ (S19)

$\Delta_{22.1} = \Delta_{22}-\Delta_{12}^{\top}\Delta_{11}^{-1}\Delta_{12}$ (S20)

$\gamma= \sigma_{12}^{\top}-\Sigma_{22}\beta_{2}.$ (S21)

In our setting, we have a single ($P=1)$ error-free predictor, with $F_{1}$ being a column of $1$’s for the intercept, and thus $\Delta_{11}=1$ (a scalar) and $\Delta_{12}=\bar{\theta}$ is the $1\times J$ vector of means of the noiseless FC edges over $N$ participants; the $J$ noise-corrupted FC measurements make up $X$, and the elements of $U$ are exactly the measurement errors $\epsilon_{Tij}$. A common assumption is that the model error $e$ is uncorrelated with the measurement noise, and hence $\sigma_{12}=0$. Further, we can capture the $T$-dependence of the measurement error by assuming,

$\Sigma_{22}=\frac{1}{T}\Sigma_{22}^{*}$ (S22)

that is, that $\Sigma_{22}^{*}$ is a normalized measurement error covariance that scales by $\frac{1}{T}$ to give the actual measurement error. As an aside, under these settings, OLS $\hat{\beta}$ has mean

$\beta+\frac{1}{T}\left[ \begin{matrix} -\bar{\theta}\Delta_{22.1}^{-1}\Sigma_{22}^{*}\beta_{2} \\ \Delta_{22.1}^{-1}\Sigma_{22}^{*}\beta_{2} \end{matrix} \right].$ (S23)

Note in our setting $\Delta_{22.1}$ is the limiting covariance of the (unobserved, noise-free) FC design matrix $F_{2}$. The reason is that in our case $\Delta_{11}^{-1}=1$, so $\Delta_{22.1}=\Delta_{22}-\Delta_{12}^{\top}\Delta_{11}^{-1}\Delta_{12}=E\left( F_{2}^{T}F_{2} \right)-E^{T}\left( F \right)E(F)$, which is the standard variance formula.

In our setting $C=\left[ 1,\bar{\theta} \right]$, which is the intercept plus (true) mean FC. Instead, we want, for each participant $i$, $\hat{Y}_{i}=\left[ 1,X_{i} \right]\hat{\beta}$, the linear combination of $\hat{\beta}$ that is the intercept plus the J elements of $\hat{\beta}$ weighted according to that participant’s FC measurements. Hence, strictly, the Theorem 2 result is relevant to the prediction for the average participant (using the noise-free FC), however we use this to gauge the properties of the sampling variance of the prediction.

To simplify the main result, let $\beta_{2}^{*}=\beta_{2}-\left( \Delta_{22.1}+\frac{1}{T}\Sigma_{22}^{*} \right)^{-1}\frac{1}{T}\Sigma_{22}^{*}\beta_{2}$, which converges to $\beta_{2}$ as $T$ grows. Then in our application, the asymptotic variance is

$\text{Var}\left( C\hat{\beta} \right)=\frac{1}{N}\left[ 1,-{\beta_{2}^{*}}^{\top} \right]\left[ \begin{matrix} \sigma_{11}^{2} & 0^{\top} \\ 0 & \frac{\Sigma_{22}^{*}}{T} \end{matrix} \right]\left[ 1,-{\beta_{2}^{*}}^{\top} \right]^{\top}$ (S24)

$=\frac{1}{N}\sigma_{11}^{2}+\frac{1}{TN}{\beta_{2}^{*}}^{\top}\Sigma_{22}^{*}\beta_{2}^{*}$ (S25)

We can see that this variance has 2 terms: the first is the residual variance in $Y$ not explained by (noiseless) FC; the second contains the contribution of FC measurement error, which we have expressed relative to the normalized $\Sigma_{22}^{*}$, showing the dependence on $\frac{1}{TN}$.

Finally, to compute reliability, we have

$R\left( X_{T}\hat{\beta} \right)=\frac{S_{\theta\beta}^{2}}{S_{\theta\beta}^{2}+\frac{1}{N}\sigma_{11}^{2}+\frac{1}{TN}{\beta_{2}^{*}}^{\top}\Sigma_{22}^{*}\beta_{22}^{*}}$ (S26)

where $S_{\theta\beta}^{2}$ is the “true” variation of interest, the inter-participant variation in the predictions using noise-free FC $\theta$ and ideal regression coefficients $\beta$. Then the expected regression FC-phenotype correlation is

$E\left( \hat{\rho} \right)\approx\rho\sqrt{\frac{S_{\theta\beta}^{2}}{S_{\theta\beta}^{2}+\frac{1}{N}\sigma_{11}^{2}+\frac{1}{TN}{\beta_{2}^{*}}^{\top}\Sigma_{22}^{*}\beta_{22}^{*}}}\sqrt{\frac{\sigma_{\psi}^{2}}{\sigma_{\psi}^{2}+\sigma_{\xi}^{2}}}$ (S27)

## Empirical curve fitting for prediction accuracy

When we estimate a function of observed correlations as a function of $N$ and $T$

$K_{0}\sqrt{\frac{1}{1+\frac{1}{N}K_{1}+\frac{1}{TN}K_{2}}}$ (S28)

we can interpret $K_{0}=\rho R\left( Y \right)$ as the ideal association attenuated by phenotype reliability. Noting that $\rho^{2}=\frac{S_{\theta\beta}^{2}}{\sigma_{\psi}^{2}}$, the proportion of noise-free phenotype explained by the ideal prediction, and $\sigma_{11}^{2}=\left( 1-\rho^{2} \right)\sigma_{\psi}^{2}$ is the noise-free phenotype variance not explained, then

$K_{1}=\frac{\sigma_{11}^{2}}{S_{\theta\beta}^{2}}=\frac{\left( 1-\rho^{2} \right)\sigma_{\psi}^{2}}{\rho^{2}\sigma_{\psi}^{2}}=\frac{{1-\rho}^{2}}{\rho^{2}}$ (S29)

as the inverse Cohen’s $f^{2}$ of the (noise-free, ideal) prediction ($f^{2}$ is an effect size often similar to $R^{2}$), and

$K_{2}=\frac{{\beta_{2}^{*}}^{\top}\Sigma_{22}^{*}\beta_{2}^{*}}{S_{\theta\beta}^{2}}=\frac{{\beta_{2}^{*}}^{\top}\Sigma_{22}^{*}\beta_{2}^{*}}{\rho^{2}\sigma_{\psi}^{2}}$ (S30)

as the measurement error relative to the variance explained with noise-free ideal prediction.

These interpretations, however, should be tempered by the many assumptions leading up to this result. The principle critical assumptions are $\sigma_{12}=0$, in that ideal prediction errors are uncorrelated with measurement errors, and that we’re using a variance result for the prediction of a participant with the (true) average FC value for each edge, which may not be representative overall.

## Intuition under restrictive independence assumptions

Returning to $R(X_{T}\hat{\beta})$, note that if we assume independent measurement errors along the $J$ edges, then $\Sigma_{22}^{*}=\text{diag}\left( \left\{ \tau_{j}^{2} \right\} \right)$ and ${\beta_{2}^{*}}^{\top}\Sigma_{22}^{*}\beta_{22}^{*}=\Sigma_{j}{\beta_{2j}^{*}}^{2}\tau_{j}^{2}$. Further, if we can assume that noise-free FC is (1) normalized to unit variance and (2) independent, then $\Delta_{22.1}=I$ and the measurement error contribution ${\beta_{2}^{*}}^{\top}\Sigma_{22}^{*}\beta_{2}^{*}$ further simplifies to $\Sigma_{j}\beta_{j}^{2}\tau_{j}^{2}\left( 1+\frac{\tau_{j}^{2}}{T} \right)^{-2}$. However, we realize that we can only ever normalize $X$ by empirical variance which is an over-estimate of noise-free FC, and that FC edges are highly structured and could never be anywhere near independent unless some very careful thinning was done. However, using $\rho^{2}$ and these restrictive assumptions, we obtain an alternate form of $R(X_{T}\hat{\beta})$ of

$\frac{\rho^{2}}{\rho^{2}+\frac{1}{N}\left( 1-\rho^{2} \right)+\frac{J}{TN}\left\langle\tau_{j}^{2}\left( \frac{1}{1+\frac{\tau_{j}^{2}}{T}} \right)^{2}\left( \frac{\beta_{j}}{\sigma_{\psi}} \right)^{2} \right\rangle}$ (S31)

which clearly shows the contributions of noise-free FC-phenotype association $\left( \rho^{2} \right)$, a $\left( \frac{1}{N} \right)$-weighted contribution of unexplained variation $\left( 1-\rho^{2} \right)$, and a $\left( \frac{1}{TN} \right)$-weighted contribution of measurement error and normalized (noise-free) regression coefficients, where the $\left\langle\cdot\right\rangle$ notation indicates average over edges. As any of $\rho^{2}$, $N$ or $T$ increases, reliability $R(X_{T\hat{\beta}})$ grows to an asymptote of 1; increases in measurement noise decrease reliability through a complex weighted fashion depending on the regression coefficients.

## Edgewise reliability of FC-phenotype association

We measure edgewise reliability of FC-phenotype association by splitting participants into two groups, computing FC-phenotype association at each of $J$ edges, and then correlating the associations over edges. Consider participants split into groups $A$ and $B$, $\frac{N}{2}$ in each, computing $\hat{\rho}_{Aj}$ and $\hat{\rho}_{Bj}$ for each edge $j$, with a model for these noisy correlations at edge $j$ of

$\hat{\rho}_{Aj}=\rho_{j}+\epsilon_{Aj}$ (S32)

$\hat{\rho}_{Bj}=\rho_{j}+\epsilon_{Bj}$ (S33)

where $\epsilon_{Aj}$ and $\epsilon_{Bj}$ are the random measurement error from the true association value $\rho_{j}$; note that since participants are split, these two errors are independent and from (S3) above, we know the variance is

$V\left( \hat{\rho}_{Aj} \right)=V\left( \hat{\rho}_{Bj} \right)\approx\frac{1}{N/2}\left( 1-\rho_{j}^{2}R\left( X_{Tj} \right)R\left( Y \right) \right)^{2}\approx\frac{1}{N/2}(1-2\rho_{j}^{2}R\left( X_{Tj} \right)R\left( Y \right))$ (S34)

using the (S8) to obtain a simplified form. If we use the true means instead of sample means, the correlation coefficient computed between $\hat{\rho}_{Aj}$ and $\hat{\rho}_{Bj}$ over edges is

$\frac{\sum_{j} \left( \hat{\rho}_{Aj}-\bar{\rho} \right)\left( \hat{\rho}_{Bj}-\bar{\rho} \right)}{\sqrt{\sum_{j} \left( \hat{\rho}_{Aj}-\bar{\rho} \right)^{2}}\sqrt{\sum_{j} \left( \hat{\rho}_{Bj}-\bar{\rho} \right)^{2}}}$ (S35)

where $\bar{\rho}=\frac{1}{J}\sum_{j} \rho_{j}$, the true FC-phenotype association averaged over edges. We proceed by approximating the expectation of this ratio as a ratio of expectations.

The expected value of the numerator is

$E\left( \sum_{j} \left( \hat{\rho}_{Aj}-\bar{\rho} \right)\left( \hat{\rho}_{Bj}-\bar{\rho} \right) \right)=\sum_{j} E((\rho_{j}-\bar{\rho}+\epsilon_{Aj})(\rho_{j}-\bar{\rho}+\epsilon_{Aj}))$ (S36)

$= \sum_{j} \left( E\left( \rho_{j}-\bar{\rho} \right)^{2}+E\left( \left( \rho_{j}-\bar{\rho} \right)\epsilon_{Bj} \right)+E\left( \left( \rho_{j}-\bar{\rho} \right)\epsilon_{Aj} \right)+ E\left( \epsilon_{Aj}\epsilon_{Bj} \right) \right)$ (S37)

$=JS_{\rho}^{2}$ (S38)

where $S_{\rho}^{2}$ is the inter-edge variance of the true FC-phenotype association, and the other terms are zero because the errors are uncorrelated with FC and between participant groups $A$ and $B$.

For the denominator term for $A$ (identical to that for $B$),

$E\left( \sum_{j} \left( \hat{\rho}_{Aj}-\bar{\rho} \right)^{2} \right)=\sum_{j} E\left( \left( \rho_{j}-\bar{\rho}+\epsilon_{Aj} \right)^{2} \right)$ (S39)

$=\sum_{j} \left( E\left( \left( \rho_{j}-\bar{\rho} \right)^{2} \right)+2E\left( \left( \rho_{j}-\bar{\rho} \right)\epsilon_{Aj} \right)+E\left( \epsilon_{Aj}^{2} \right) \right)$ (S40) $=JS_{\rho}^{2}+\frac{1}{N/2}\sum_{j} \left( 1-\rho_{j}^{2}R\left( X_{Tij} \right)R\left( Y \right) \right)^{2}$ (S41)

$\approx JS_{\rho}^{2}+\frac{J}{N/2}\left( 1-2\langle\rho_{j}^{2}R\left( X_{Tij} \right)\rangle R\left( Y \right) \right)$ (S42)

$=JS_{\rho}^{2}+\frac{J}{N/2}\left( 1-2\left\langle\rho_{j}^{2}\frac{1}{1+\frac{{\tau_{j}^{2}}/{\sigma_{\theta j}^{2}}}{T}} \right\rangle R\left( Y \right) \right)$ (S43)

again using (S8) to facilitate writing the sum of measurement error variance as an average over edges. If we then approximate an expectation of a product with a product of expectations, and expectation of a square root with square root of the expectation, we can use these to obtain the final result, an approximation to the expected value of the split-participants inter-edge correlation of FC-phenotype correlation:

$\frac{S_{\rho}^{2}}{S_{\rho}^{2}+\frac{1}{N/2}\left( 1-2\left\langle\rho_{j}^{2}\frac{1}{1+\frac{{\tau_{j}^{2}}/{\sigma_{\theta j}^{2}}}{T}} \right\rangle R(Y) \right)}.$ (S44)

This can be interpreted as follows: This reliability measure has 3 fundamental inputs: (1) Inter-edge variability of true association, $S_{\rho}^{2}$, (2) reliability of FC-phenotype correlation depending on $\rho_{j}^{2}$, $\tau_{j}^{2}$, and $\sigma_{\theta j}^{2}$, and (3) reliability of phenotype $R(Y)$. These last 2 are jointly scaled by $\frac{1}{N/2}$, but the FC-phenotype correlation reliability also has a $\frac{1}{T}$ dependence embedded in an inter-edge average such that, all else being equal, increased $T$ increases reliability. While the appearance of a negative term may be unexpected, it directly follows from the variance of Pearson’s correlation (S3) and as approximated by (S8): variance of Pearson’s correlation is maximal when true correlation is 0, and decreases with the absolute value of correlation; the reliability terms attenuate the correlation, and thus increase the sample variance. Note also that further simplification would be possible as $\left\langle\rho_{j}^{2} \right\rangle=S_{\rho}^{2}+\bar{\rho}^{2}$, i.e. the FC variability is also captured by the averaged squared true correlation, but this is complicated by the edgewise weighting involving ${\tau_{j}^{2}}/{\sigma_{\theta j}^{2}}$.

## Empirical curve fitting for edgewise reliability

The reliability result (S44) doesn’t make a simple prediction for the interplay of the $T$ and $N$ terms, as the influence of $T$ is embedded within an average over edges. However, if it is the case that $\left( 1+\frac{\tau_{j}^{2}}{\sigma_{\theta j}^{2}} \right)^{-1}$ varies little relative to $\rho_{j}^{2}$, then we might be able to fit observed correlations as a function of $N$ and $T$ like

$\frac{K_{0}}{K_{0}+\frac{1}{N/2}\left( 1-2K_{1}\frac{1}{1+{K_{2}}/T} \right)}$ (S45)

where we can interpret $K_{0}$ as the inter-edge variability of true association $S_{\rho}^{2}$, $K_{1}$ as the joint influence of FC-phenotype association variability and phenotype reliability $R\left( Y \right)$, and $K_{2}$ as the normalized FC measurement error relative to true FC variance.

## References

Afyouni, S., Smith, S. M., & Nichols, T. E. (2019). Effective degrees of freedom of the Pearson's correlation coefficient under autocorrelation. *Neuroimage*, *199*, 609-625. <https://doi.org/10.1016/j.neuroimage.2019.05.011>

Gleser, L. J., Carroll, R. J., & Gallo, P. P. (1987). The Limiting Distribution of Least Squares in an Errors-in-Variables Regression Model. *The Annals of Statistics*, *15*(1), 220-233. <http://www.jstor.org/stable/2241078>

Spearman, C. (1904). The Proof and Measurement of Association between Two Things. *The American Journal of Psychology*, *15*(1), 72-101. <https://doi.org/10.2307/1412159>

# Supplemental material

## Supplementary Tables

### Supplementary Table 1. Statistical analyses.

This table summarizes all descriptive statistics and statistical tests supporting claims in this study. The table is organized into different sub-sections to mirror the main text results.

| Result section: Sample size & scan time interchangeability | | |
| --- | --- | --- |
| Context sentence | Procedure | Statistics / p value if any |
| Although cognitive factor scores are not necessarily comparable across datasets (due to population and phenotypic differences), prediction accuracies were highly similar between the ABCD and HCP datasets (Pearson’s r=0.98; Fig. 1b). | Pearson’s correlation was computed between prediction accuracies in the HCP dataset and prediction accuracies in the ABCD dataset for corresponding sample size and scan time per participant.  Note that 30 data points went into this correlation (30 dots in Fig. 1b. corresponding to prediction accuracies for a particular pair of sample size and scan time per participant) | r = 0.98  No p value can be computed because the samples are not independent. |
| Intriguingly, the prediction accuracy of the cognitive factor score increased with total scan duration (# training participants × scan time per participant) in both ABCD (Spearman’s ρ = 0.99) and HCP (Spearman’s ρ = 0.96) datasets (Fig. 2a). | We compute Spearman’s correlation between total scan duration and prediction accuracy.  90 data points went into this correlation for the ABCD dataset (90 dots in Fig. 2a left panel; each corresponding to prediction accuracy for a total scan duration).  174 data points went into this correlation for the HCP dataset, (174 dots in Fig 2a right panel; each corresponding to prediction accuracy for a total scan duration). | HCP: ρ = 0.96  ABCD: ρ = 0.99  No p value can be computed because the samples are not independent. However, we note that a statistical test was performed across all phenotypes when we correlated prediction accuracy with logarithm of total scan duration (see below). The statistical test provides further statistical evidence for this statement. |
| In both datasets, there were diminishing returns of sample size and scan time, whereby each unit increase in sample size or scan duration resulted in progressively smaller gains in prediction accuracy (Fig. 2a; Supplementary Table 1).  In the HCP dataset, we also observed diminishing returns of scan time relative to sample size, especially beyond 30 minutes (Fig. 2a; Supplementary Table 1). For example, starting from an accuracy of 0.33 with 200 participants × 14 min scans, a 3.5× larger sample (N = 700) lifted accuracy to 0.45, whereas a 4.1× longer scan (58 min) raised it only to 0.40. | We note that diminishing returns is demonstrated in the statistical test linking prediction accuracy with log of total scan duration. The theoretical model in the next section also formally characterizes this phenomenon as well. Therefore, here we will only illustrate these statements with numerical examples.  As an example of diminishing returns of both sample size and scan time, we can consider the ABCD cognitive factor, where accuracy was 0.37 (N = 400; T = 4). Increasing the sample size by another 400 (N = 800, T = 4) improved accuracy by 0.02. However, we need to increase sample size by another 1000 (N = 1800, T = 4) to further improve accuracy by 0.05. This demonstrates diminishing returns of sample size.  As an example of diminishing returns in scan time, for the HCP cognitive factor score, accuracy (N = 200; T = 14) was 0.33. Increasing scan time by 14 min (N = 200; T = 28) led to an accuracy increase of 0.06. Increase the scan time by another 30 min (N = 200; T = 58) led to a small accuracy increase of 0.01. This demonstrates diminishing returns of scan time.  As an example of diminishing returns of scan time relative to sample size, for the HCP cognitive factor score (N = 200; T = 14), accuracy was 0.33. A 3.5× larger sample (N = 700) lifted accuracy to 0.45, whereas a 4.1× longer scan (58 min) raised it only to 0.40. | HCP (cognitive factor)  200 × 14 min: 0.33  400 × 14 min: 0.40  200 × 28 min: 0.39  700 × 14 min: 0.45  200 × 58 min: 0.40  ABCD (cognitive factor)  400 × 4 min: 0.37  800 × 4 min: 0.39  400 × 8 min: 0.41  1800 × 4 min: 0.44  400 × 20 min: 0.42 |
| 90% of HCP phenotypes (i.e., 26 out of 29) and 100% of ABCD phenotypes (i.e., 23 out of 23) exhibited prediction accuracies that increased with total scan duration (Spearman’s ρ = 0.85). | For each phenotype, Spearman’s correlation between prediction accuracies and total scan durations was computed, and then averaged across all 26+23 = 49 phenotypes. | ρ = 0.85  No p value can be computed because the samples are not independent.  However, we note that a statistical test was performed across all phenotypes when we correlated prediction accuracy with logarithm of total scan duration (see below). The statistical test provides further statistical evidence for this statement. |
| Diminishing returns of scan time (relative to sample size) was observed for many HCP phenotypes, especially beyond 20 minutes (Supplementary Table 1). This phenomenon was less pronounced for the ABCD phenotypes, potentially because maximum scan time was only 20 minutes (Supplementary Table 1). | We note that a statistical test of diminishing returns of scan time relative to sample size is demonstrated in the next section (Fig. 3a). Furthermore, the theoretical model in the next section also formally characterizes this phenomenon as well. Therefore, here we will only illustrate these statements with numerical examples.  As an example of diminishing returns of scan time relative to sample size, for the HCP cognitive factor score, accuracy (N = 200; T = 14) was 0.11. A 3.5× larger sample (N = 700) lifted accuracy to 0.19, whereas a 4.1× longer scan (58 min) raised it only to 0.14.  In the case of ABCD, accuracy (N = 400, T = 4) was 0.16. A 4.5× larger sample size (N =1800) lifted accuracy to 0.21, while a 5× increase in scan time only increased accuracy to 0.20. So slight diminishing returns was also observed for ABCD, consistent with results (Extended Data Fig. 3a) and theoretical model from the next section. | HCP (26 phenotypes)  200 × 10 min: 0.11  400 × 10 min: 0.15  200 × 20 min: 0.13  700 × 10 min: 0.19  200 × 58 min: 0.14  ABCD (23 phenotypes)  400 × 4 min: 0.16  800 × 4 min: 0.18  400 × 8 min: 0.19  1800 × 4 min: 0.21  400 × 20 min: 0.20 |
| Overall, total scan duration explained prediction accuracy across HCP and ABCD phenotypes remarkably well: coefficient of determination (COD) or R^2^ = 0.88 and 0.89 respectively (Supplementary Table 3). | For each phenotype, we fitted the log curve, and then the coefficient of determinant (COD; R^2^) was computed based on the fit. The CODs were then averaged across all 19 HCP phenotypes and 17 ABCD phenotypes. | HCP: R^2^ = 0.88  ABCD: R^2^ = 0.89 |
| The logarithm of total scan duration explained prediction accuracy very well (r = 0.95; p = 0.001). | In Fig. 2b, we calculate the Pearson’s correlation between the log of total scan duration and normalized prediction performance. The correlation was computed based on all 2520 dots in Fig. 2b Each dot refers to prediction accuracy for a specific total scan duration (16 total scan duration x 90 ABCD phenotypes + 18 total scan duration x 60 HCP phenotypes = 2520).  To compute a p value, we note that the dots in Fig. 2b are not independent across different total scan durations. Furthermore, the cognitive factor scores were derived from other measures, so we excluded the two cognitive factor scores from the 36 phenotypes, resulting in 34 phenotypes.  For each of the remaining 34 phenotypes, we randomly picked a total scan duration (corresponding to a particular scan time and sample size) and corresponding normalized prediction accuracy. Therefore, we ended up with 34 pairs of log_2_(total scan duration) and normalized prediction performance. We then use a permutation test (1,000 permutations) to obtain a p value for the Pearson’s correlation between the 34 pairs of values.  This procedure was repeated 100 times to ensure robustness, so we ended up with 100 p values (which all turned out to be 0.001). | r = 0.95  All 100 p values = 0.001 |
| Result section: Diminishing returns of scanning longer  Multiple comparisons were corrected using the Benjamini–Yekutieli false discovery rate (FDR) procedure with q < 0.05. The FDR procedure was applied to all p values in this subsection. | | |
| Context sentence | Procedure | Statistics / p value if any |
| Prediction accuracy decreased with increasing scan time per participant, despite maintaining 6000 minutes of total scan duration (Fig. 3a). However, the accuracy reduction was modest for short scan times (Fig. 3a; Supplementary Table 1). | A corrected resampled two-tailed paired-sample t-test was performed between the largest sample size (N = 600, T = 10 min) and other sample sizes in Fig. 3a.  The corrected resampled t-test (Nadeau & Bengio, 2003) corrects for the non-independence across the 50 repetitions of 10-fold cross-validation (due to overlapping training and test sets across splits).  Note that there were 50 x 10 = 500 prediction accuracy values for each sample size (and scan time). Each corrected resampled t-test was performed on 500 pairs of prediction accuracy values. | 10m vs 20m: p = 0.48  10m vs 30m: p = 0.21  10m vs 40m: p = 0.054  10m vs 50m: p = 7.9e-3*  10m vs 58m: p = 9.8e-4*  * indicates significance after FDR correction (q < 0.05) |
| Similar conclusions were obtained for all 19 HCP and 17 ABCD phenotypes that followed a logarithmic fit (Extended Data Fig. 3). | Two-tailed paired-sample t-test was performed between the largest sample size and the other sample sizes.  Same as before, the cognitive factor scores were excluded when performing the tests. Therefore, each HCP test involved 18 pairs of values, while each ABCD test involved 16 pairs of values. | ABCD  4 vs 10 min: p = 7.7e-9*  4 vs 20 min: p = 4.9e-7*  HCP  10 vs 20 min: p = 0.0006*  10 vs 30 min: p = 1.7e-5*  10 vs 40 min: p = 4.9e-7*  10 vs 50 min: p = 9.4e-8*  10 vs 58 min: p = 2.0e-8*  * indicates significance FDR correction (q < 0.05) |
| For 20-min scans, the logarithmic and theoretical models performed equally well with equivalent goodness of fit (R^2^) across the 17 ABCD phenotypes (p = 0.57; Supplementary Table 1). For longer scan time, the theoretical model exhibited better fit than the logarithmic model across the 19 HCP phenotypes (p = 0.002; Supplementary Table 1; Supplementary Fig. 12). | Two-tailed paired-sample t-test was performed between goodness of fit (COD) of logarithm and theoretical models.  Like before the cognitive factor scores were excluded when performing the tests. Therefore, HCP test involved 18 pairs of values, while ABCD test involved 16 pairs of values. | ABCD: p = 0.57  HCP: p = 0.002*  * indicates significance after FDR correction (q < 0.05) |
| Result section: Predictability increases model adherence  Multiple comparisons were corrected using the Benjamini–Yekutieli false discovery rate (FDR) procedure with q < 0.05. The FDR procedure was applied to all p values in this subsection. | | |
| Context sentence | Procedure | Statistics / p value if any |
| Indeed, model fit was strongly correlated with prediction accuracy across phenotypes in both datasets (Spearman’s ρ = 0.90; p = 0.001; Extended Data Figs. 4c and 4d). | For each dataset, we performed Spearman’s correlation between goodness of fit (COD) of theoretical model and prediction accuracy (corresponding obtained from maximum sample size and scan time) across phenotypes.  In ABCD, the correlation was based on 33 phenotypes. In HCP, the correlation was based on 42 phenotypes.  To obtain a p value, we performed a permutation test with 10,000 permutations. The cognitive factor scores were excluded in the permutation test. Thus, the permutation was based on 32 ABCD phenotypes and 41 HCP phenotypes. | ABCD: ρ = 0.9, p = 0.001*  HCP: ρ = 0.9, p = 0.001*  * indicates significance after FDR correction (q < 0.05) |
| Result section: Non-stationarity weakens model adherence  Multiple comparisons were corrected using the Benjamini–Yekutieli false discovery rate (FDR) procedure with q < 0.05. The FDR procedure was applied to all p values in this subsection. | | |
| Context sentence | Procedure | Statistics / p value if any |
| For example, “Anger: Aggression” was reasonably well-predicted in the HCP dataset. While prediction accuracy increased with larger sample sizes (Spearman’s ρ = 1.00), extending scan duration did not generate a similarly consistent effect for this phenotype (Spearman’s ρ = 0.21; Extended Data Fig. 5a). | For each sample size N, we calculate Spearman’s correlation between the prediction accuracies and the entire range of scan times (2 to 58 min). 29 pairs of values went into each correlation. We then average the correlations across all sample sizes.  We repeat by reversing the role of scan time and sample size. More specifically, for each scan time T, we calculate Spearman’s correlation between the prediction accuracies and the entire range of sample sizes. 6 pairs of values went into each correlation. We then average the correlations across all scan times. | Before randomization Average ρ between accuracy and sample size (from 200 to 700*) = 1.00  Average ρ between accuracy and scan time (from 2 min to 58 min): 0.21  After randomization  Average ρ between accuracy and sample size (from 200 to 700*) = 1.00  Average ρ between accuracy and scan time (from 2 min to 58 min): 0.98  * indicates that the sample size is not exactly 700 (see main text).  No p value can be computed because the samples are not independent. |
| The run randomization improved the goodness of fit of the theoretical model (p < 4e-5), suggesting the presence of non-stationarities (Extended Data Figs. 5b and 5c). | Two-tail paired-sample t-test between the CODs before and after randomization.  The statistical test for ABCD used 32 pairs of CODs because there were 32 phenotypes (after excluding cognitive factor score).  The statistical test for HCP used 41 pairs of CODs because there were 41 phenotypes (after excluding cognitive factor score). | ABCD: p = 8.8e-5* HCP: p = 2.23e-7*  * indicates significance FDR correction (q < 0.05) |
| Result section: Higher overhead costs favor longer scans | | |
| Context sentence | Procedure | Statistics / p value if any |
| We fitted the theoretical model to 76 phenotypes in the nine datasets, yielding an average COD or R^2^ of 89% (Supplementary Table 1). | We fitted the theoretical model to each phenotype (see Methods), and then computed resulting goodness of fit (COD). We then averaged the COD across the 76 phenotypes. | R^2^ = 0.89  No p value was computed because the samples were not independent. |
| We note that the Pearson’s correlation between Fig. 4a and Fig. 1a across corresponding sample sizes and scan durations was 0.97 (Supplementary Table 1). | Pearson’s correlation was computed between prediction accuracies in Fig. 4a and Fig. 1a (ABCD) for corresponding sample size and scan time per participant. There were 90 pairs of values that went into this correlation.  For completeness, we also computed Pearson’s correlation between prediction accuracies in Fig. 4a and Supplementary Fig. 1 (HCP) for corresponding sample size and scan time per participant. There were 174 pairs of values that went into this correlation. | ABCD: r = 0.97  HCP: r = 0.99  No p value can be computed because the samples are not independent. |
| The asymmetry of the curves suggests that it is better to overshoot than undershoot optimal scan time (Supplementary Table 1). | To quantify this phenomenon, recall that there are 36 curves in Extended Data Fig. 6.  For each curve, we computed the fraction of maximum prediction accuracies 10 min before and 10 min after the optimal scan time, thus yielding 36 pairs of values.  We then computed the absolute change in prediction accuracies relative from when using optimum can time across the 36 values (10 minutes before and 10 after optimal scan time). Larger numbers denote greater loss in prediction accuracy.  Observe that the prediction accuracies were reduced more at 10 mins below optimal scan time compared with 10 mins beyond optimal scan time. | Reduction in prediction accuracy 10 mins before optimal scan time =  mean = 0.0041; std = 0.0059  Reduction in prediction accuracy 10 mins after optimal scan time =  mean = 0.0026; std = 0.0028  No p value can be computed because the samples are not independent. |
| Result section: 30-min scans are the most cost-effective | | |
| Context sentence | Procedure | Statistics / p value if any |
| On average across resting and task states, 30-min scans were the most cost-effective (95% bootstrapped confidence interval (CI) 25-40; Extended Data Fig. 7; Supplementary Table 1) | To derive the confidence interval, we performed bootstrap. More specifically, since there were 9 datasets, we sampled 9 datasets with replacement. Within each dataset, we sampled the phenotypes with replacement. Like before, the cognitive factor scores were excluded from this bootstrap.  We then used the bootstrapped samples to repeat the same analysis (used to derive Fig. 4c), yielding the most cost-effective scan time. We repeated the bootstrap procedure 1000 times, yielding 1000 most cost-effective scan times. 95% confidence intervals were then obtained by sorting the 1000 values and finding the 2.5th percentile and 97.5th percentile most-cost-effective scan time. | 95% CI = (25, 40) |
| Results section: Minimizing task-fMRI costs | | |
| Context sentence | Procedure | Statistics / p value if any |
| Across the six resting-state datasets (Fig. 5a), the most cost-effective scan time was the longest for ABCD (60 min; CI 40-100) and shortest for the TCP and ADNI datasets (20 min; TCP CI 10-35; ADNI CI 15-35).  the most cost-effective scan time was shorter for ABCD task-fMRI than ABCD resting-state fMRI (Fig. 5b; Supplementary Table 1). Among the three tasks, the most cost-effective scan time was the shortest for n-back at 25 min (CI 20-35) | To derive the confidence interval, we performed bootstrap. This procedure was repeated independently for each of 9 datasets.  For each dataset, we sampled the phenotypes with replacement. Like before, the cognitive factor scores were excluded from this bootstrap. We then used the bootstrapped samples to repeat the same analysis (used to derive Fig. 5a or Fig. 5b).  We repeated the bootstrap procedure 1000 times, yielding 1000 most cost-effective scan times. 95% confidence intervals were then obtained by sorting the 1000 values and finding the 2.5% percentile and 97.5% percentile most-cost-effective scan time. | 95% confidence intervals:  ABCD-RS: (40, 100)  ABCD-MID: (30, 55)  ABCD-NBACK: (20, 35)  ABCD-SST: (20, 50)  HCP: (25, 40)  SINGER: (25, 55)  TCP: (10, 35)  MDD: (15, 65)  ADNI: (15, 35) |
| The most cost-effective scan time for the two-session design was only slightly longer than for the original HCP analysis (Fig. 5c): 40 min (CI 30-55) vs 30 min (CI 25-40). | Bootstrap procedure was the same as above. For each condition (original or two-session), we sampled the phenotypes with replacement. Like before, the cognitive factor scores were excluded from this bootstrap. We then used the bootstrapped samples to repeat the same analysis (used to derive Fig. 5c)  We repeated the bootstrap procedure 1000 times, yielding 1000 most cost-effective scan times. 95% confidence intervals were then obtained by sorting the 1000 values and finding the 2.5% percentile and 97.5% percentile most-cost-effective scan time. | 95% confidence intervals:  HCP original: 30 min (25, 40)  HCP two session: 40 min (30, 55) |
| Results section: Variation across phenotypes & scan parameters | | |
| Context sentence | Procedure | Statistics / p value if any |
| we did not find an obvious relationship between phenotypic prediction accuracy and optimal scan time (Extended Data Fig. 8b; Supplementary Table 1) | To quantify this, we note that there were three boxplots corresponding to prediction accuracies (1) r < 0.25, (2) 0.25 < r < 0.5 and (3) r > 0.5  For each boxplot, we computed the median optimal scan time. Because optimal scan time decreases then increases, we conclude that there was not an obvious relationship between accuracy and optimal scan time.  Note that we were not trying to claim that there were (or were no) differences between boxplots, so we did not perform a statistical test or equivalence test between boxplots. Instead, we were simply making the observation that the optimal scan time was not monotonically increasing or decreasing over the conditions. | Median optimal scan time (min): 39 **→ 37 → 39** |
| there was not an obvious relationship between phenotypic test-retest reliability and optimal scan time (Extended Data Fig. 8c; Supplementary Table 1) | To quantify this, we note that there were three boxplots corresponding to test-retest reliability (1) ICC < 0.5, (2) 0.5 < ICC < 0.75 and (3) ICC > 0.75  For each boxplot, we computed the median optimal scan time. Because optimal scan time decreased then increased, we concluded that there was not an obvious relationship between phenotypic reliability and optimal scan time.  Note that we were not trying to claim that there were (or were not) differences between boxplots, so we did not perform a statistical test or equivalence test between boxplots. Instead, we were just making the observation that the optimal scan time was not monotonically increasing or decreasing over the conditions. | Median optimal scan time (min): 35 **→ 30 → 33** |
| There was also not an obvious relationship between optimal scan time and temporal resolution, voxel resolution or scan sequence (Extended Data Figs. 8d to 8f; Supplementary Table 1). | To quantify this for temporal resolution (TR), we note that there were five boxplots corresponding to TR = 0.7s, 0.8s, 0.8s, 1s and 3s. For each boxplot, we computed the median optimal scan time. Because optimal scan time went up and down, we concluded that there was not an obvious relationship between temporal resolution and optimal scan time.  To quantify this for voxel resolution, we note that there were five boxplots corresponding to voxel size = 2mm, 2mm, 2.4mm, 3mm and 3mm. For each boxplot, we computed the median optimal scan time. Because optimal scan time was the highest of the middle voxel resolution, we concluded that there was not an obvious relationship between voxel resolution and optimal scan time.  To quantify this for scan acquisition, we note that there were three boxplots corresponding to single-echo single-band (SE-SB), single-echo multi-band (SE-MB), and multi-echo multi-band (ME-MB). We might think of the acquisition quality improving from SE-SB to SE-MB to ME-MB. For each boxplot, we computed the median optimal scan time. Because optimal scan time was the highest of the middle acquisition, we concluded that there was not an obvious relationship between acquisition and optimal scan time.  Note that we were not trying to claim that there were (or were not) differences between boxplots, so we did not perform a statistical test or equivalence test between boxplots. Instead, we were just making the observation that the optimal scan time was not monotonically increasing or decreasing over the conditions. | Median optimal scan time (min) for TR: 32 **→** 59 **→** 20 **→** 34 **→** 28  Median optimal scan time (min) for voxel resolution:  32 **→** 20 **→** 59 **→** 34 **→** 28  Median optimal scan time (min) for acquisition type: 28 **→** 35 **→** 34 |
| Results section: Minimizing costs of subcortical BWAS | | |
| Context sentence | Procedure | Statistics / p value if any |
| The most cost-effective scan time for subcortical RSFC was about double that of whole-brain RSFC (Fig. 6a; Supplementary Table 1; Extended Data Fig. 7). | Bootstrap procedure is the same as above. For each condition (whole-brain FC or subcortical-to-whole-brain FC), we sampled the phenotypes with replacement. Like before, the cognitive factor scores were excluded from this bootstrap. We then used the bootstrapped samples to repeat the same analysis (used to derive Fig. 5c)  We repeated the bootstrap procedure 1000 times, yielding 1000 most cost-effective scan times. 95% confidence intervals were then obtained by sorting the 1000 values and finding the 2.5% percentile and 97.5% percentile most-cost-effective scan time. | 95% confidence intervals:  ABCD whole-brain: (35, 80)  ABCD subcortical: (65, 395)  HCP whole-brain: (25, 40)  HCP subcortical: (45, 80) |
| Even doubling the noise ($\sigma$ = 1) had very little impact on the optimal scan time (Fig. 6b; Supplementary Table 1). As a sanity check, we added a large quantity of noise ($\sigma$ = 3), which led to a much longer optimal scan time (Fig. 6b; Supplementary Table 1). | In Fig. 6b, we note that for each dataset, there were 12 conditions, corresponding 3 possible accuracy targets (80%, 90% or 95% of maximum achievable accuracy), 2 possible overhead costs ($500 or $1000 per participant) and 2 possible scan costs per hour ($500 or $1000). So there were 3 × 2 × 2 = 12 conditions.  Note that we could not bootstrap the phenotypes (like previous analyses) since this analysis only involved the cognitive factor scores. In addition, there were only 4 values of $\sigma$, so we also decided against correlating optimal scan time with $\sigma$. The 12 curves were also not independent, so we could not treat them as independent to perform a statistical test.  Instead, we simply averaged the 12 curves for each dataset and reported the optimal scan time for each value of $\sigma$ (see next column). We observed that from $\sigma$ = 0 (no noise) to $\sigma$ = 1 (doubling the noise), there was no change in optimal scan time for HCP and ~5 min increase in optimal scan time for ABCD. However, there was a large increase in optimal scan time when we increased $\sigma$ = 1 to $\sigma$ = 3. | HCP (min)  $\sigma$ = 0.0: Optimal T = 33.8  $\sigma$ = 0.5: Optimal T = 31.0  $\sigma$ = 1.0: Optimal T = 33.8  $\sigma$ = 3.0: Optimal T = 83.1  ABCD (min)  $\sigma$ = 0.0: Optimal T = 57.0  $\sigma$ = 0.5: Optimal T = 57.0  $\sigma$ = 1.0: Optimal T = 61.8  $\sigma$ = 3.0: Optimal T = 394.7 |
| There was a weak trend in which higher parcellation resolution led to slightly lower optimal scan time, although there was a big drop in optimal scan time from 200 parcels to 400 parcels in the ABCD dataset (Fig. 6c; Supplementary Table 1). | In Fig. 6c, we note that for each dataset, there were 12 conditions, corresponding 3 possible accuracy targets (80%, 90% or 95% of maximum achievable accuracy), 2 possible overhead costs ($500 or $1000 per participant) and 2 possible scan costs per hour ($500 or $1000). So there were 3 × 2 × 2 = 12 conditions.  Note that we could not bootstrap the phenotypes (like previous analyses) since this analysis only involved the cognitive factor scores (Fig. 6c). In addition, there were only 5 parcellation resolutions, so we also decided against correlating optimal scan time with the number of parcels. The 12 curves were also not independent, so we cannot treat them as independent to perform a statistical test.  Instead, we simply averaged the 12 curves for each dataset and reported the optimal scan time for each parcellation resolution (see next column).  In the HCP dataset, there was a trend of decreasing optimal scan time from 200 to 800 parcels. But reduction was small and less than 10 minutes. From 800 to 1000 parcels, the optimal scan time only increased by 3 min.  In the ABCD dataset, there was a big drop in optimal scan time from 200 to 400 parcels. But optimal scan time stayed the same from 400 to 600 parcels and then decreased by 17 min from 600 to 800 parcels, and then stayed the same from 800 to 1000 parcels. | HCP (min)  200: Optimal T = 35.5 min  400: Optimal T = 33.8 min  600: Optimal T = 28.4 min  800: Optimal T = 28.4 min  1000: Optimal T = 31.1 min  ABCD (min)  200: Optimal T = 395.0 min  400: Optimal T = 57.0 min  600: Optimal T = 57.0 min  800: Optimal T = 40.2 min  1000: Optimal T = 40.2 min |

Supplementary Table 2. Summary of prediction accuracy analyses in the ABCD and HCP datasets.

Table S1a shows the number of phenotypes in the prediction accuracy analyses across phenotypic domains. The “loose accuracy threshold” column shows the number of phenotypes whose prediction accuracies (Pearson’s r) were positive in at least 90% of all combinations of sample size *N* and scan time *T*. The “strict accuracy threshold” column shows the number of phenotypes whose prediction accuracies (Pearson’s r) were more than 0.1 when the full dataset was used (maximum *N* and *T*). The “Diminishing returns” column shows the number of phenotypes (among the subset of scores than passed the strict threshold) that exhibited diminishing returns in prediction accuracy with more than 20 minutes of scan time (relative to sample size). The “Exhibit log relationship” column shows the number of phenotypes (among the diminishing returns phenotypes) that displayed a possible logarithmic relationship between prediction accuracy and total scan duration. Table S1b shows the goodness-of-fit for the theoretical prediction accuracy models before and after randomizing run order. Goodness-of-fit was measured using the coefficient of determination (COD), which ranges from 0 to 1, which can be thought of fraction of variance explained. The COD was averaged across all phenotypes in the “loose accuracy threshold”, “strict accuracy threshold” and “exhibit log relationship”.

| S1a. Number of phenotypes in the prediction accuracy analyses | | | | | | | | | |
| --- | --- | --- | --- | --- | --- | --- | --- | --- | --- |
| Dataset | Phenotypic Domain | Total | Loose threshold (at least 90% with r > 0) | Strict threshold (max r > 0.1) | | Diminishing returns at 20 mins | | Exhibit log relationship | |
| ABCD | All | 37 | 33 (out of 37) | 23 (out of 37) | | 23 (out of 23) | | 17 (out of 23) | |
|  | Cognition | 17 | 17 (out of 17) | 15 (out of 17) | | 15 (out of 15) | | 13 (out of 15) | |
|  | Personality | 9 | 6 (out of 9) | 3 (out of 9) | | 3 (out of 3) | | 0 (out of 3) | |
|  | Mental Health | 11 | 10 (out of 11) | 5 (out of 11) | | 5 (out of 5) | | 4 (out of 5) | |
| HCP | All | 59 | 42 (out of 59) | 29 (out of 59) | | 26 (out of 29) | | 19 (out of 26) | |
|  | Cognition | 20 | 18 (out of 20) | 16 (out of 20) | | 15 (out of 16) | | 12 (out of 14) | |
|  | Personality | 6 | 5 (out of 6) | 4 (out of 6) | | 4 (out of 4) | | 4 (out of 4) | |
|  | Emotion | 13 | 6 (out of 13) | 1 (out of 13) | | 0 (out of 1) | | 1 (out of 1) | |
|  | Physical | 9 | 7 (out of 9) | 5 (out of 9) | | 5 (out of 5) | | 1 (out of 5) | |
|  | Well-being | 11 | 6 (out of 11) | 3 (out of 11) | | 2 (out of 3) | | 1 (out of 2) | |
|  | | | | | | | | | |
| S1b. Average goodness-of-fit (COD or R^2^) over each set of phenotypes for prediction models before and after randomizing run orders | | | | | | | | | |
|  | | | | 20 min | 58 min | | 20 min (random) | | 58 min (random) |
| ABCD (Loose accuracy threshold: 33 phenotypes) | | | |  |  | |  | |  |
| Theoretical | | | | 0.763 | N.A | | 0.844 | | N.A |
| ABCD (Strict accuracy threshold: 23 phenotypes) | | | |  |  | |  | |  |
| Theoretical | | | | 0.834 | N.A | | 0.885 | | N.A |
| ABCD (Exhibit log relationship: 17 phenotypes) | | | |  |  | |  | |  |
| Theoretical | | | | 0.894 | N.A | | 0.940 | | N.A |
| HCP (Loose accuracy threshold: 42 phenotypes) | | | |  |  | |  | |  |
| Theoretical | | | | 0.724 | 0.728 | | 0.854 | | 0.880 |
| HCP (Strict accuracy threshold: 28 phenotypes) | | | |  |  | |  | |  |
| Theoretical | | | | 0.818 | 0.836 | | 0.918 | | 0.926 |
| HCP (Exhibit log relationship: 19 phenotypes) | | | |  |  | |  | |  |
| Theoretical | | | | 0.890 | 0.888 | | 0.945 | | 0.947 |

### Supplementary Table 3. Summary of prediction accuracy analyses for all datasets.

The “strict accuracy threshold” column shows the number of phenotypes whose prediction accuracies (Pearson’s r) were more than 0.1 when the full dataset was used (maximum *N* and *T*). The “Adherence to theoretical model” column shows the number of phenotypes which passed the strict accuracy threshold and showed a good fit to the theoretical models after visual assessment. “Average COD of model fit” refers to the goodness-of-fit of the theoretical model averaged over the phenotypes that showed adherence to the theoretical model. We note that many phenotypes overlap between ABCD (rest), ABCD (MID), ABCD (NBACK), ABCD (SST), so in the case of the ABCD, there were in total 23 unique phenotypes that showed adherence to the theoretical model, yielding 23 + 19 + 14 + 7 + 7 + 6 = 76 unique phenotypes used for generating Fig. 4a.

| Dataset | Total number of phenotypes | Strict accuracy threshold (max r > 0.1) | Adherence to theoretical model | Average COD of model fit |
| --- | --- | --- | --- | --- |
| ABCD (rest) | 37 | 23 (out of 37) | 17 (out of 23) | 0.894 |
| HCP | 59 | 28 (out of 59) | 19 (out of 28) | 0.888 |
| SINGER | 19 | 15 (out of 19) | 14 (out of 15) | 0.926 |
| TCP | 19 | 10 (out of 19) | 7 (out of 10) | 0.818 |
| MDD | 20 | 11 (out of 20) | 7 (out of 11) | 0.844 |
| ADNI | 6 | 6 (out of 6) | 6 (out of 6) | 0.920 |
| ABCD (MID) | 37 | 21 (out of 37) | 16 (out of 21) | 0.921 |
| ABCD (NBACK) | 37 | 22 (out of 37) | 19 (out of 22) | 0.884 |
| ABCD (SST) | 37 | 22 (out of 37) | 18 (out of 22) | 0.872 |
| Control Analyses |  |  |  |  |
| ABCD (subcortical) | 37 | 18 (out of 37) | 14 (out of 18) | 0.868 |
| HCP (subcortical) | 59 | 21 (out of 59) | 13 (out of 21) | 0.860 |
| HCP (two-sessions) | 59 | 21 (out of 59) | 16 (out of 21) | 0.847 |

### Supplementary Tables 4.1-4.6. Phenotypic Measures in each dataset.

Supplementary Table 4.1 Phenotypic measures in the ABCD dataset.

|  | Description | ABCD field | ABCD file | Category |
| --- | --- | --- | --- | --- |
| 1 | Anxious Depressed | cbcl_scr_syn_anxdep_r | abcd_cbcls01.txt | Mental Health |
| 2 | Withdrawn Depressed | cbcl_scr_syn_withdep_r | abcd_cbcls01.txt | Mental Health |
| 3 | Somatic Complaints | cbcl_scr_syn_somatic_r | abcd_cbcls01.txt | Mental Health |
| 4 | Social Problems | cbcl_scr_syn_social_r | abcd_cbcls01.txt | Mental Health |
| 5 | Thought Problems | cbcl_scr_syn_thought_r | abcd_cbcls01.txt | Mental Health |
| 6 | Attention Problems | cbcl_scr_syn_attention_r | abcd_cbcls01.txt | Mental Health |
| 7 | Rule-breaking Behavior | cbcl_scr_syn_rulebreak_r | abcd_cbcls01.txt | Mental Health |
| 8 | Aggressive Behavior | cbcl_scr_syn_aggressive_r | abcd_cbcls01.txt | Mental Health |
| 9 | Vocabulary | nihtbx_picvocab_uncorrected | abcd_tbss01.txt | Cognition |
| 10 | Attention | nihtbx_flanker_uncorrected | abcd_tbss01.txt | Cognition |
| 11 | Working Memory | nihtbx_list_uncorrected | abcd_tbss01.txt | Cognition |
| 12 | Executive Function | nihtbx_cardsort_uncorrected | abcd_tbss01.txt | Cognition |
| 13 | Processing Speed | nihtbx_pattern_uncorrected | abcd_tbss01.txt | Cognition |
| 14 | Episodic Memory | nihtbx_picture_uncorrected | abcd_tbss01.txt | Cognition |
| 15 | Reading | nihtbx_reading_uncorrected | abcd_tbss01.txt | Cognition |
| 16 | Fluid Cognition | nihtbx_fluidcomp_uncorrected | abcd_tbss01.txt | Cognition |
| 17 | Crystallized Cognition | nihtbx_cryst_uncorrected | abcd_tbss01.txt | Cognition |
| 18 | Overall Cognition | nihtbx_totalcomp_uncorrected | abcd_tbss01.txt | Cognition |
| 19 | Negative Urgency | upps_y_ss_negative_urgency | abcd_mhy02.txt | Personality |
| 20 | Lack of Planning | upps_y_ss_lack_of_planning | abcd_mhy02.txt | Personality |
| 21 | Sensation Seeking | upps_y_ss_sensation_seeking | abcd_mhy02.txt | Personality |
| 22 | Positive Urgency | upps_y_ss_positive_urgency | abcd_mhy02.txt | Personality |
| 23 | Lack Perseverance | upps_y_lack_of_perseverance | abcd_mhy02.txt | Personality |
| 24 | Behavioral Inhibition | bis_y_ss_bis_sum | abcd_mhy02.txt | Personality |
| 25 | Reward Responsiveness | bis_y_ss_bas_rr | abcd_mhy02.txt | Personality |
| 26 | Drive | bis_y_ss_bas_drive | abcd_mhy02.txt | Personality |
| 27 | Fun Seeking | bis_y_ss_bas_fs | abcd_mhy02.txt | Personality |
| 28 | Total Psychosis Symptoms | pps_y_ss_number | abcd_mhy02.txt | Mental Health |
| 29 | Psychosis Severity | pps_y_ss_severity_score | abcd_mhy02.txt | Mental Health |
| 30 | Mania | pgbi_p_ss_score | abcd_mhp02.txt | Mental Health |
| 31 | Short Delay Recall | pea_ravlt_sd_trial_vi_tc | abcd_ps01.txt | Cognition |
| 32 | Long Delay Recall | pea_ravlt_ld_trial_vii_tc | abcd_ps01.txt | Cognition |
| 33 | Fluid Intelligence | pea_wiscv_trs | abcd_ps01.txt | Cognition |
| 34 | Visuospatial Accuracy | lmt_scr_perc_correct | lmtp201.txt | Cognition |
| 35 | Visuospatial Reaction Time | lmt_scr_rt_correct | lmtp201.txt | Cognition |
| 36 | Visuospatial Efficiency | lmt_scr_efficiency | lmtp201.txt | Cognition |
| 37 | Cognitive factor score | Obtained from PCA of previous 36 measures | N.A. | Cognition |

Supplementary Table 4.2 Phenotypic measures in the HCP dataset.

|  | Description | HCP field | Category |
| --- | --- | --- | --- |
| 1 | Visual Episodic Memory | PicSeq_Unadj | Cognition |
| 2 | Cognitive Flexibility (DCCS) | CardSort_Unadj | Cognition |
| 3 | Inhibition (Flanker Task) | Flanker_Unadj | Cognition |
| 4 | Fluid Intelligence (PMAT) | PMAT24_A_CR | Cognition |
| 5 | Reading (Pronunciation) | ReadEng_Unadj | Cognition |
| 6 | Vocabulary (Picture Matching) | PicVocab_Unadj | Cognition |
| 7 | Processing Speed | ProcSpeed_Unadj | Cognition |
| 8 | Delay Discounting | DDic_AUC_40K | Personality |
| 9 | Spatial Orientation | VSPLOT_TC | Cognition |
| 10 | Sustained Attention – Sens. | SCPT_SEN | Cognition |
| 11 | Sustained Attention – Spec. | SCPT_SPEC | Cognition |
| 12 | Verbal Episodic Memory | IWRD_TOT | Cognition |
| 13 | Working Memory (List Sorting) | ListSort_Unadj | Cognition |
| 14 | Cognitive Status (MMSE) | MMSE_Score | Cognition |
| 15 | Sleep Quality (PSQI) | PSQI_Score | Physical |
| 16 | Walking Endurance | Endurance_Unadj | Physical |
| 17 | Walking Speed | GaitSpeed_Unadj | Physical |
| 18 | Manual Dexterity | Dexterity_Unadj | Physical |
| 19 | Grip Strength | Strength_Unadj | Physical |
| 20 | Odor Identification | Odor_Unadj | Physical |
| 21 | Pain Interference Survey | PainInterf_Tscore | Physical |
| 22 | Taste Intensity | Taste_Unadj | Physical |
| 23 | Contrast Sensitivity | Mars_Final | Physical |
| 24 | Emotional Face Matching | Emotion_Task_Face_Acc | Emotion |
| 25 | Arithmetic | Language_Task_Math_Avg_Difficulty_Level | Cognition |
| 26 | Story Comprehension | Language_Task_Story_Avg_Difficulty_Level | Cognition |
| 27 | Relational Processing | Relational_Task_Acc | Cognition |
| 28 | Social Cognition – Random | Social_Task_Perc_Random | Cognition |
| 29 | Social Cognition – Interaction | Social_Task_Perc_TOM | Cognition |
| 30 | Working Memory (N-back) | WM_Task_Acc | Cognition |
| 31 | Agreeableness (NEO) | NEOFAC_A | Personality |
| 32 | Openness (NEO) | NEOFAC_O | Personality |
| 33 | Conscientiousness (NEO) | NEOFAC_C | Personality |
| 34 | Neuroticism (NEO) | NEOFAC_N | Personality |
| 35 | Extraversion (NEO) | NEOFAC_E | Personality |
| 36 | Emot. Recog. – Total | ER40_CR | Emotion |
| 37 | Emot. Recog. – Angry | ER40ANG | Emotion |
| 38 | Emot. Recog. – Fear | ER40FEAR | Emotion |
| 39 | Emot. Recog. – Happy | ER40HAP | Emotion |
| 40 | Emot. Recog. - Neutral | ER40NOE | Emotion |
| 41 | Emot. Recog. – Sad | ER40SAD | Emotion |
| 42 | Anger – Affect | AngAffect_Unadj | Emotion |
| 43 | Anger – Hostility | AngHostil_Unadj | Emotion |
| 44 | Anger – Aggression | AngAggr_Unadj | Emotion |
| 45 | Fear – Affect | FearAffect_Unadj | Emotion |
| 46 | Fear – Somatic Arousal | FearSomat_Unadj | Emotion |
| 47 | Sadness | Sadness_Unadj | Emotion |
| 48 | Life Satisfaction | LifeSatisf_Unadj | Well-being |
| 49 | Meaning & Purpose | MeanPurp_Unadj | Well-being |
| 50 | Positive Affect | PosAffect_Unadj | Well-being |
| 51 | Friendship | Friendship_Unadj | Well-being |
| 52 | Loneliness | Loneliness_Unadj | Well-being |
| 53 | Perceived Hostility | PercHostil_Unadj | Well-being |
| 54 | Perceived Rejection | PercReject_Unadj | Well-being |
| 55 | Emotional Support | EmotSupp_Unadj | Well-being |
| 56 | Instrument Support | InstruSupp_Unadj | Well-being |
| 57 | Perceived Stress | PercStress_Unadj | Well-being |
| 58 | Self-Efficacy | SelfEff_Unadj | Well-being |
| 59 | Cognitive factor score | Obtained from PCA of previous 58 measures | Cognition |

Supplementary Table 4.3 Phenotypic measures in the SINGER dataset.

|  | Description | SINGER field | Category |
| --- | --- | --- | --- |
| 1 | Age | SC_age | Physical |
| 2 | Montreal Cognitive Assessment | SC_moca_TOTAL | Cognition |
| 3 | Years of Education | BL_Demo6b_eduyr | Cognition |
| 4 | Body Mass Index | BL_bmi | Physical |
| 5 | Grip Strength (Left) | BL_leftgrip_avg | Physical |
| 6 | Grip Strength (Right) | BL_rightgrip_avg | Physical |
| 7 | Mini-Mental State Examination | BL_MMSETotalScore | Cognition |
| 8 | Visual Paired Associates | BL_NTBVPAImmediateTotalScore | Cognition |
| 9 | Logical Memory | BL_NTBLogicalMemoryTotalScore | Cognition |
| 10 | Rey Auditory Visual Learning Test | BL_NTBRAVLTTotalScore | Cognition |
| 11 | Digit Span | BL_NTBDSTotalScore | Cognition |
| 12 | Category Fluency Test | BL_NTBCFTTotalScore | Cognition |
| 13 | Delayed visual paired associates | BL_NTBVPADelayTotalScore | Cognition |
| 14 | Delayed Logical Memory | BL_NTBLMDelayTotalScore | Cognition |
| 15 | Delayed Rey Auditory Visual Learning Test | BL_RAVLTdelayed_TOTAL | Cognition |
| 16 | Rey Auditory Visual Learning Test (Delay Recognition) | BL_NTBRAVLTDelayRecogTotalScore | Cognition |
| 17 | Trail Making Test Part A | BL_TMTPartASec | Cognition |
| 18 | Trail Making Test Part B | BL_TMTPartBSec | Cognition |
| 19 | Letter Digit Substitution | BL_LDSTTotalScore | Cognition |

Supplementary Table 4.4 Phenotypic measures in the TCP dataset.

|  | Description | TCP field | Category |
| --- | --- | --- | --- |
| 1 | Age | age | Physical |
| 2 | Columbia-Suicide Severity Rating Scale Ideation | cssrs_isi | Mental Health |
| 3 | Montgomery-Åsberg Depression Rating Scale | madrstot | Mental Health |
| 4 | Positive and Negative Syndrome Scale (General Psychopathology) | panss_gen_clean | Mental Health |
| 5 | Positive and Negative Syndrome Scale (Negative Symptoms) | panss_neg_clean | Mental Health |
| 6 | Positive and Negative Syndrome Scale (Positive Symptoms) | panss_pos_clean | Mental Health |
| 7 | Young Mania Rating Scale | ymrs_tot | Mental Health |
| 8 | Depression Anxiety Stress Scale (Anxiety) | dass_anx_sc | Mental Health |
| 9 | Depression Anxiety Stress Scale (Depression) | dass_depr_sc | Mental Health |
| 10 | Depression Anxiety Stress Scale (Stress) | dass_stress_sc | Mental Health |
| 11 | Depression Anxiety Stress Scale (Total) | dass_total | Mental Health |
| 12 | Marder Factor Score (Anxiety / Depression) | panss_marder_AnxDep | Mental Health |
| 13 | Marder Factor Score (Cognitive / Disorganisation) | panss_marder_CogDis | Mental Health |
| 14 | Marder Factor Score (Negative Symptoms) | panss_marder_Neg | Mental Health |
| 15 | Marder Factor Score (Positive Symptoms) | panss_marder_Pos | Mental Health |
| 16 | Marder Factor Score (Uncontrolled Hostility / Excitement) | panss_marder_UHE | Mental Health |
| 17 | Positive and Negative Syndrome Scale (Total) | panss_tot | Mental Health |
| 18 | Perceived Stress | pss_totalscore | Mental Health |
| 19 | Body Mass Index | bmi | Physical |

Supplementary Table 4.5 Phenotypic measures in the MDD dataset.

|  | Description | MDD field | Category |
| --- | --- | --- | --- |
| 1 | Age | Age | Physical |
| 2 | Hamilton Depression Rating Scale (Total) | HAMD_baseline_total | Mental Health |
| 3 | Hamilton Anxiety Rating Scale (Total) | HAMA_baseline_total | Mental Health |
| 4 | Depressed Mood | HAMD01_baseline | Mental Health |
| 5 | Guilt | HAMD02_baseline | Mental Health |
| 6 | Suicide | HAMD03_baseline | Mental Health |
| 7 | Early Insomnia | HAMD04_baseline | Mental Health |
| 8 | Middle Insomnia | HAMD05_baseline | Mental Health |
| 9 | Late Insomnia | HAMD06_baseline | Mental Health |
| 10 | Work and Interests | HAMD07_baseline | Mental Health |
| 11 | Retardation | HAMD08_baseline | Mental Health |
| 12 | Agitation | HAMD09_baseline | Mental Health |
| 13 | Anxiety (Psychic) | HAMD10_baseline | Mental Health |
| 14 | Anxiety (Somatic) | HAMD11_baseline | Mental Health |
| 15 | Somatic Symptoms (Gastrointestinal) | HAMD12_baseline | Mental Health |
| 16 | Somatic Symptoms (General) | HAMD13_baseline | Mental Health |
| 17 | Genital Symptoms | HAMD14_baseline | Mental Health |
| 18 | Hypochondriasis | HAMD15_baseline | Mental Health |
| 19 | Weight Loss | HAMD16_baseline | Physical |
| 20 | Insight | HAMD17_baseline | Mental Health |

Supplementary Table 4.6 Phenotypic measures in the ADNI dataset.

|  | Description | ADNI field | Category |
| --- | --- | --- | --- |
| 1 | Age | age | Physical |
| 2 | Body Mass Index | body_mass_index | Physical |
| 3 | Delayed Logical Memory | Logical_memory_delayed | Cognition |
| 4 | Mini-Mental State Examination | MMSE | Cognition |
| 5 | Beta Amyloid level (Default A network) | amyloid_defaultA | PET |
| 6 | Beta Amyloid level (Default B network) | amyloid_defaultB | PET |

### Supplementary Tables 5.1-5.3. Distribution details for each dataset.

Supplementary Table 5.1 Demographics of each dataset.

|  | N | Age (Years) | Sex | Racial Groups |
| --- | --- | --- | --- | --- |
| HCP | 792 | 22 - 36 (mean = 28.6) | 371M / 421F | White (N = 612),  Black / African Am. (N = 94),  Am. Indian / Alaskan Nat. (N = 1),  Asian / Nat. Hawaiian / Other Pacific Is. (N = 53),  More than one (N = 21),  Unknown or Not Reported (N = 11) |
| ABCD-rest | 2565 | 9.00 - 10.9 (mean = 10.0) | 1251M / 1314F | White (N = 1443), Black (N = 277), Hispanic (N = 501),  Asian (N = 66),  Other (N = 273),  Not declared (N = 5) |
| ABCD-task | 2262 | 9.00 - 10.91 (mean = 10.01) | 1030M / 1232F | White (N = 1335), Black (N = 201), Hispanic (N = 425),  Asian (N = 60),  Other (N = 236),  Not declared (N = 5) |
| SINGER | 642 | 60 - 80 (mean = 68.8) | 309M / 333F | Chinese (N = 625), Malay (N = 2), Indian (N = 12), Other (N = 3) |
| TCP | 194 | 18.08 – 65.0 (mean = 33.4) | 81M / 110F / 3 SD | White (N = 116), Black or African American (N = 30), Asian (N = 29), More than one (N = 13), Other (N = 6) |
| MDD | 287 | 16 – 64 (mean = 32.3) | 101M / 186F | Chinese (N = 287) |
| ADNI | 586 | 50.8 – 97.5 (mean = 74.4) | 278M / 308F | White (N = 510), Black (N = 43), Asian (N = 15), American Indian (N = 1), More than one (N = 12), Unknown (N = 5) |

Supplementary Table 5.2 Diagnostic distributions for each dataset

|  | Diagnostic distributions |
| --- | --- |
| HCP | Healthy Controls |
| ABCD | Healthy Controls |
| SINGER | Elderly at risk for vascular cognitive impairment |
| TCP | Control (N = 76), MDD (N = 31), PTSD (N = 14), GAD (N = 12), Dysthymia (N = 9), Social Anxiety Disorder (N = 8), SUD (N = 8), BPD I (N = 6), BPD II (N = 6), Other Anxiety Disorder (N = 5), Other Mood Disorder (N = 4), Schizophrenia (N = 4), Schizoaffective Disorder (N = 4), ADHD (N = 3), Eating Disorder (N = 2), OCD (N = 2) |
| MDD | Major Depressive Disorder (N = 287) |
| ADNI | Control (N = 334), Mild Cognitive Impairment (N = 184), AD dementia (N = 68) |

Supplementary Table 5.3 Acquisition information for each dataset

|  | Acquisition | Voxel size/mm^3^ | TR/s | Atlas Space |
| --- | --- | --- | --- | --- |
| HCP | Single-echo Multi-band Custom Skyra | 2.0 | 0.72 | fsLR |
| ABCD | Single-echo Multi-band on GE & Siemens scanners (more information in Table S6.1) | 2.4 | 0.8 | fsaverage |
| SINGER | Multi-echo multi-band Prisma Fit | 3.0 | 1.0 | fsaverage |
| TCP | Single-echo multi-band on two Prisma scanners | 2.0 | 0.8 | MNI152 |
| MDD | Single-echo single-band on five Prisma scanners | 3.0 | 3.0 | fsaverage |
| ADNI | Single-echo multi-band (N = 78) & single-echo single-band (N = 508) on GE, Philips & Siemens scanners (more information in Table S6.2) | 2.5 & ~3.4 | 0.6 & 3 | fsaverage |

Supplementary Tables 6.1-6.3. Site clusters used for each dataset.

Supplementary Table 6.1. Site clusters for ABCD resting-state fMRI.

| ABCD Site | Make | Model | N | Site-cluster |
| --- | --- | --- | --- | --- |
| 2 | Siemens | Prisma fit | 97 | A |
| 6 | Siemens | Prisma fit | 67 | A |
| 12 | Siemens | Prisma fit | 79 | A |
| 4 | GE | Discovery MR750 | 315 | B |
| 3 | Siemens | Prisma | 183 | C |
| 9 | Siemens | Prisma fit | 51 | C |
| 5 | Siemens | Prisma fit | 97 | D |
| 20 | Siemens | Prisma/Prisma fit | 139 | D |
| 10 | GE | Discovery MR750 | 218 | E |
| 22 | GE | Discovery MR750 | 14 | E |
| 7 | Siemens | Prisma fit | 90 | F |
| 14 | Siemens | Prisma/Prisma fit | 150 | F |
| 13 | GE | Discovery MR750 | 245 | G |
| 11 | Siemens | Prisma | 100 | H |
| 15 | Siemens | Prisma fit | 69 | H |
| 21 | Siemens | Prisma fit/Prisma | 97 | H |
| 16 | Siemens | Prisma | 327 | I |
| 8 | GE | Discovery MR750 | 106 | J |
| 18 | GE | Discovery MR750 | 121 | J |

Supplementary Table 6.2 Site clusters for ADNI

| ADNI site | Make | Model | N | Site-cluster |
| --- | --- | --- | --- | --- |
| 58 | Siemens | Prisma fit | 33 | A |
| 2 | Siemens | Prisma | 27 | B |
| 23 | Siemens | Prisma fit | 4 | B |
| 28 | Siemens | Prisma fit | 25 | C |
| 3 | GE | Discovery MR750 | 4 | C |
| 33 | Siemens | Prisma fit | 15 | D |
| 55 | Siemens | Verio | 14 | D |
| 59 | Siemens | Prisma fit | 18 | E |
| 39 | GE | Signa premier | 11 | E |
| 18 | Siemens | Prisma fit | 10 | F |
| 16 | GE | Discovery MR750 | 19 | F |
| 20 | GE | Discovery MR750 | 22 | G |
| 52 | GE | Signa premier | 7 | G |
| 52 | GE | Discovery MR750w | 15 | H |
| 8 | Siemens | Prisma fit | 12 | H |
| 7 | Philips | Achieva | 1 | H |
| 9 | Siemens | Prisma | 1 | H |
| 50 | Philips | Achieva | 20 | I |
| 46 | GE | Discovery MR750 | 9 | I |
| 47 | GE | Discovery MR750 | 23 | J |
| 1 | Philips | Intera | 5 | J |
| 10 | Siemens | Biograph mMR | 1 | J |
| 25 | Siemens | Prisma fit | 18 | K |
| 40 | GE | Discovery MR750 | 11 | K |
| 4 | Philips | Ingenia | 23 | L |
| 13 | Philips | Achieva | 3 | L |
| 16 | GE | Signa UHP | 3 | L |
| 27 | Siemens | Prisma | 22 | M |
| 9 | Philips | Achieva | 7 | M |
| 49 | GE | Discovery MR750 | 17 | N |
| 1 | Siemens | Prisma fit | 11 | N |
| 21 | Philips | Achieva | 1 | N |
| 11 | Siemens | Verio | 9 | O |
| 15 | Siemens | Prisma fit | 9 | O |
| 21 | GE | Discovery MR750w | 9 | O |
| 22 | Philips | Achieva | 2 | O |
| 38 | Siemens | Prisma | 11 | P |
| 62 | Philips | Achieva | 8 | P |
| 14 | Philips | Achieva | 8 | P |
| 17 | Siemens | Prisma fit | 2 | P |
| 40 | Philips | Achieva | 5 | Q |
| 41 | Philips | Achieva | 5 | Q |
| 43 | Siemens | Skyra fit | 8 | Q |
| 26 | Siemens | Skyra | 9 | Q |
| 29 | Siemens | Prisma fit | 2 | Q |
| 43 | Siemens | Verio | 8 | R |
| 44 | Siemens | TrioTim | 5 | R |
| 45 | Philips | Ingenia | 4 | R |
| 5 | Siemens | Prisma | 5 | R |
| 60 | Philips | Ingenia | 2 | R |
| 61 | Philips | Ingenia | 5 | R |
| 13 | Philips | Achieva | 5 | S |
| 19 | Siemens | Prisma fit | 5 | S |
| 30 | Philips | Intera | 5 | S |
| 5 | Philips | Singa HDxt | 5 | S |
| 50 | Philips | Achieva | 6 | S |
| 63 | Siemens | Prisma | 3 | S |
| 17 | Siemens | TrioTim | 2 | T |
| 30 | Siemens | Prisma | 3 | T |
| 31 | Philips | Ingenia Elition X | 1 | T |
| 31 | Philips | Intera | 1 | T |
| 32 | GE | Discovery MR750 | 2 | T |
| 34 | Siemens | TrioTim | 1 | T |
| 37 | Philips | Singa HDxt | 3 | T |
| 37 | Siemens | Verio | 3 | T |
| 39 | GE | Discovery MR750 | 1 | T |
| 41 | Philips | Ingenia Elition X | 1 | T |
| 51 | Philips | Ingenia | 2 | T |
| 59 | Philips | Achieva | 3 | T |
| 6 | GE | Discovery MR750w | 4 | T |
| 7 | GE | Signa premier | 2 | T |

Supplementary Table 6.3 Site clusters for ABCD task-fMRI

| ABCD Site | Make | Model | N | Site-cluster |
| --- | --- | --- | --- | --- |
| 2 | Siemens | Prisma fit | 129 | A |
| 6 | Siemens | Prisma fit | 150 | A |
| 12 | Siemens | Prisma fit | 116 | A |
| 4 | GE | Discovery MR750 | 170 | B |
| 3 | GE | Discovery MR750 | 160 | C |
| 9 | Siemens | Prisma | 66 | C |
| 5 | Siemens | Prisma fit | 72 | D |
| 20 | Siemens | Prisma fit | 103 | D |
| 10 | Siemens | Prisma/Prisma fit | 161 | E |
| 22 | GE | Discovery MR750 | 13 | E |
| 7 | Siemens | Prisma fit | 71 | F |
| 14 | Siemens | Prisma/Prisma fit | 165 | F |
| 13 | GE | Discovery MR750 | 192 | G |
| 11 | Siemens | Prisma | 71 | H |
| 15 | Siemens | Prisma fit | 37 | H |
| 12 | Siemens | Prisma fit/Prisma | 93 | H |
| 16 | Siemens | Prisma | 337 | I |
| 8 | GE | Discovery MR750 | 74 | J |
| 18 | GE | Discovery MR750 | 82 | J |

##
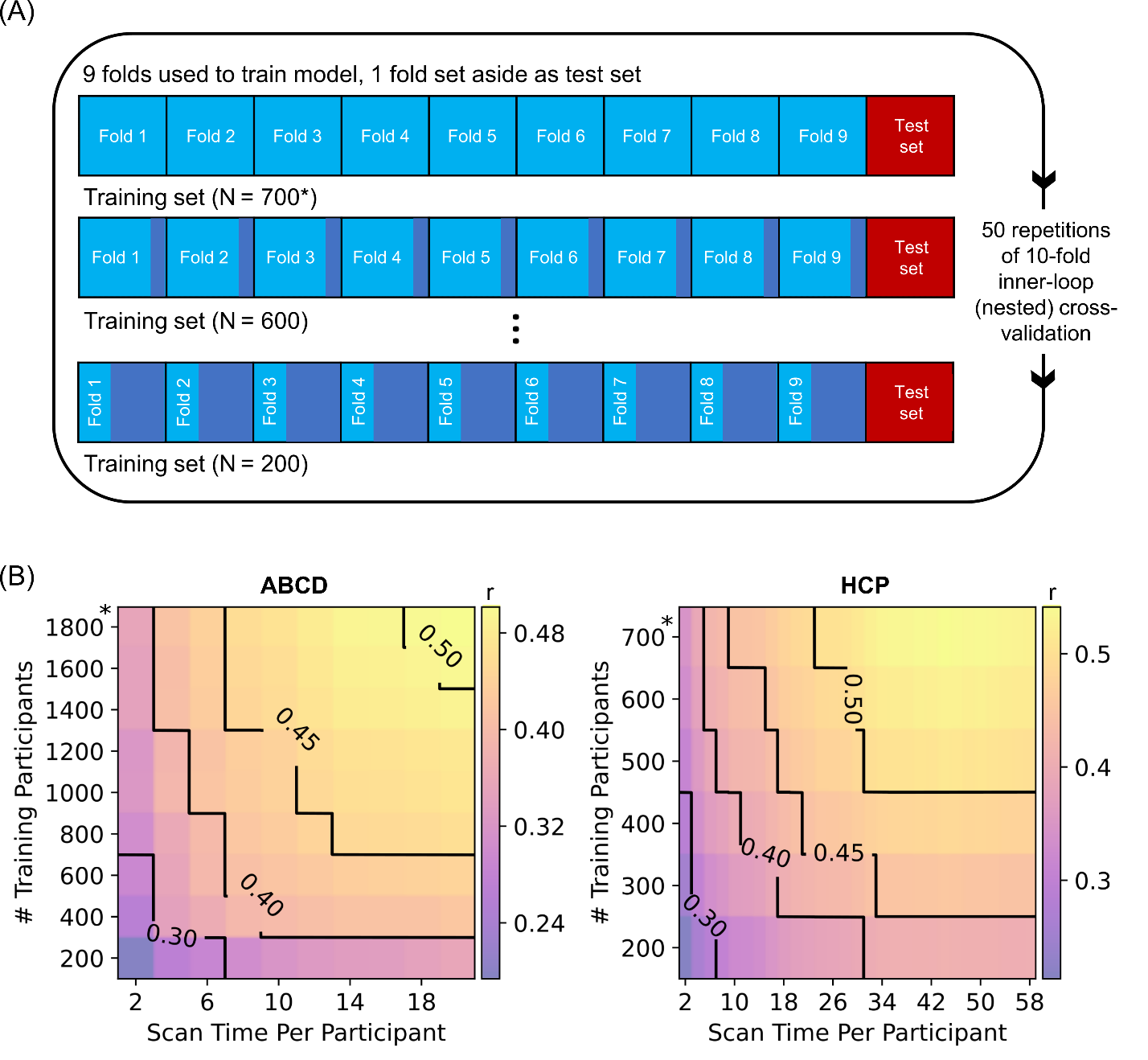
Supplementary Figures

### Supplementary Fig. 1 | Contour plot of HCP prediction accuracy.

Contour plot of prediction accuracy (Pearson’s correlation) of the cognitive factor score as a function of the scan time *T* used to generate the functional connectivity matrix, and the number of training participants *N* used to train the predictive model in the Human Connectome Project (HCP) dataset. Increasing training participants and scan time both improved prediction performance. The * indicates that all available participants were used, therefore the sample size will be close to, but not exactly the number shown.


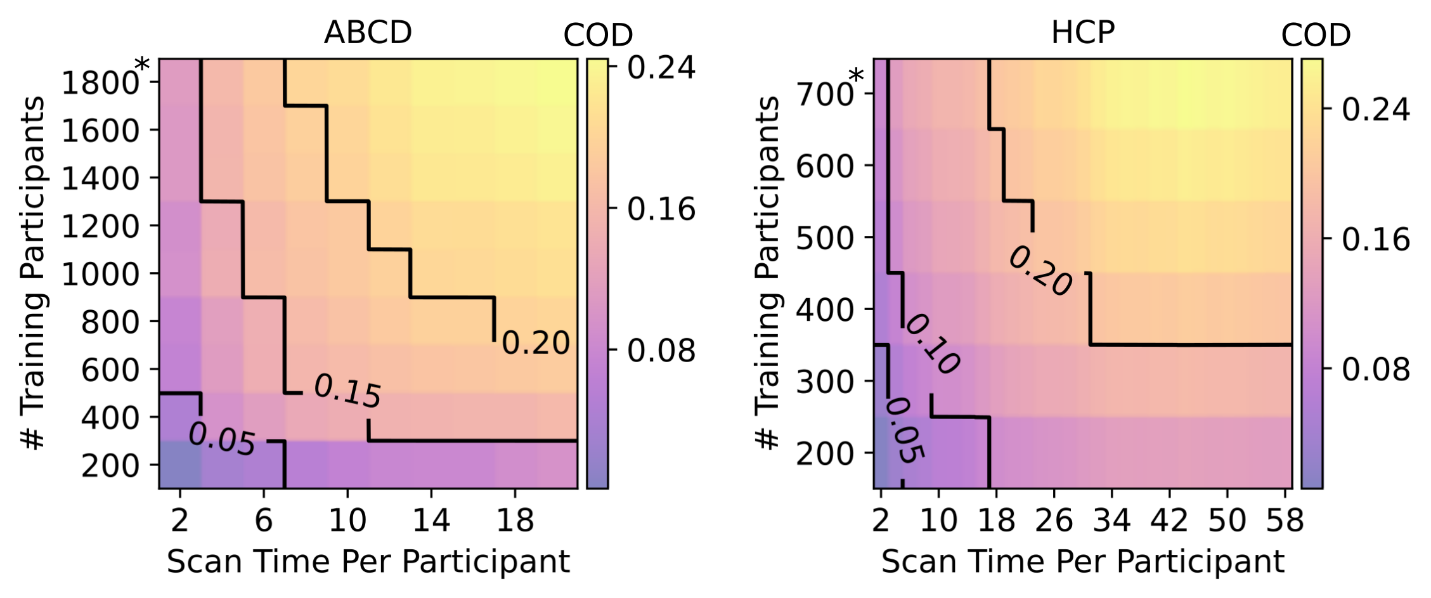


Supplementary Fig. 2 | Contour plot of ABCD & HCP prediction accuracy in terms of COD.

Same as Fig. 1a except prediction accuracy was calculated with Coefficient of Determination (COD or R^2^) instead of Pearson’s correlation. Contour plot of prediction accuracy (COD) of the cognitive component score as a function of the scan time used to generate the functional connectivity matrix (x-axis), and the number of training participants used to train the predictive model (y-axis) in the ABCD and HCP datasets. Increasing training participants and scan time both led to increases in prediction performance. The * in both figures indicates that all available participants were used, therefore the sample size will be close to, but not exactly the number shown.


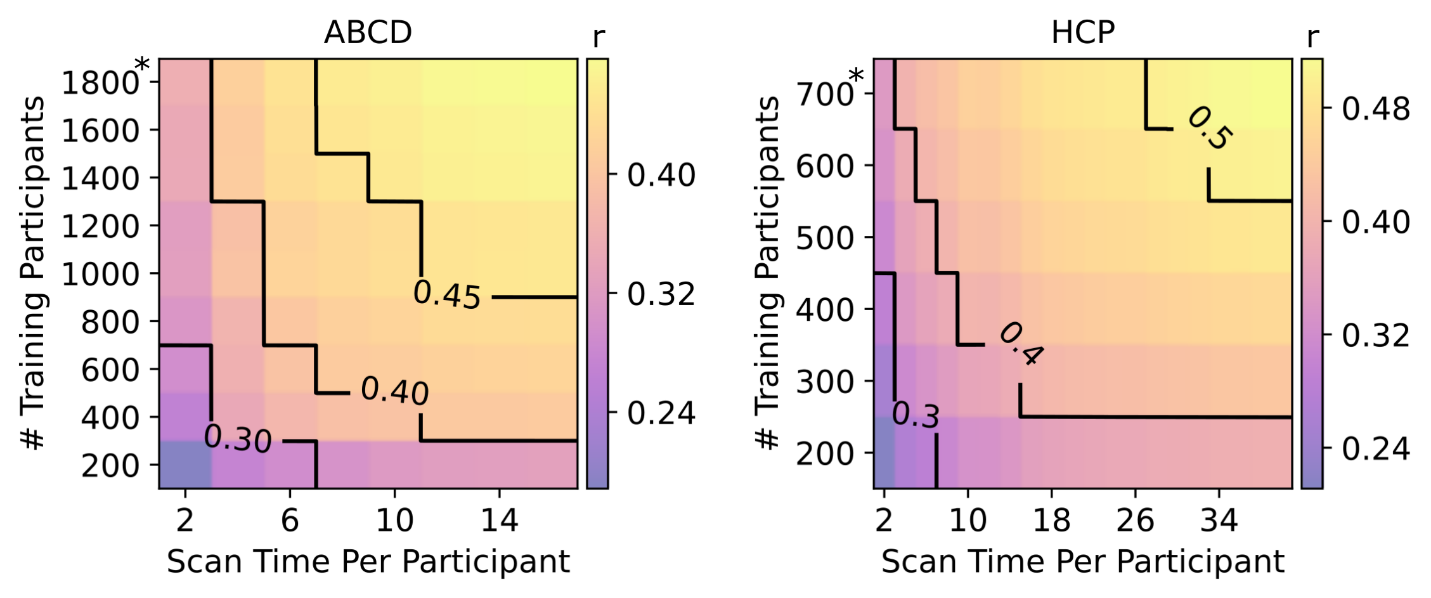


### Supplementary Fig. 3 | Contour plot of ABCD & HCP prediction after censoring.

Same as Fig. 1a except functional connectivity matrices constructed with first *T* minutes of uncensored data. Contour plot of prediction accuracy (Pearson’s correlation) of the cognitive component score as a function of the scan time used to generate the functional connectivity matrix (x-axis), and the number of training participants used to train the predictive model (y-axis) in the ABCD and HCP datasets. Increasing training participants and scan time both led to increases in prediction performance. The * in both figures indicates that all available participants were used, therefore the sample size will be close to, but not exactly the number shown.


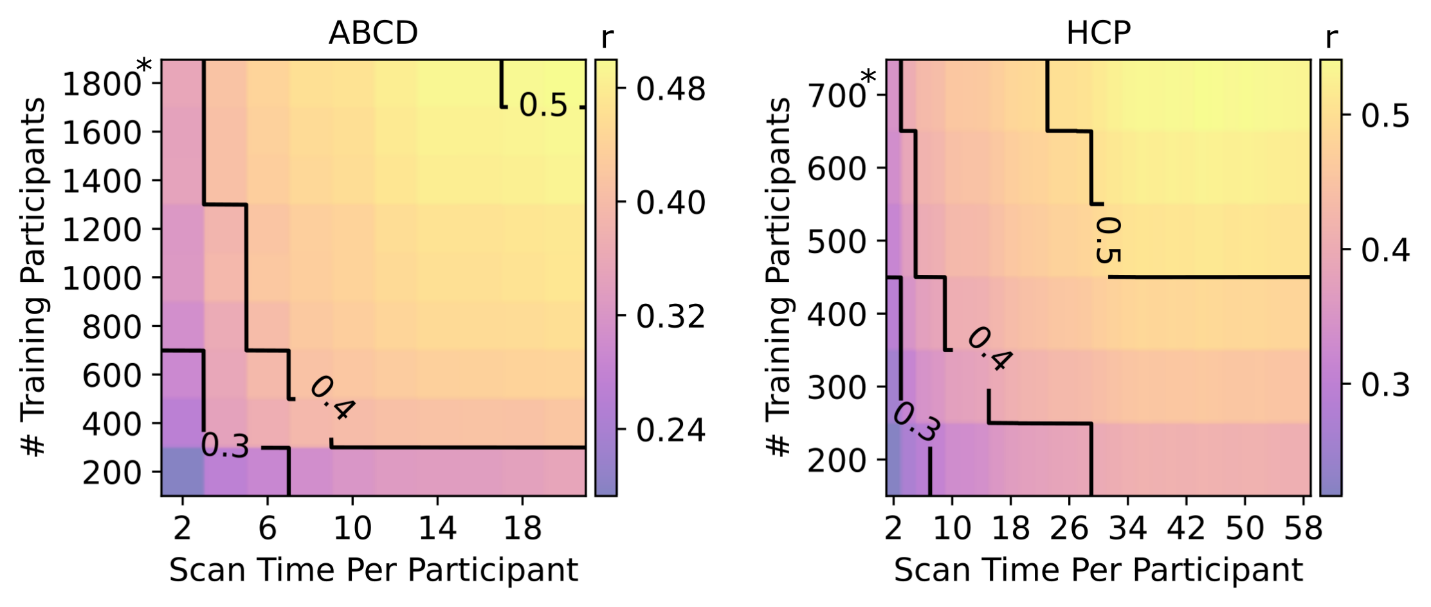


### Supplementary Fig. 4 | Contour plot of ABCD & HCP prediction accuracy using data without censoring.

Same as Fig. 1a except censored frames were not excluded when computing the functional connectivity matrices. Contour plot of prediction accuracy (Pearson’s correlation) of the cognitive component score as a function of the scan time used to generate the functional connectivity matrix (x-axis), and the number of training participants used to train the predictive model (y-axis) in the ABCD and HCP datasets. Increasing training participants and scan time both led to increases in prediction performance. The * in both figures indicates that all available participants were used, therefore the sample size will be close to, but not exactly the number shown.


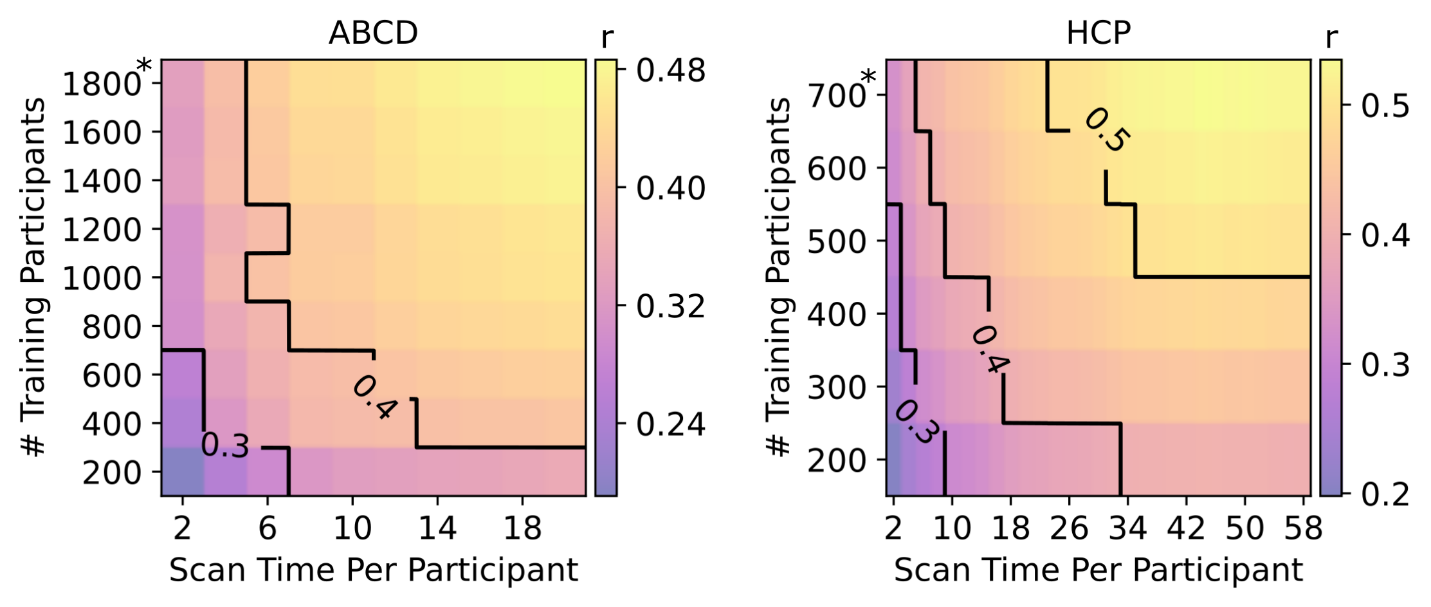


### Supplementary Fig. 5 | Contour plot of ABCD & HCP prediction accuracy (Pearson’s Correlation) using LRR.

Same as Fig. 1a except linear ridge regression was used as the prediction algorithm instead of kernel ridge regression. Contour plot of prediction accuracy (Pearson’s correlation) of the cognitive component score as a function of the scan time used to generate the functional connectivity matrix (x-axis), and the number of training participants used to train the predictive model (y-axis) in the ABCD and HCP datasets. Increasing training participants and scan time both led to increases in prediction performance. The * in both figures indicates that all available participants were used, therefore the sample size will be close to, but not exactly the number shown.


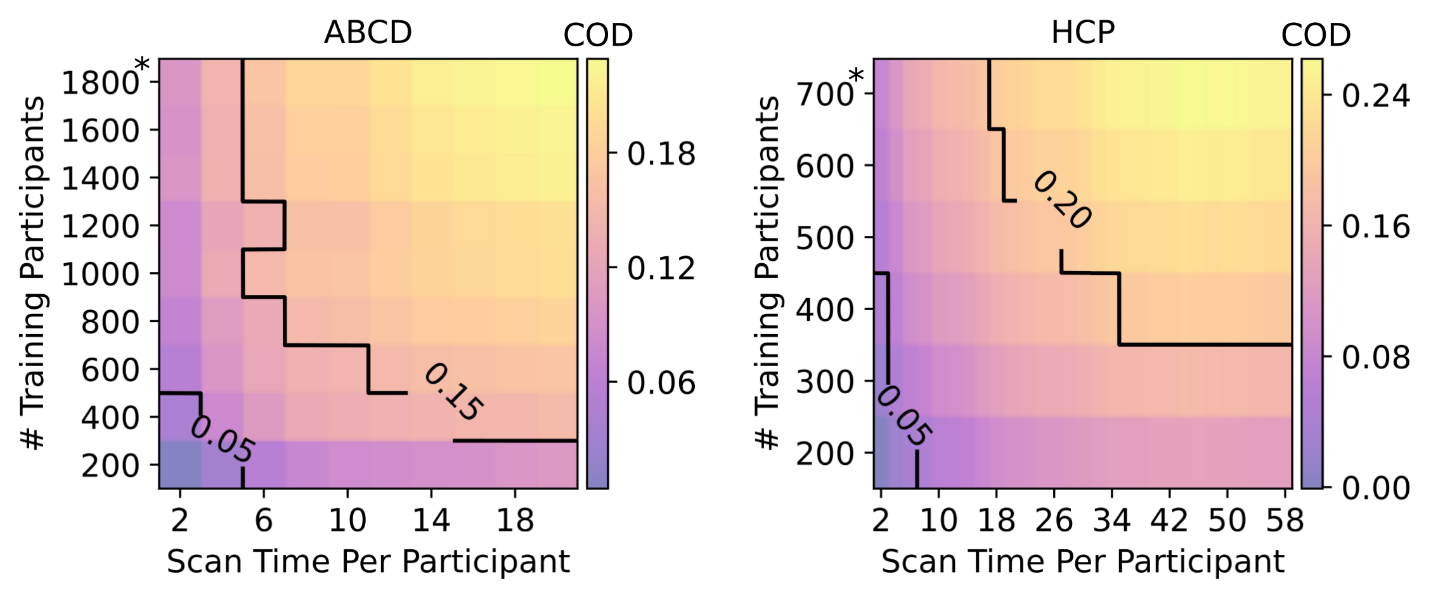


### Supplementary Fig. 6 | Contour plot of ABCD & HCP prediction accuracy (COD) using LRR.

Same as Fig. 1a except linear ridge regression was used as the prediction algorithm instead of kernel ridge regression and prediction accuracy was calculated with Coefficient of Determination (COD or R^2^) instead of Pearson’s correlation. Contour plot of prediction accuracy (COD) of the cognitive component score as a function of the scan time used to generate the functional connectivity matrix (x-axis), and the number of training participants used to train the predictive model (y-axis) in the ABCD and HCP datasets. Increasing training participants and scan time both led to increases in prediction performance. The * in both figures indicates that all available participants were used, therefore the sample size will be close to, but not exactly the number shown.

Supplementary Fig. 7.1-7.3 | Scatter plots for 17 phenotypic measures in the ABCD dataset.


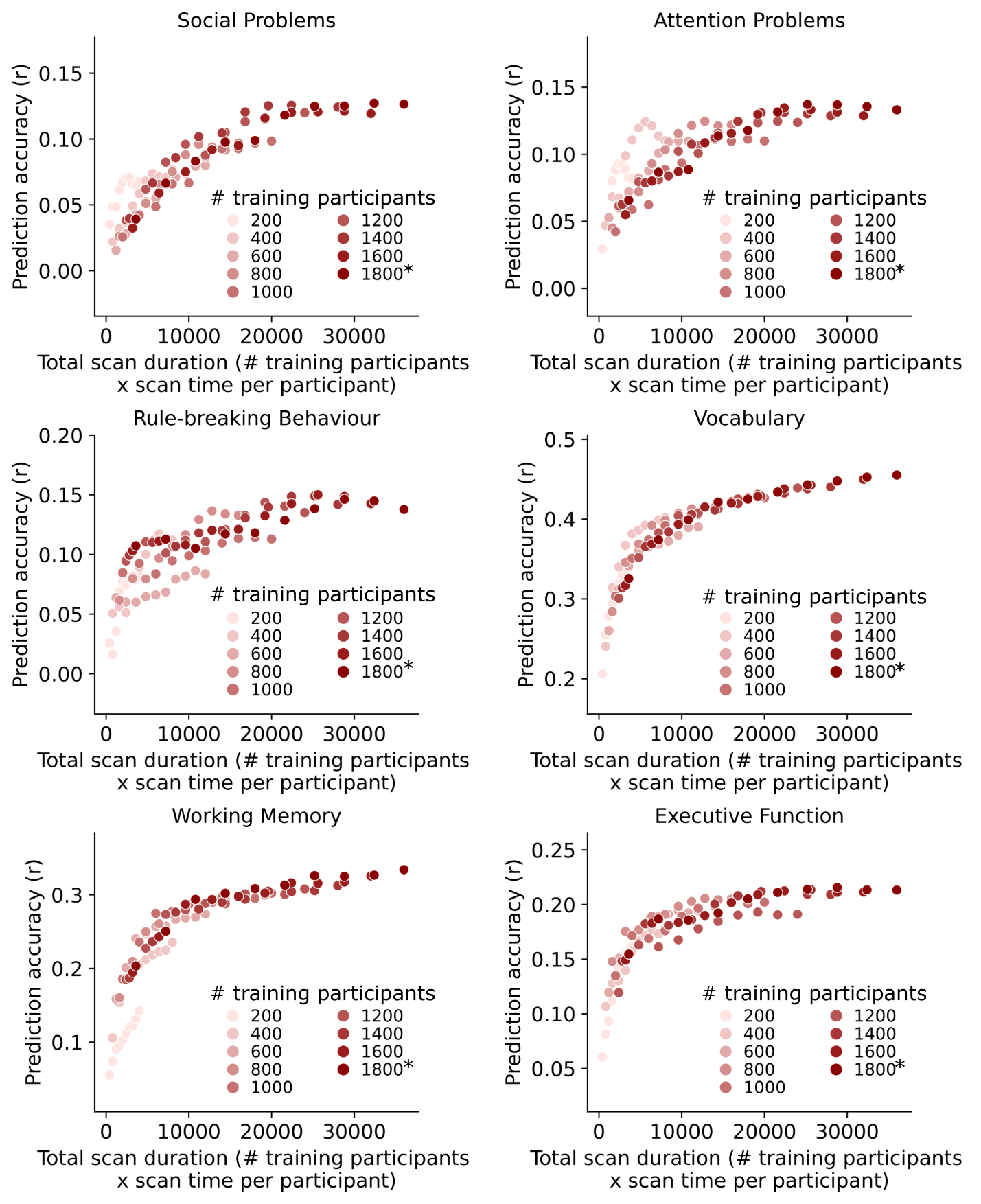


Supplementary Fig. 7.1 | Same as Fig. 2a except showing the scatter plots for 6 of the 17 phenotypic measures in the ABCD dataset that visually follow a logarithmic pattern. Scatter plots showing prediction accuracy (Pearson’s correlation) as a function of total scan duration (defined as # training participants x scan time per participant). The * in the figures indicates that all available participants were used, therefore the sample size will be close to, but not exactly the number shown.


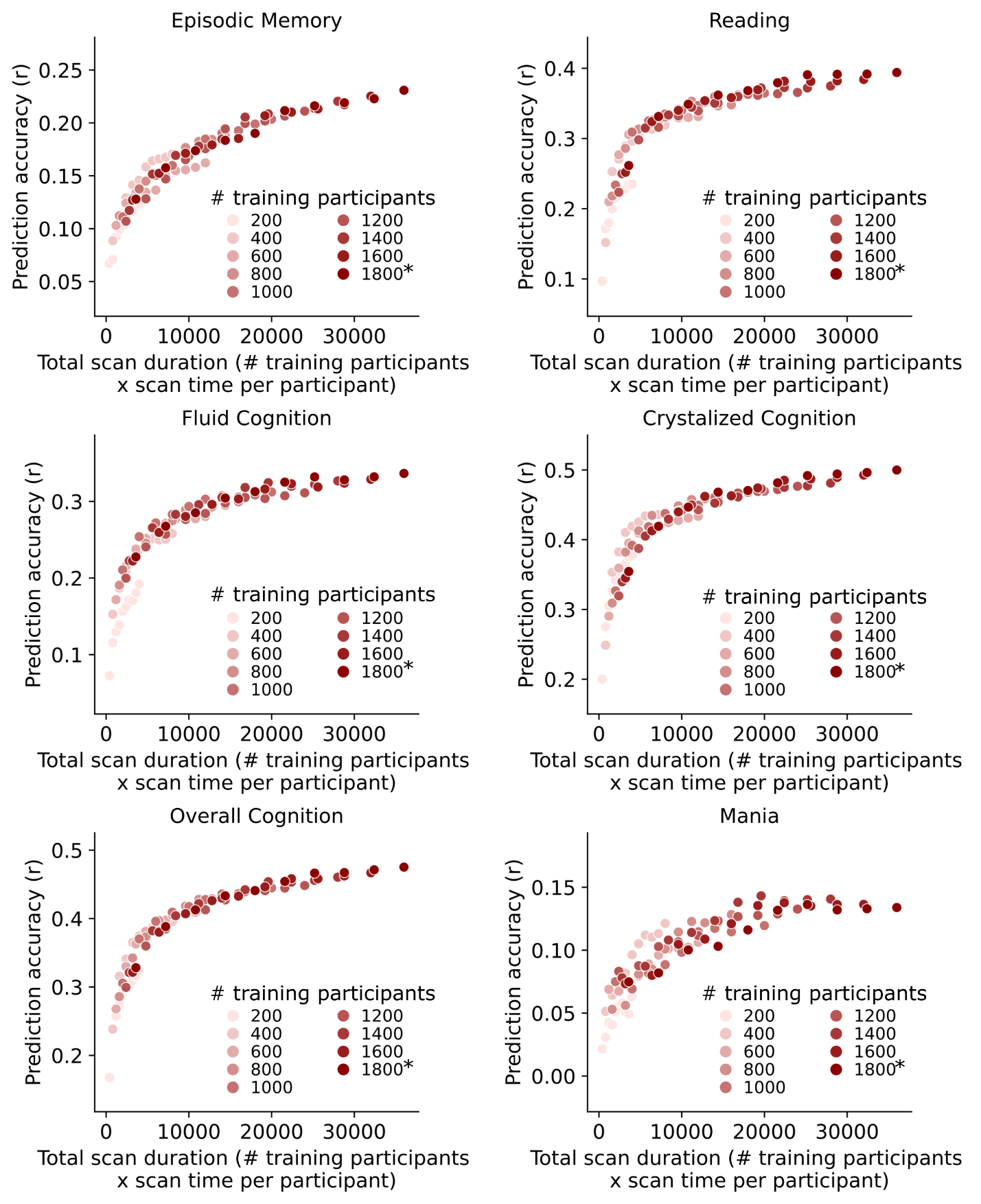


Supplementary Fig. 7.2 | Same as Fig. 2a except showing the scatter plots for 6 of the 17 phenotypic measures in the ABCD dataset that visually follow a logarithmic pattern. Scatter plots showing prediction accuracy (Pearson’s correlation) as a function of total scan duration (defined as # training participants x scan time per participant). The * in the figures indicates that all available participants were used, therefore the sample size will be close to, but not exactly the number shown.


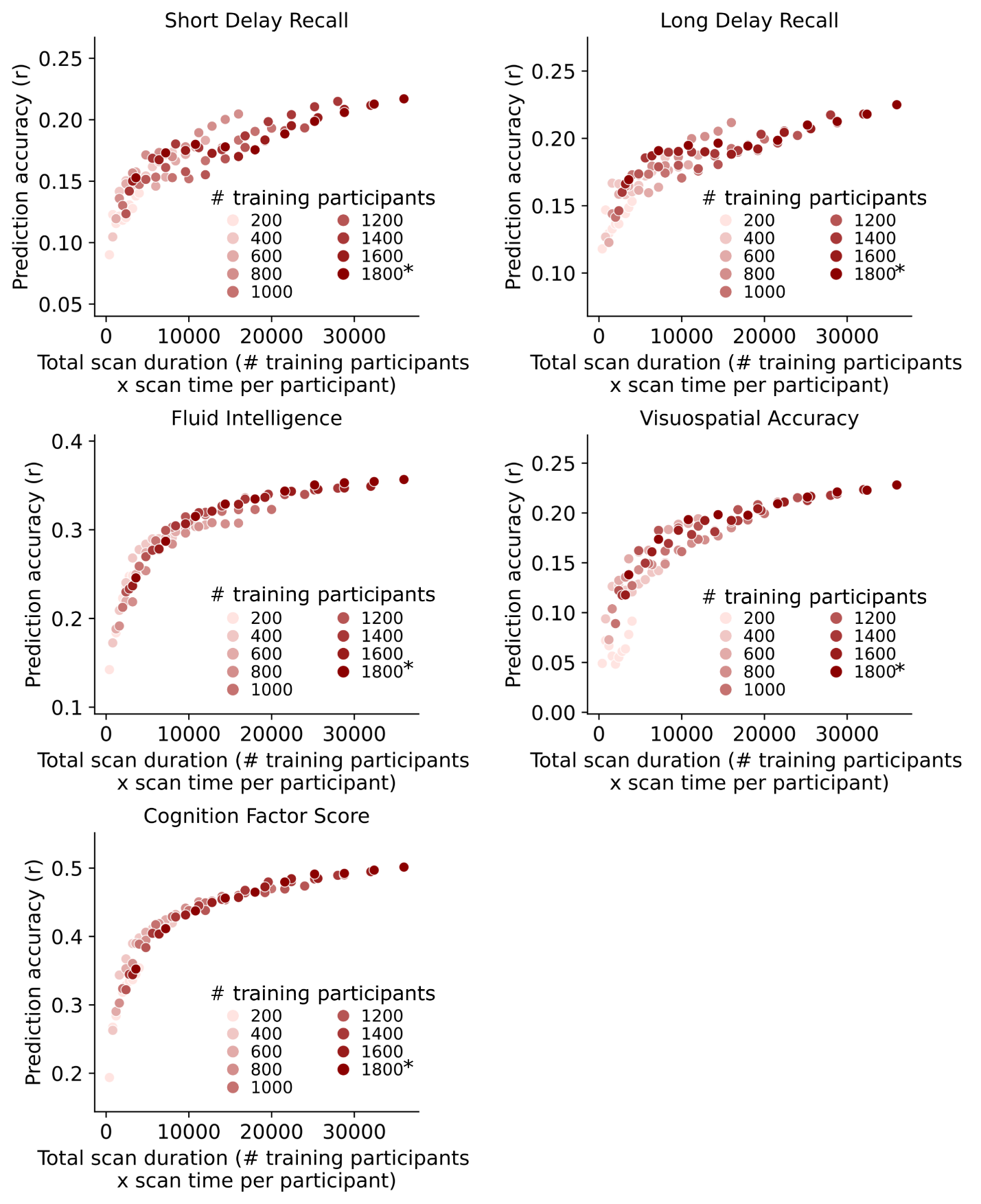


Supplementary Fig. 7.3 | Same as Fig. 2a except showing the scatter plots for 5 of the 17 phenotypic measures in the ABCD dataset that visually follow a logarithmic pattern. Scatter plots showing prediction accuracy (Pearson’s correlation) as a function of total scan duration (defined as # training participants x scan time per participant). The * in the figures indicates that all available participants were used, therefore the sample size will be close to, but not exactly the number shown.

Supplementary Fig. 8.1-8.7 | Scatter plots for 19 phenotypic measures in the HCP dataset.


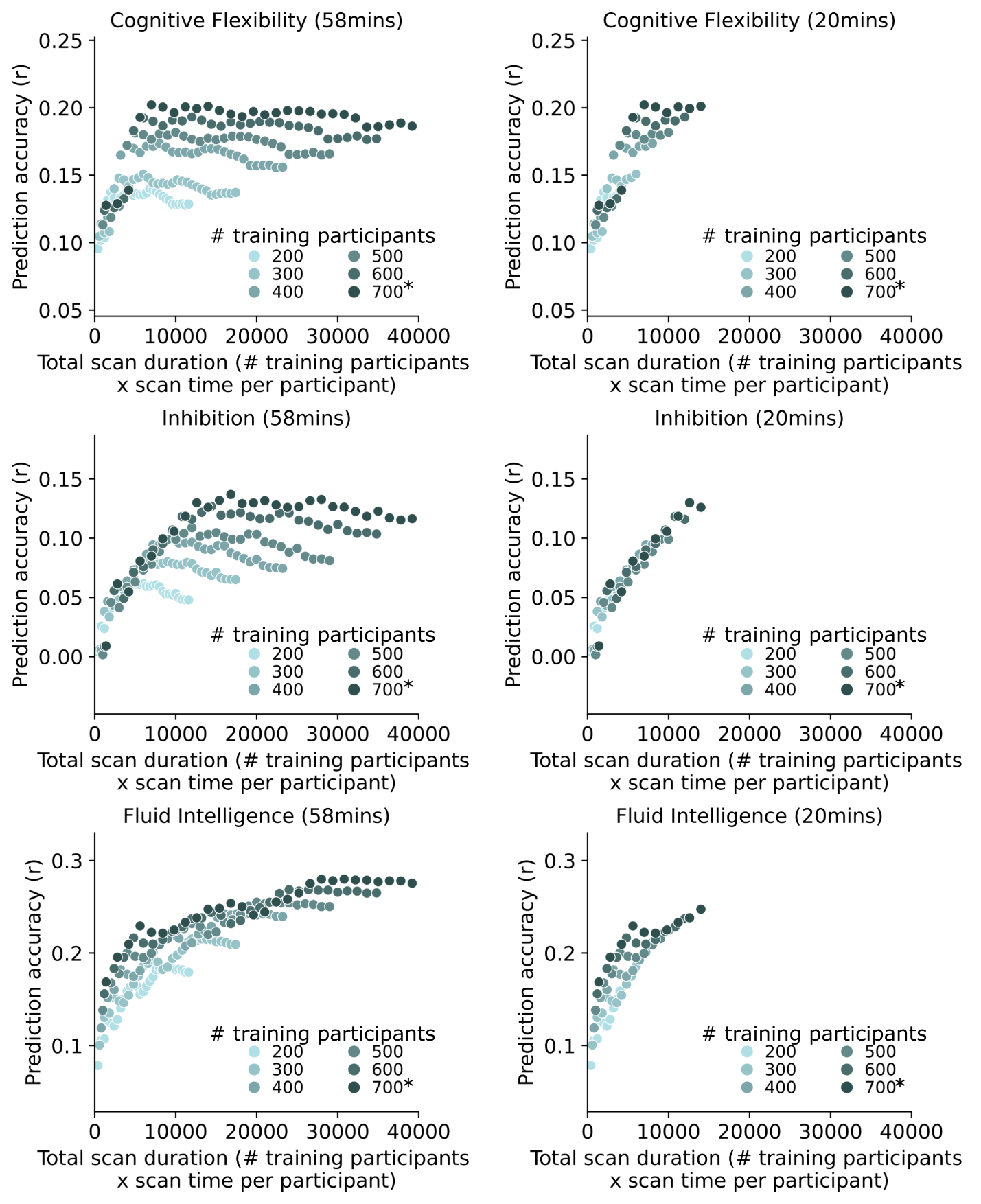


Supplementary Fig. 8.1 | Same as Fig. 2a except showing the scatter plots for 3 of the 19 phenotypic measures in the HCP dataset that visually follow a logarithmic pattern. Scatter plots showing prediction accuracy (Pearson’s correlation) as a function of total scan duration (defined as # training participants x scan time per participant). Scatter plots are shown for the full scan time (left panels) and up to 20 mins of scan time per participant (right panels). The * in the figures indicates that all available participants were used, therefore the sample size will be close to, but not exactly the number shown.


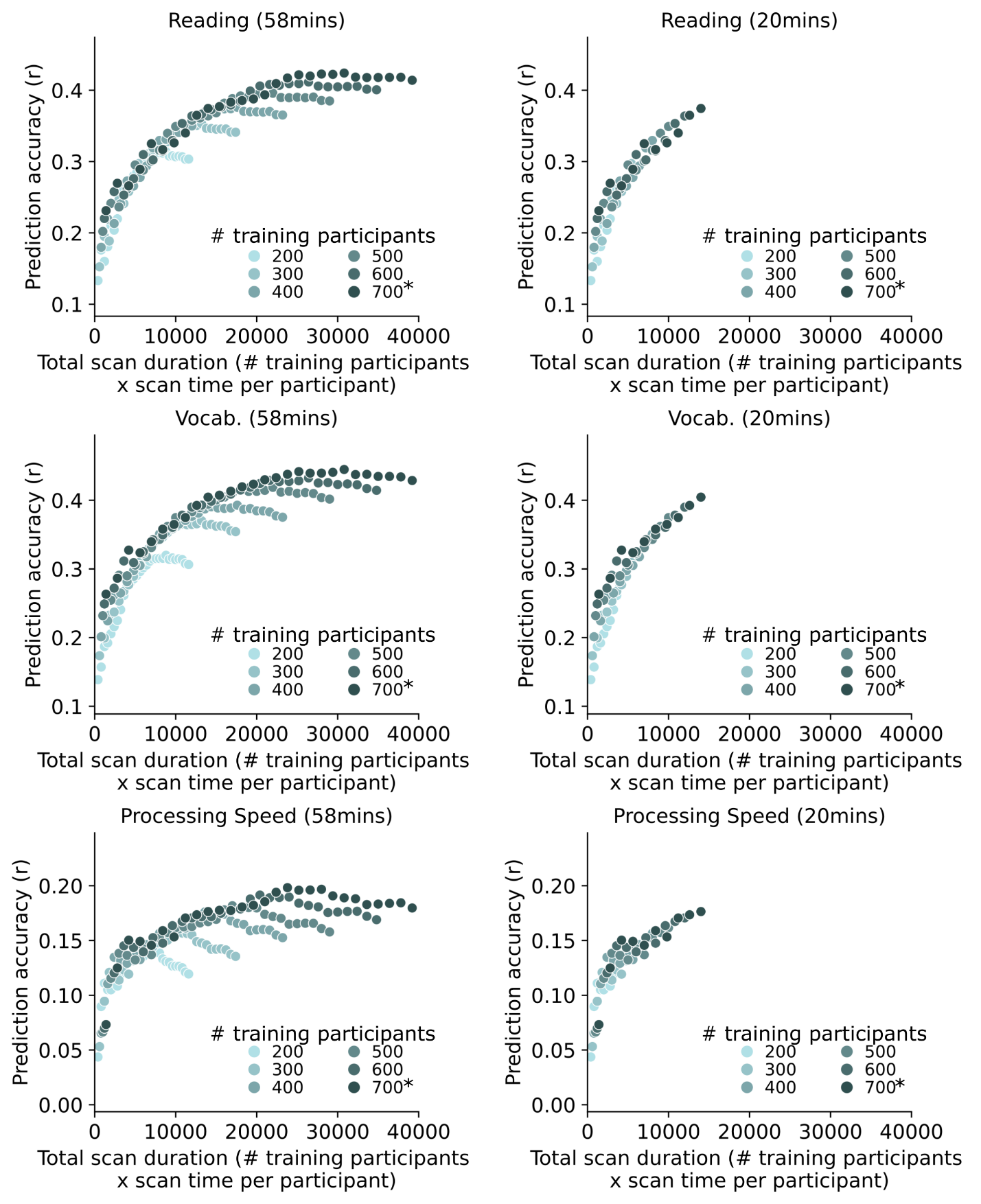


Supplementary Fig. 8.2 | Same as Fig. 2a except showing the scatter plots for 3 of the 19 phenotypic measures in the HCP dataset that visually follow a logarithmic pattern. Scatter plots showing prediction accuracy (Pearson’s correlation) as a function of total scan duration (defined as # training participants x scan time per participant). Scatter plots are shown for the full scan time (left panels) and up to 20 mins of scan time per participant (right panels). The * in the figures indicates that all available participants were used, therefore the sample size will be close to, but not exactly the number shown.


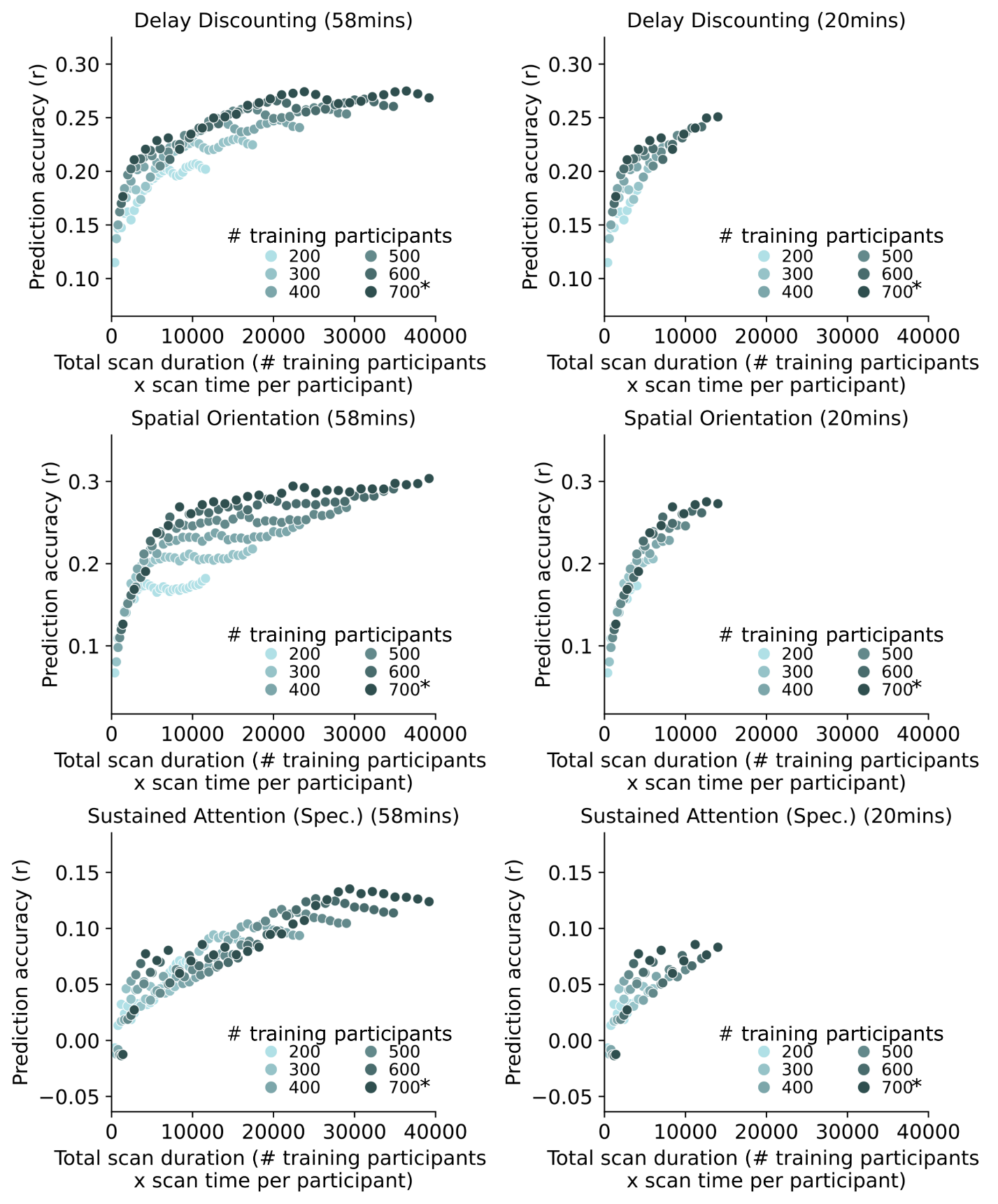


Supplementary Fig. 8.3 | Same as Fig. 2a except showing the scatter plots for 3 of the 19 phenotypic measures in the HCP dataset that visually follow a logarithmic pattern. Scatter plots showing prediction accuracy (Pearson’s correlation) as a function of total scan duration (defined as # training participants x scan time per participant). Scatter plots are shown for the full scan time (left panels) and up to 20 mins of scan time per participant (right panels). The * in the figures indicates that all available participants were used, therefore the sample size will be close to, but not exactly the number shown.


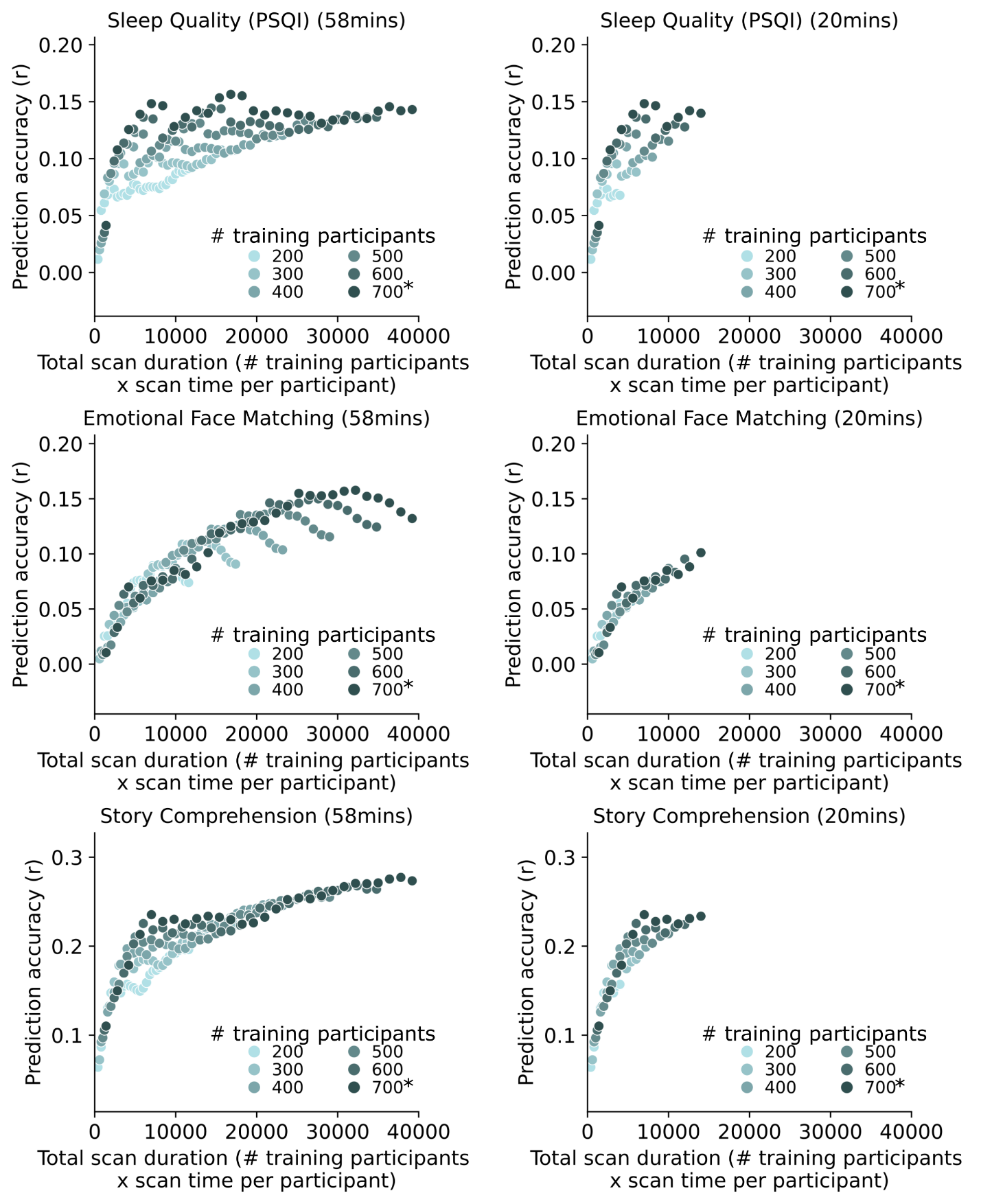


Supplementary Fig. 8.4 | Same as Fig. 2a except showing the scatter plots for 3 of the 19 phenotypic measures in the HCP dataset that visually follow a logarithmic pattern. Scatter plots showing prediction accuracy (Pearson’s correlation) as a function of total scan duration (defined as # training participants x scan time per participant). Scatter plots are shown for the full scan time (left panels) and up to 20 mins of scan time per participant (right panels). The * in the figures indicates that all available participants were used, therefore the sample size will be close to, but not exactly the number shown.


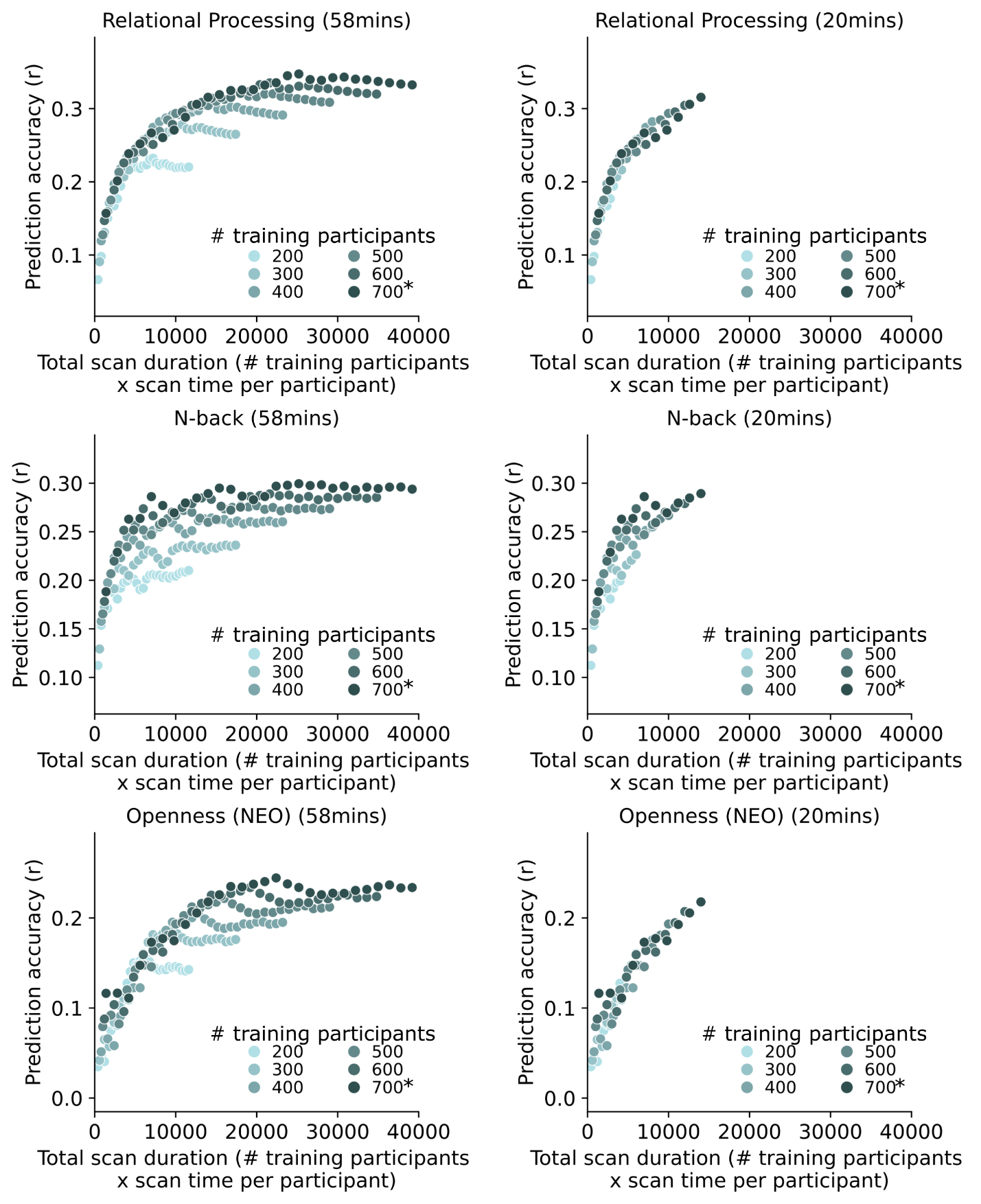


Supplementary Fig. 8.5 | Same as Fig. 2a except showing the scatter plots for 3 of the 19 phenotypic measures in the HCP dataset that visually follow a logarithmic pattern. Scatter plots showing prediction accuracy (Pearson’s correlation) as a function of total scan duration (defined as # training participants x scan time per participant). Scatter plots are shown for the full scan time (left panels) and up to 20 mins of scan time per participant (right panels). The * in the figures indicates that all available participants were used, therefore the sample size will be close to, but not exactly the number shown.


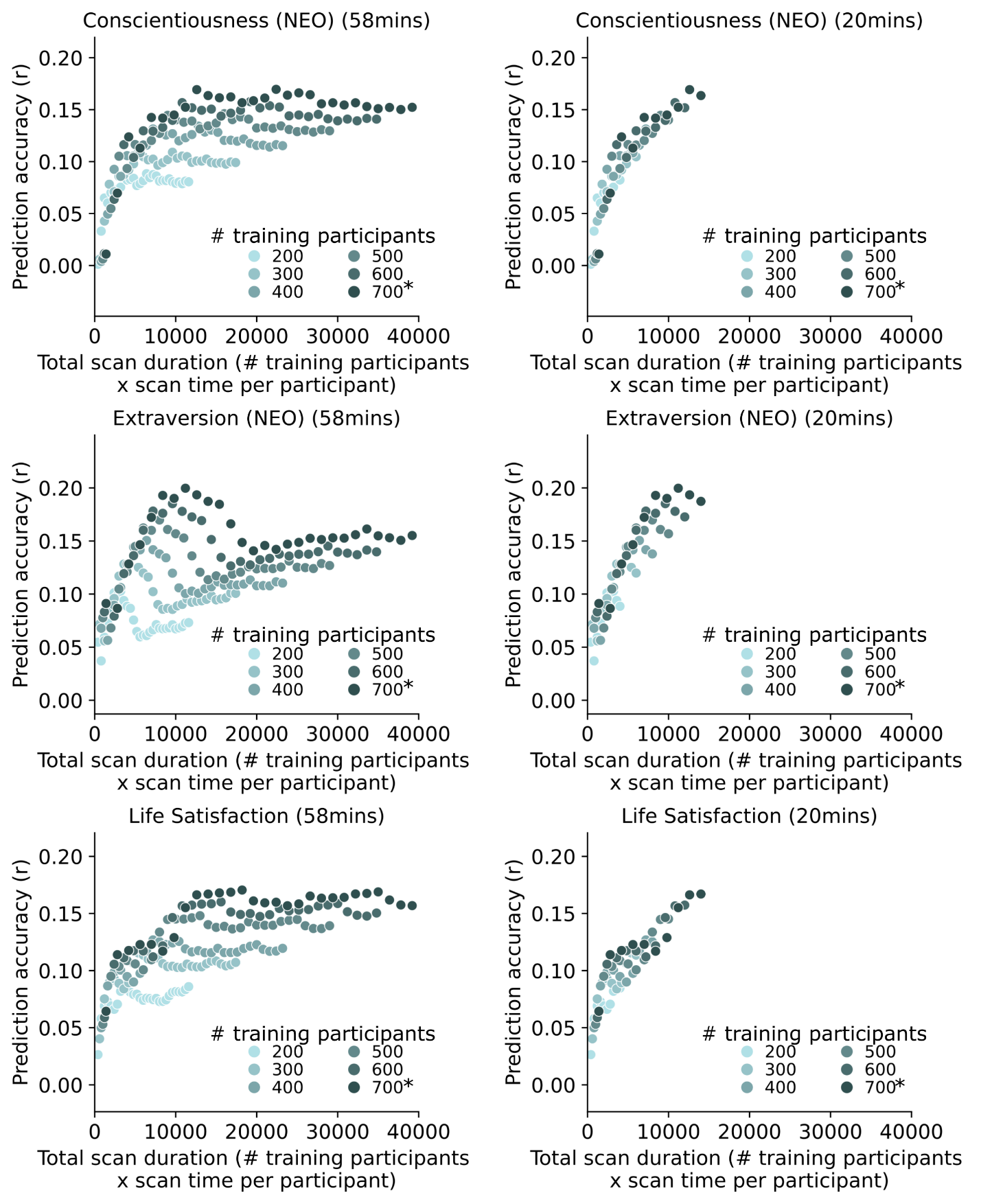


Supplementary Fig. 8.6 | Same as Fig. 2a except showing the scatter plots for 3 of the 19 phenotypic measures in the HCP dataset that visually follow a logarithmic pattern. Scatter plots showing prediction accuracy (Pearson’s correlation) as a function of total scan duration (defined as # training participants x scan time per participant). Scatter plots are shown for the full scan time (left panels) and up to 20 mins of scan time per participant (right panels). The * in the figures indicates that all available participants were used, therefore the sample size will be close to, but not exactly the number shown.


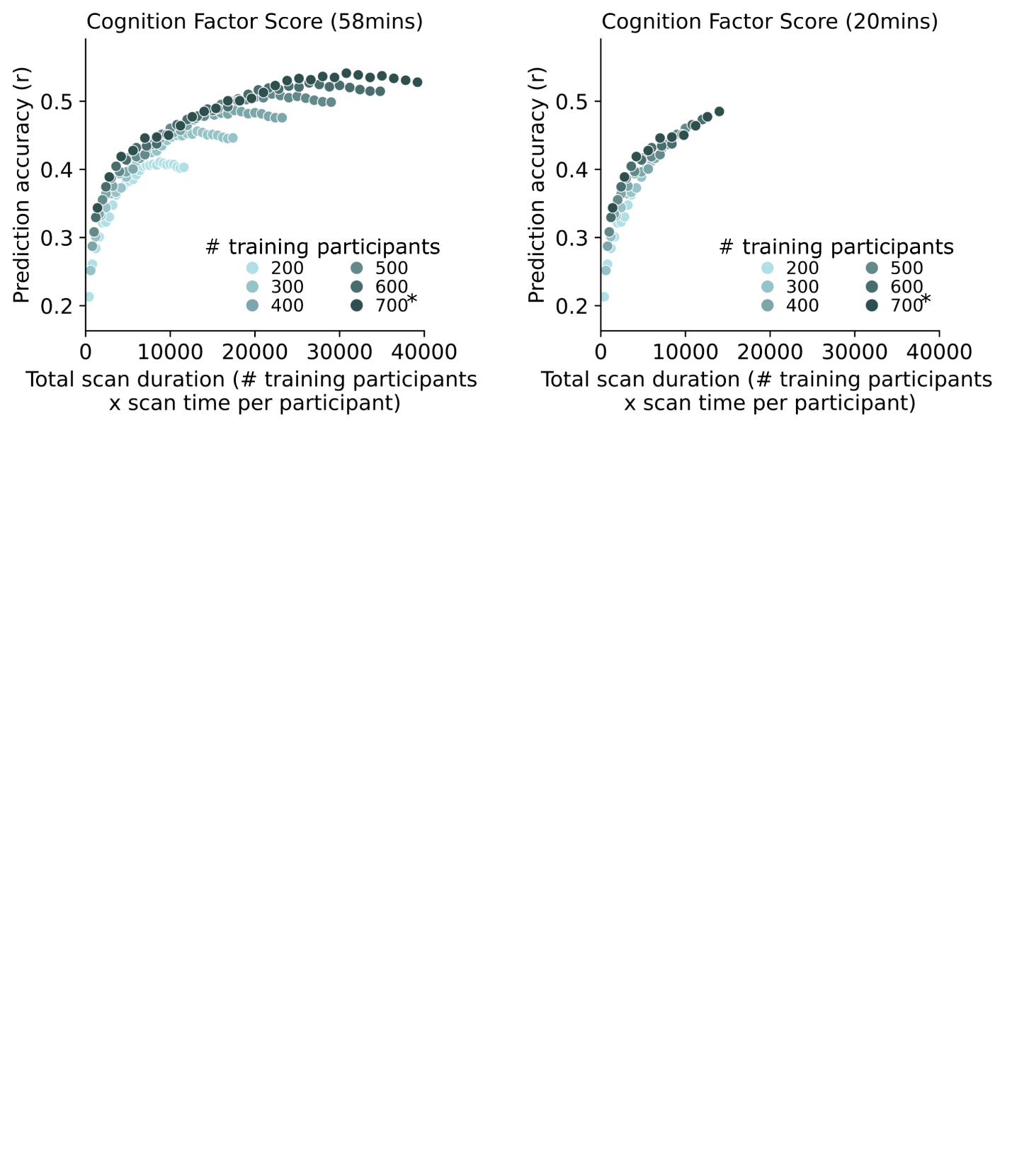


Supplementary Fig. 8.7 | Same as Fig. 2a except showing the scatter plots for 1 of the 19 phenotypic measures in the HCP dataset that visually follow a logarithmic pattern. Scatter plots showing prediction accuracy (Pearson’s correlation) as a function of total scan duration (defined as # training participants x scan time per participant). Scatter plots are shown for the full scan time (left panels) and up to 20 mins of scan time per participant (right panels). The * in the figures indicates that all available participants were used, therefore the sample size will be close to, but not exactly the number shown.


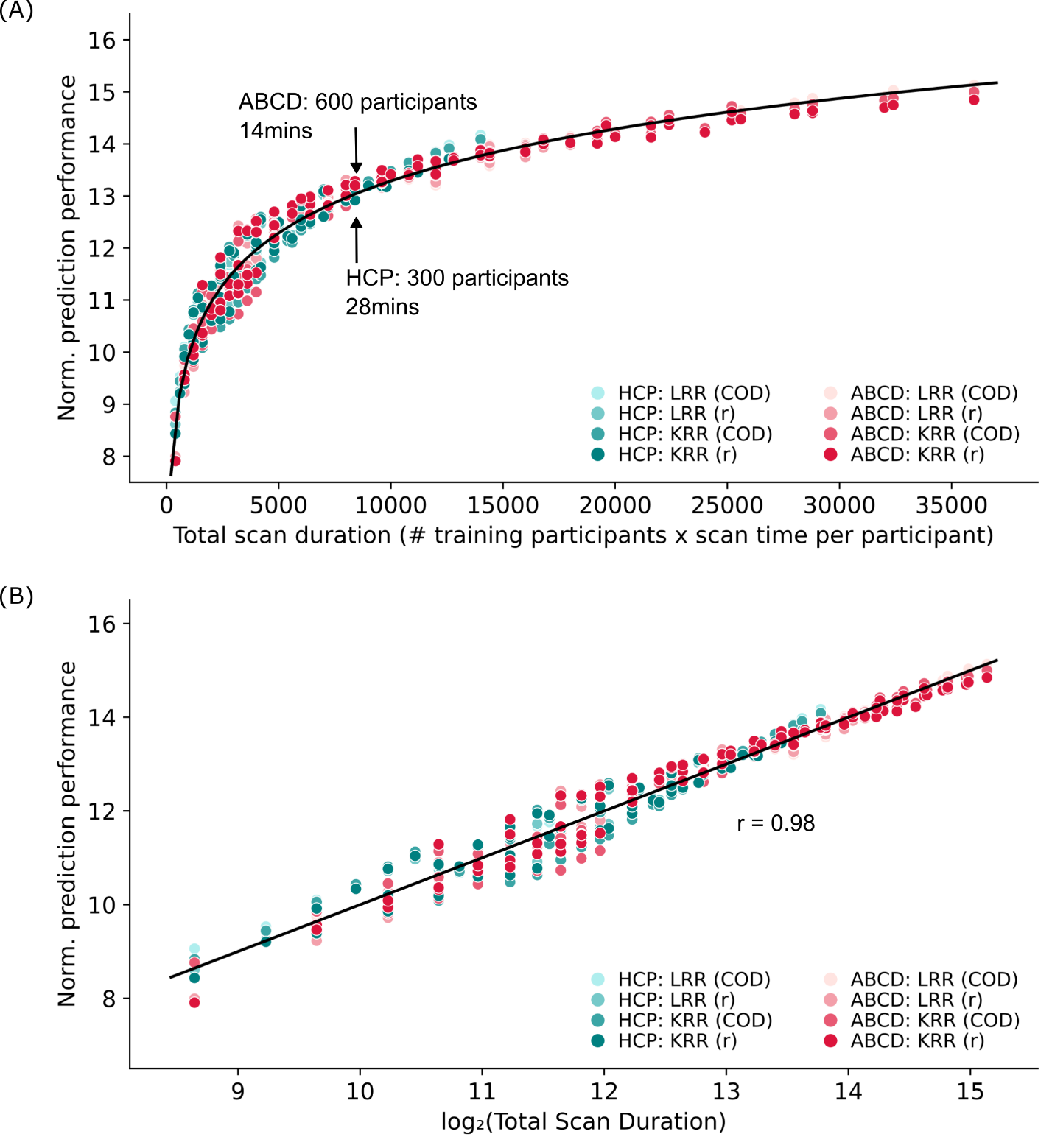


### Supplementary Fig. 9 | Consistent logarithmic relationship across algorithms and metrics

a. Same as Extended Data Fig. 2, except showing the relationship between total scan duration and prediction accuracy of the cognitive factor scores ignoring data beyond 20 min of scan time. Shown for different regressions (KRR, LRR) and different accuracy metrics (Pearson’s correlation and Coefficient of Determination). Black arrows show that scanning 300 participants for 28 minutes (total scan duration = 300 × 28 = 8400 minutes) in the HCP dataset, or 600 participants for 14 minutes (total scan duration = 600 × 14 = 8400 minutes) in the ABCD dataset yielded very similar normalized prediction accuracies b. Same as Fig. 2b, except showing the relationship between the logarithm of total scan duration and prediction accuracy of the cognitive factor scores versus total scan duration ignoring data beyond 30 min of scan time. Shown for different regressions (KRR, LRR) and different accuracy metrics (Pearson’s correlation and Coefficient of Determination).

Supplementary Fig. 10.1-10.3 | Theoretical model fit for 17 phenotypic measures in the ABCD dataset.


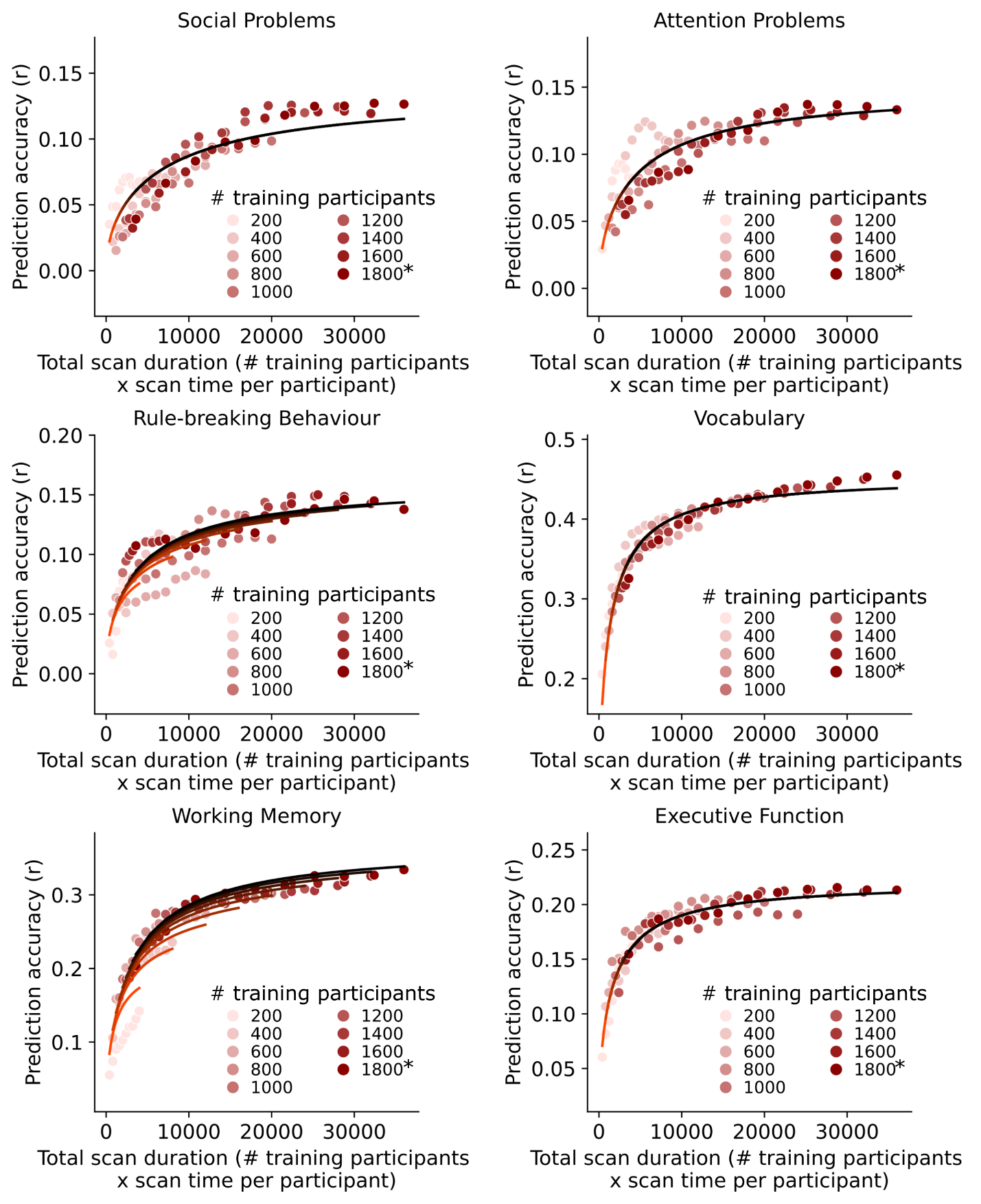
Supplementary Fig. 10.1 | Same as Fig. 3b except showing the scatter plots and the fit of the theoretical model for 6 of 17 phenotypic measures in the ABCD dataset that visually follow a logarithmic pattern. Scatter plot of prediction accuracy against total scan duration in the ABCD dataset. The curves were obtained by fitting the theoretical model to the prediction accuracies of the phenotype. The * in the figures indicates that all available participants were used, therefore the sample size will be close to, but not exactly the number shown.


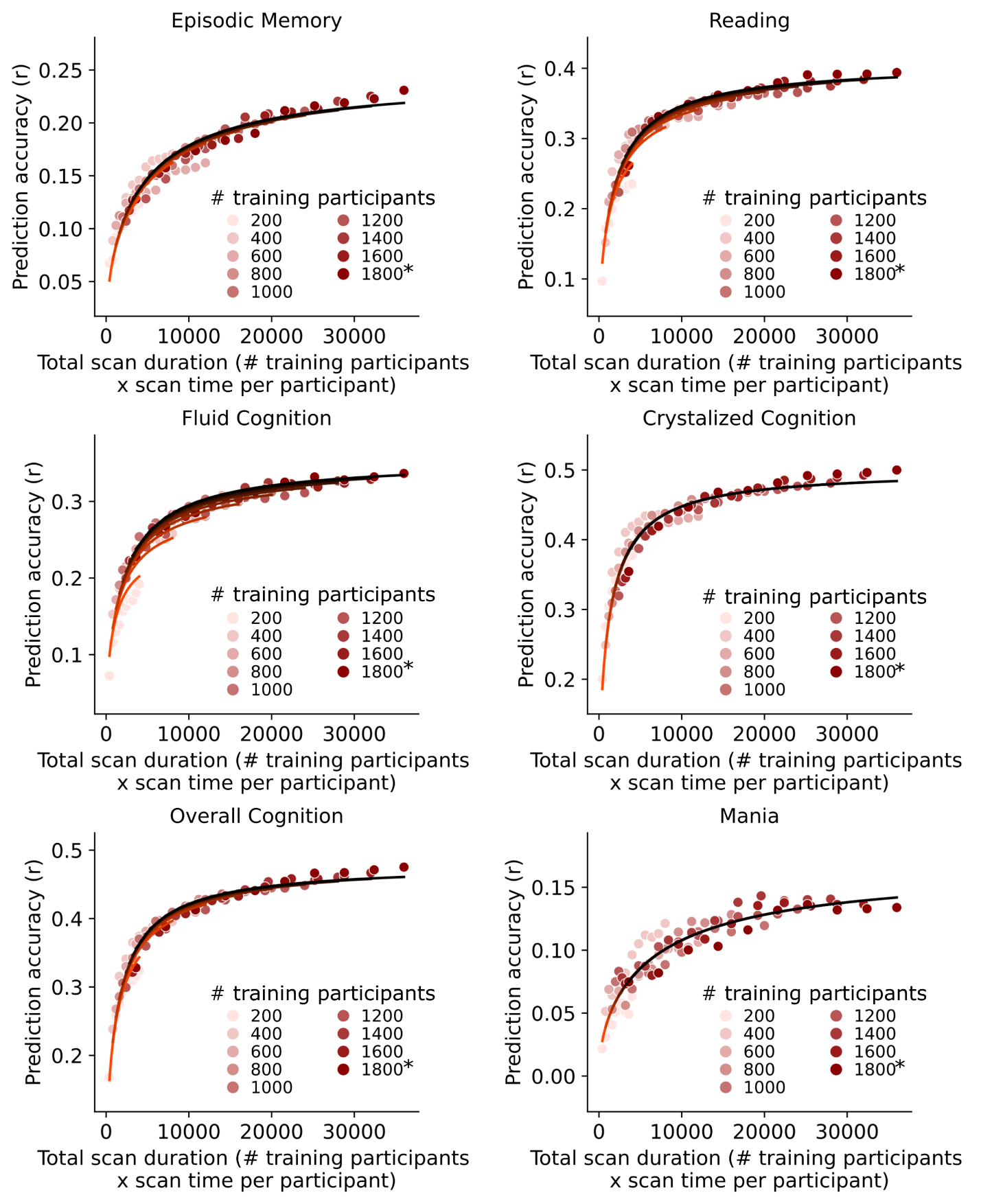


Supplementary Fig. 10.2 | Same as Fig. 3b except showing the scatter plots and the fit of the theoretical model for 6 of 17 phenotypic measures in the ABCD dataset that visually follow a logarithmic pattern. Scatter plot of prediction accuracy against total scan duration in the ABCD dataset. The curves were obtained by fitting the theoretical model to the prediction accuracies of the phenotype. The * in the figures indicates that all available participants were used, therefore the sample size will be close to, but not exactly the number shown.


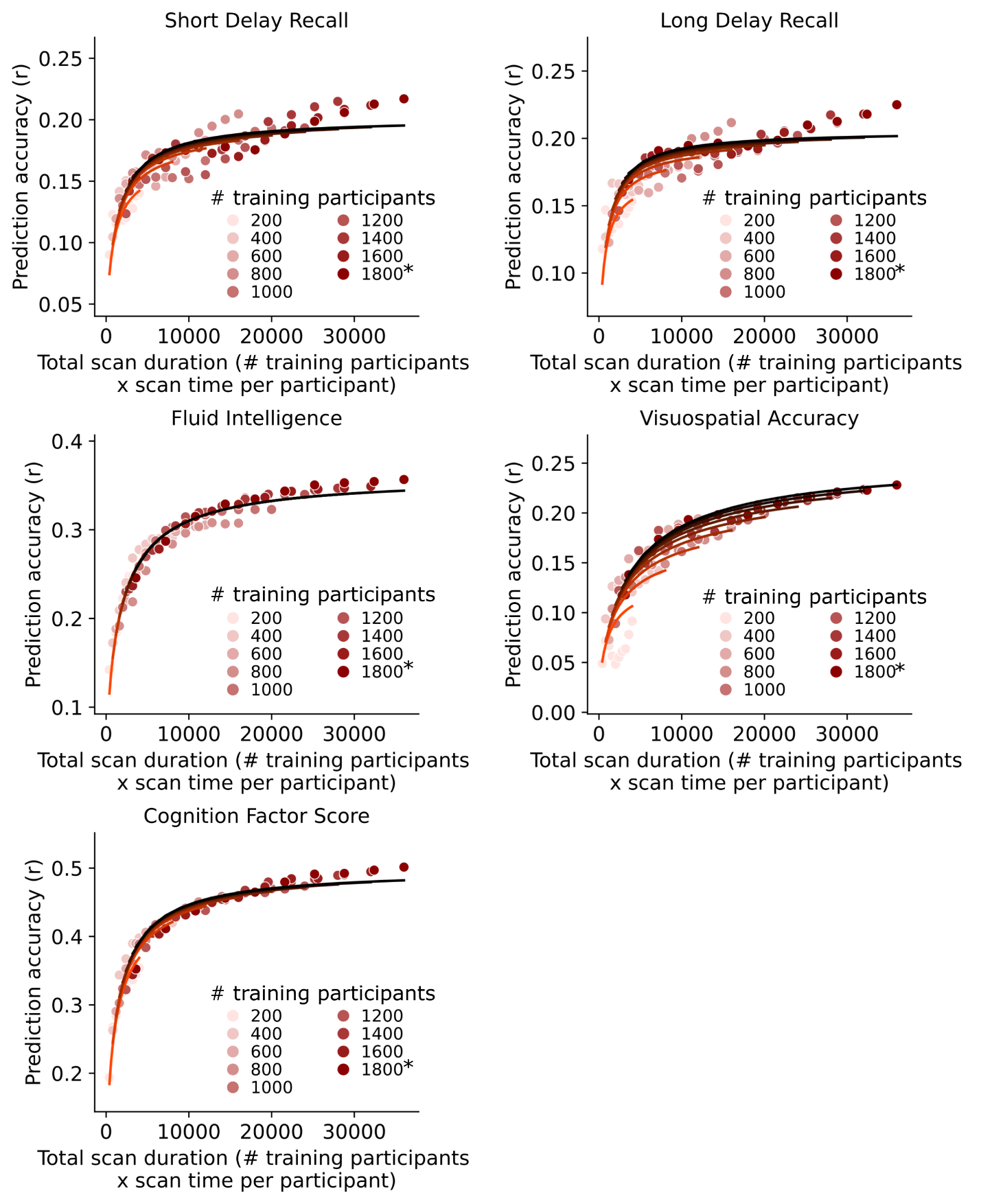


Supplementary Fig. 10.3 | Same as Fig. 3b except showing the scatter plots and the fit of the theoretical model for 5 of 17 phenotypic measures in the ABCD dataset that visually follow a logarithmic pattern. Scatter plot of prediction accuracy against total scan duration in the ABCD dataset. The curves were obtained by fitting the theoretical model to the prediction accuracies of the phenotype. The * in the figures indicates that all available participants were used, therefore the sample size will be close to, but not exactly the number shown.

Supplementary Fig. 11.1-11.4 | Theoretical model fit for 19 phenotypic measures in the HCP dataset.


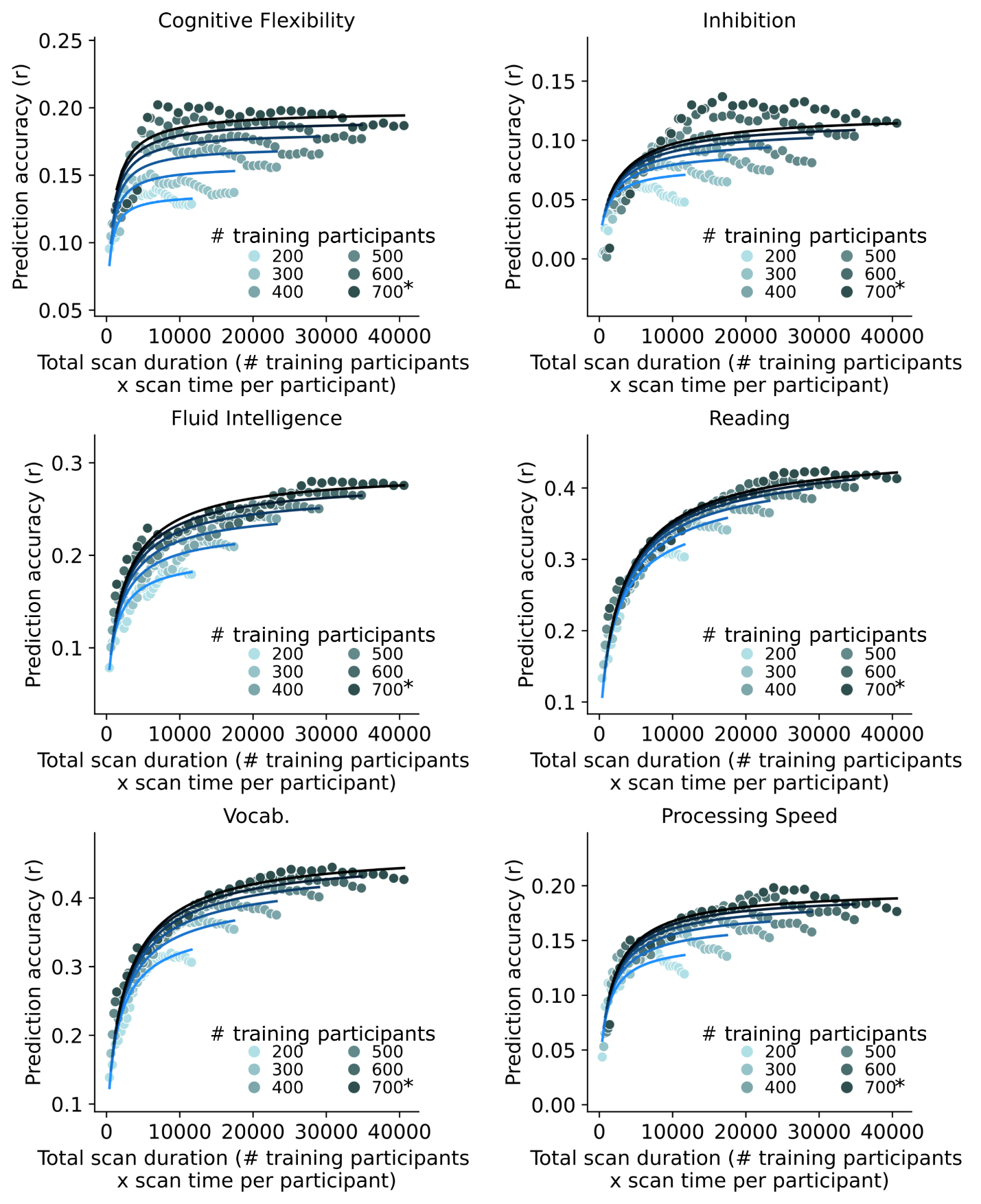
Supplementary Fig. 11.1 | Same as Fig. 3b except showing the scatter plots and the fit of the theoretical model for 6 of 19 phenotypic measures in the HCP dataset that visually follow a logarithmic pattern. Scatter plot of prediction accuracy against total scan duration in the HCP dataset. The curves were obtained by fitting the theoretical model to the prediction accuracies of the phenotype. The * in the figures indicates that all available participants were used, therefore the sample size will be close to, but not exactly the number shown.


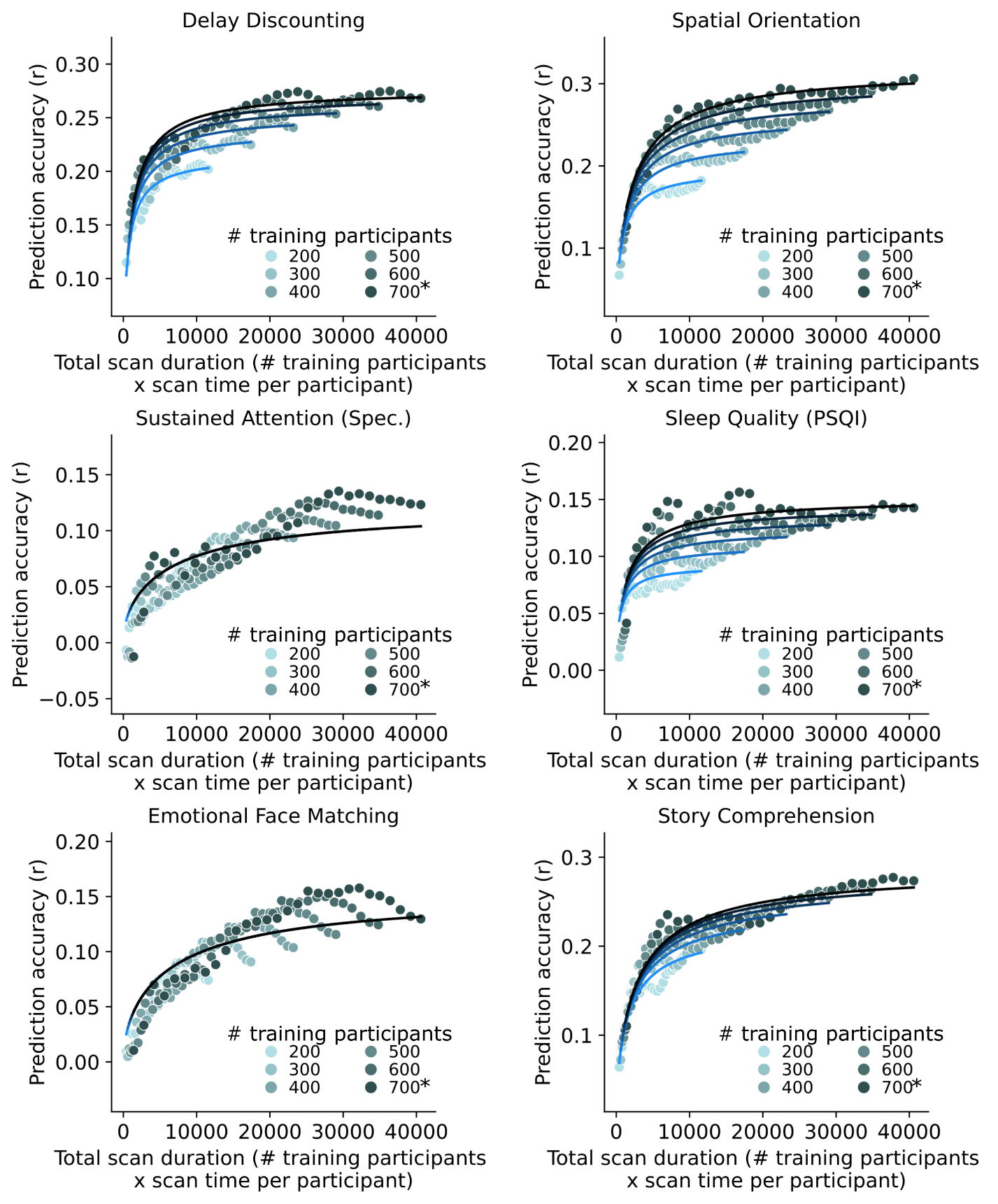


Supplementary Fig. 11.2 | Same as Fig. 3b except showing the scatter plots and the fit of the theoretical model for 6 of 19 phenotypic measures in the HCP dataset that visually follow a logarithmic pattern. Scatter plot of prediction accuracy against total scan duration in the HCP dataset. The curves were obtained by fitting the theoretical model to the prediction accuracies of the phenotype. The * in the figures indicates that all available participants were used, therefore the sample size will be close to, but not exactly the number shown.


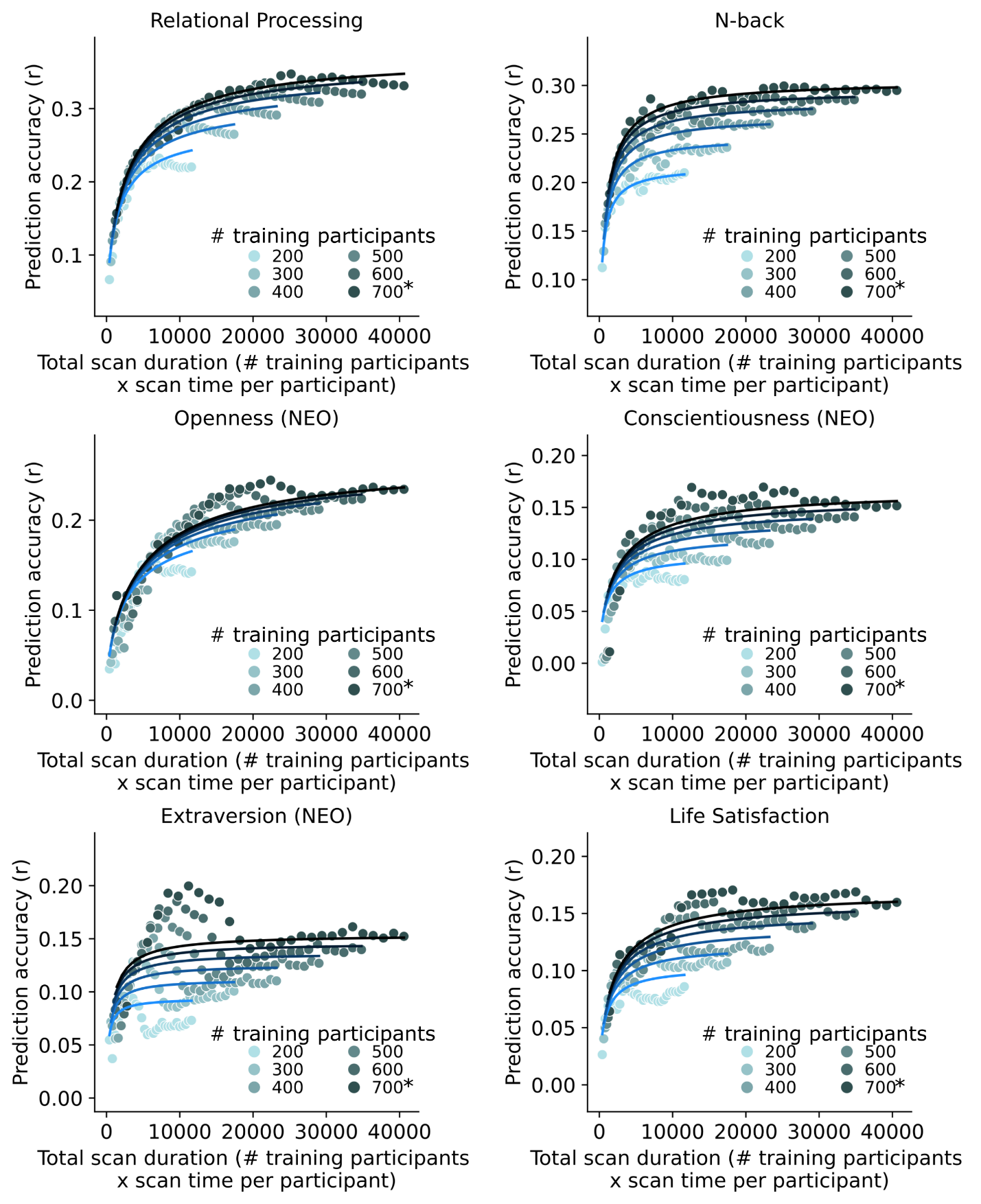


Supplementary Fig. 11.3 | Same as Fig. 3b except showing the scatter plots and the fit of the theoretical model for 6 of 19 phenotypic measures in the HCP dataset that visually follow a logarithmic pattern. Scatter plot of prediction accuracy against total scan duration in the HCP dataset. The curves were obtained by fitting the theoretical model to the prediction accuracies of the phenotype. The * in the figures indicates that all available participants were used, therefore the sample size will be close to, but not exactly the number shown.


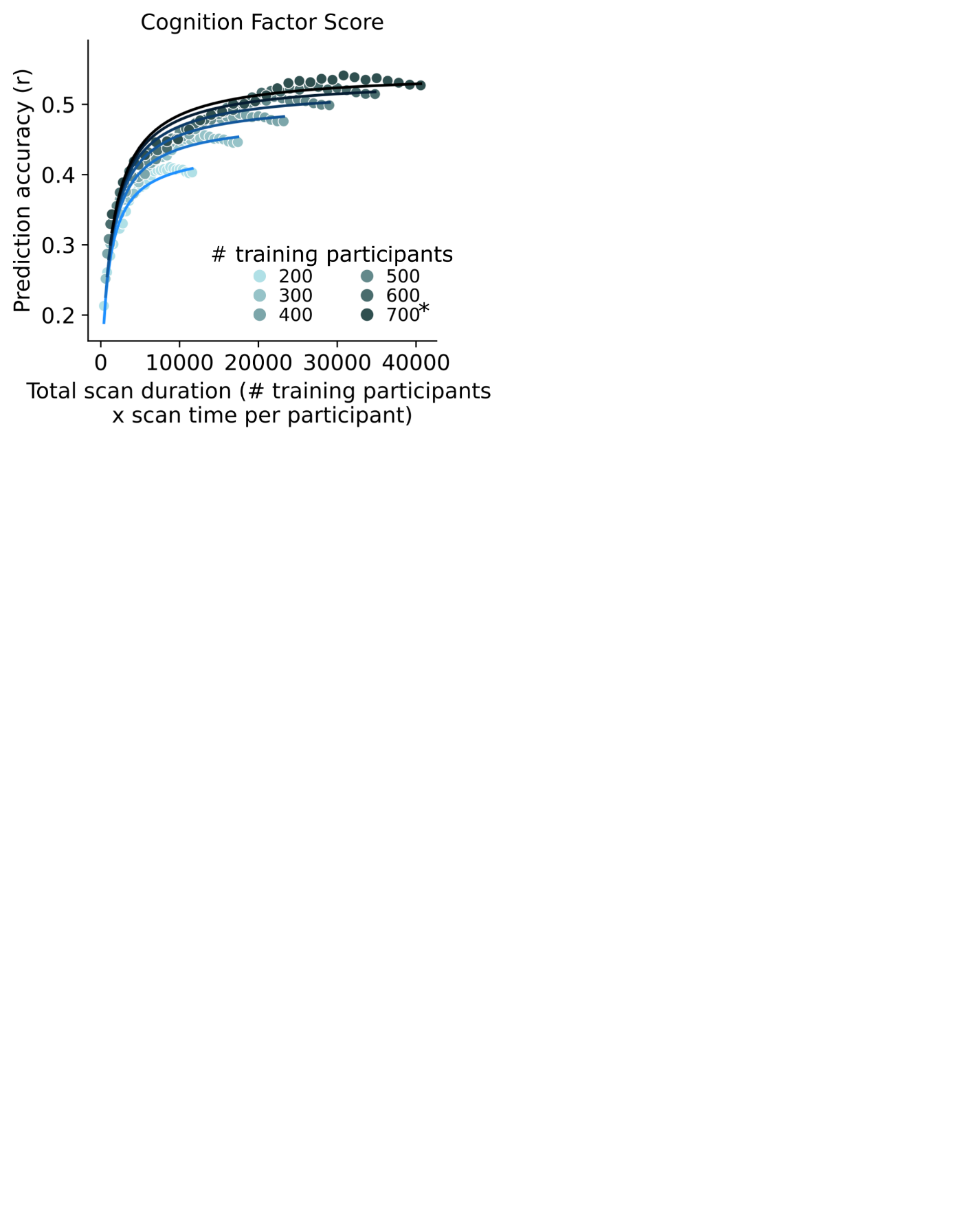


Supplementary Fig. 11.4 | Same as Fig. 3b except showing the scatter plots and the fit of the theoretical model for 1 of 19 phenotypic measures in the HCP dataset that visually follow a logarithmic pattern. Scatter plot of prediction accuracy against total scan duration in the HCP dataset. The curves were obtained by fitting the theoretical model to the prediction accuracies of the phenotype. The * in the figures indicates that all available participants were used, therefore the sample size will be close to, but not exactly the number shown.


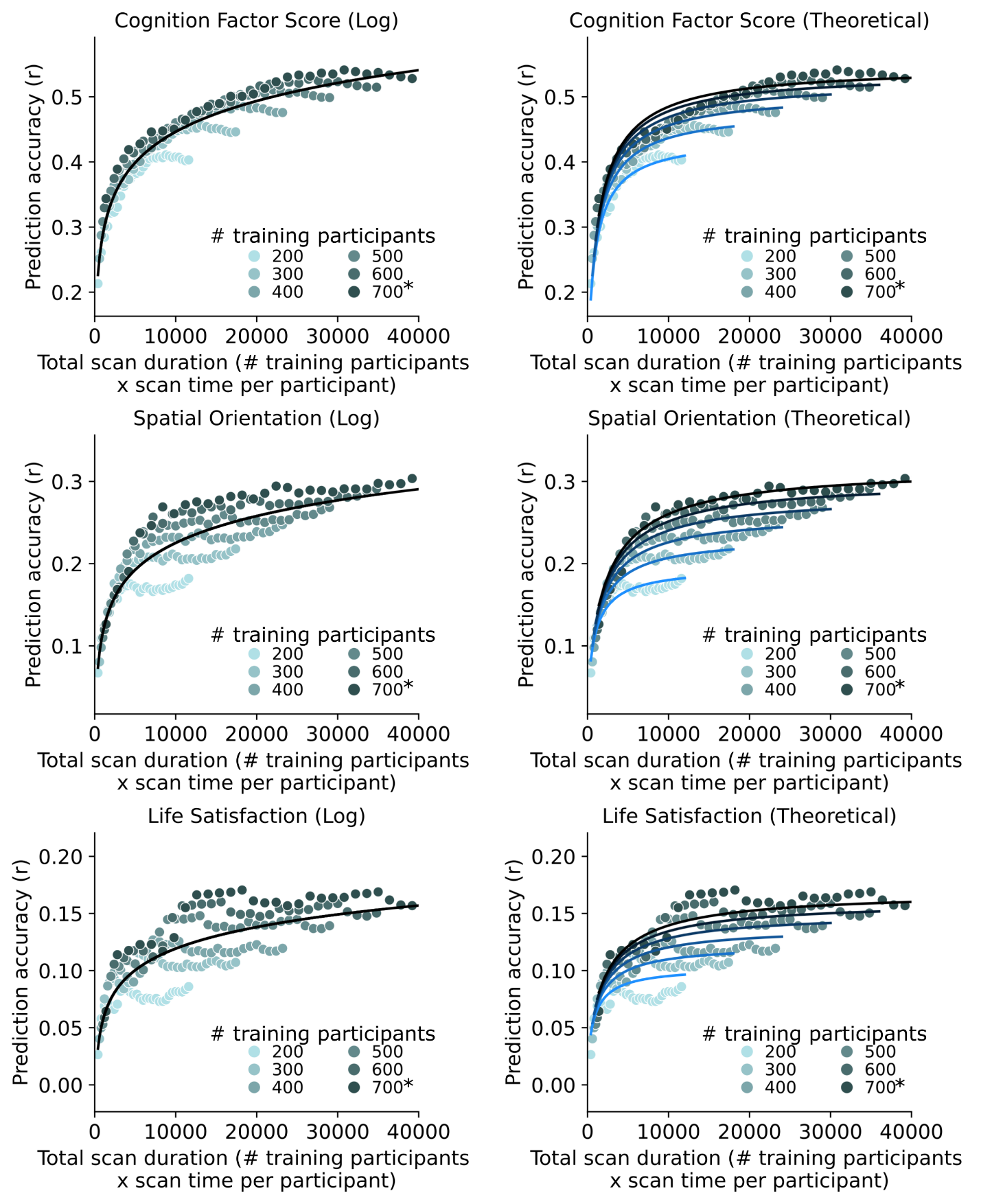


### Supplementary Fig. 12 | Theoretical model outperforms logarithmic model for longer scan times.

Visual comparison of logarithmic and theoretical models fitted to three HCP phenotypes using the full 58 minutes of data. Because the logarithm model treated the sample size N and scan time T as being interchangeable, it was not able to explain the diminishing returns of scan time T relative to sample size N for larger values of T.

Supplementary Fig. 13.1-13.3 | Theoretical model fit for 14 phenotypic measures in the SINGER dataset.


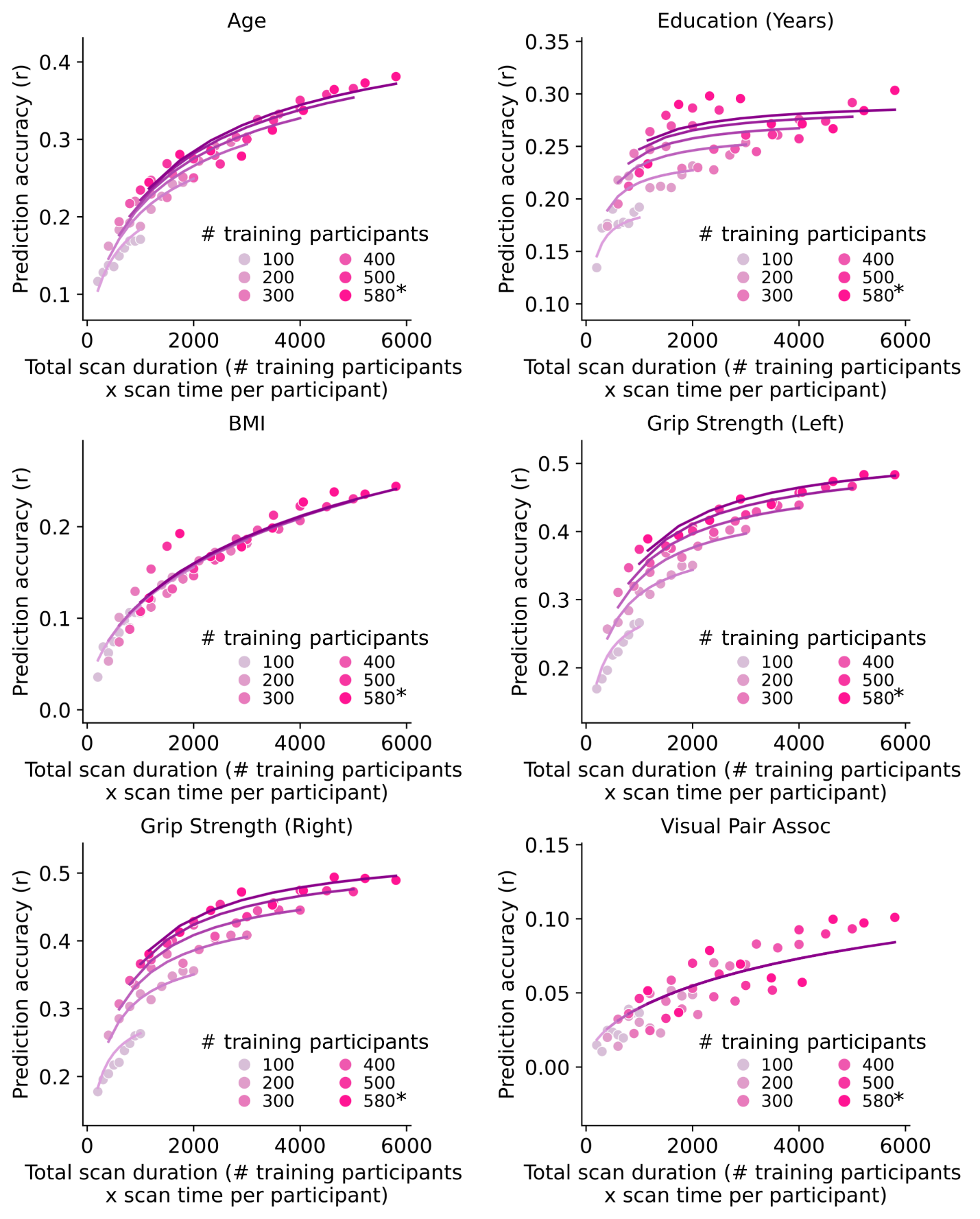


Supplementary Fig. 13.1 | Same as Fig. 3b except showing the scatter plots and the fit of the theoretical model for 6 of 14 phenotypic measures in the SINGER dataset that exhibit a good fit to the theoretical model. The curves were obtained by fitting the theoretical model to the prediction accuracies of the phenotype. The * in the figures indicates that all available participants were used, therefore the sample size will be close to, but not exactly the number shown.


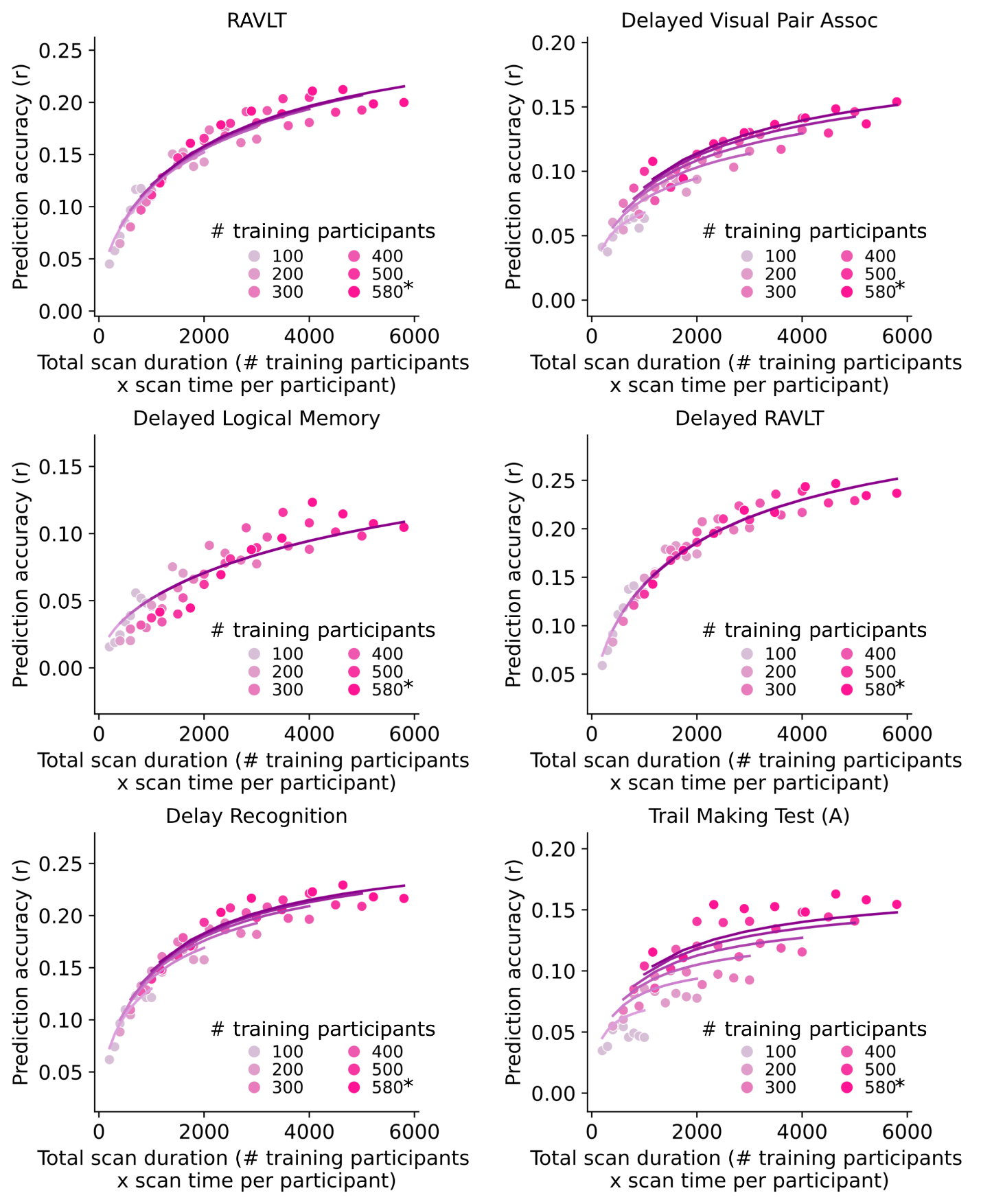


Supplementary Fig. 13.2 | Same as Fig. 3b except showing the scatter plots and the fit of the theoretical model for 6 of 14 phenotypic measures in the SINGER dataset that exhibit a good fit to the theoretical model. The curves were obtained by fitting the theoretical model to the prediction accuracies of the phenotype. The * in the figures indicates that all available participants were used, therefore the sample size will be close to, but not exactly the number shown.


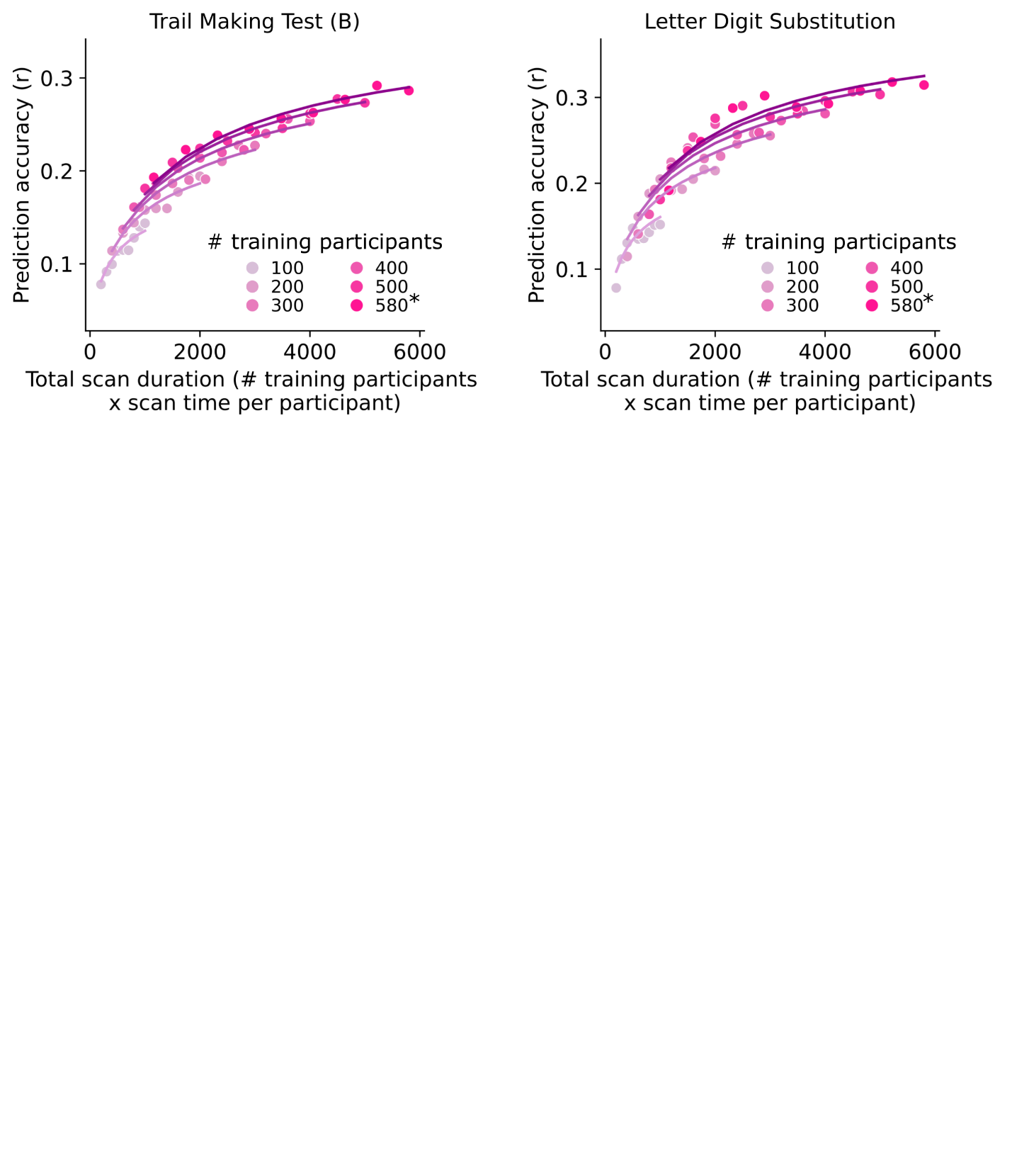


Supplementary Fig. 13.3 | Same as Fig. 3b except showing the scatter plots and the fit of the theoretical model for 2 of 14 phenotypic measures in the SINGER dataset that exhibit a good fit to the theoretical model. The curves were obtained by fitting the theoretical model to the prediction accuracies of the phenotype. The * in the figures indicates that all available participants were used, therefore the sample size will be close to, but not exactly the number shown.

Supplementary Fig. 14.1-14.2 | Theoretical model fit for 7 phenotypic measures in the TCP dataset.


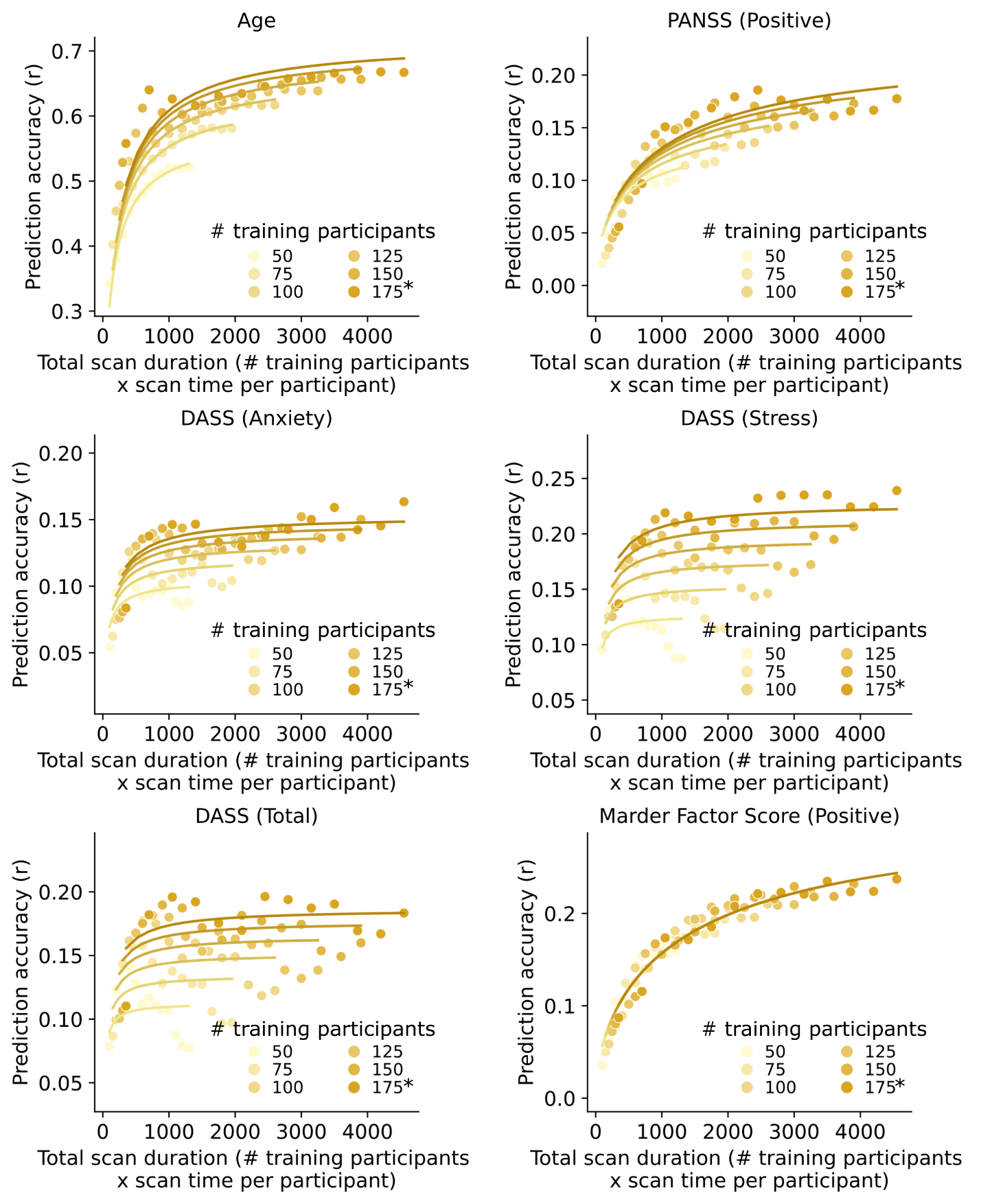
Supplementary Fig. 14.1 | Same as Fig. 3b except showing the scatter plots and the fit of the theoretical model for 6 of 7 phenotypic measures in the TCP dataset. The curves were obtained by fitting the theoretical model to the prediction accuracies of the phenotype. The * in the figures indicates that all available participants were used, therefore the sample size will be close to, but not exactly the number shown.


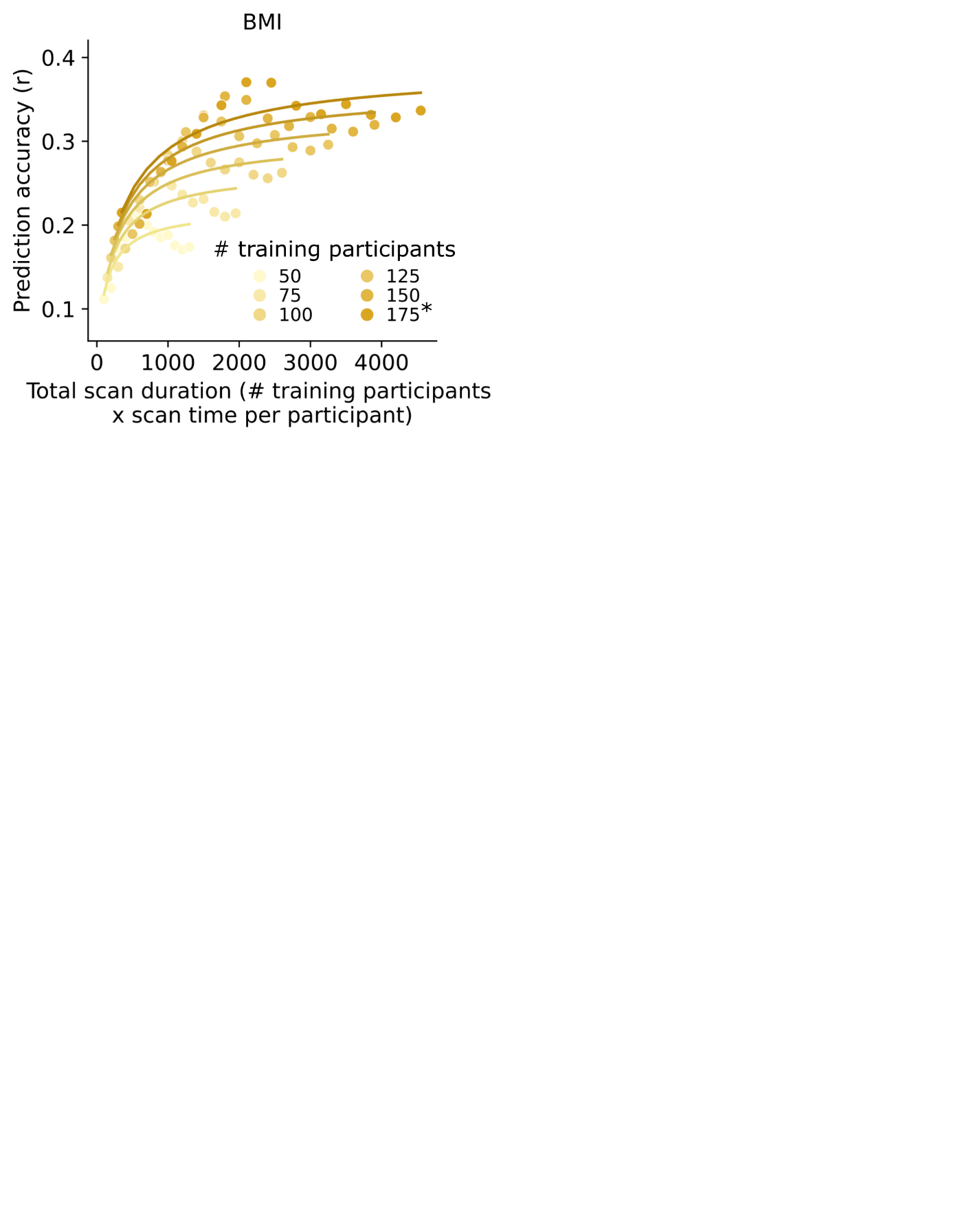
Supplementary Fig. 14.2 | Same as Fig. 3b except showing the scatter plots and the fit of the theoretical models for 1 of 7 phenotypic measures in the TCP dataset. The curves were obtained by fitting the theoretical model to the prediction accuracies of the phenotype. The * in the figures indicates that all available participants were used, therefore the sample size will be close to, but not exactly the number shown.

Supplementary Fig. 15.1-15.2 | Theoretical model fit for 7 phenotypic measures in the MDD dataset.


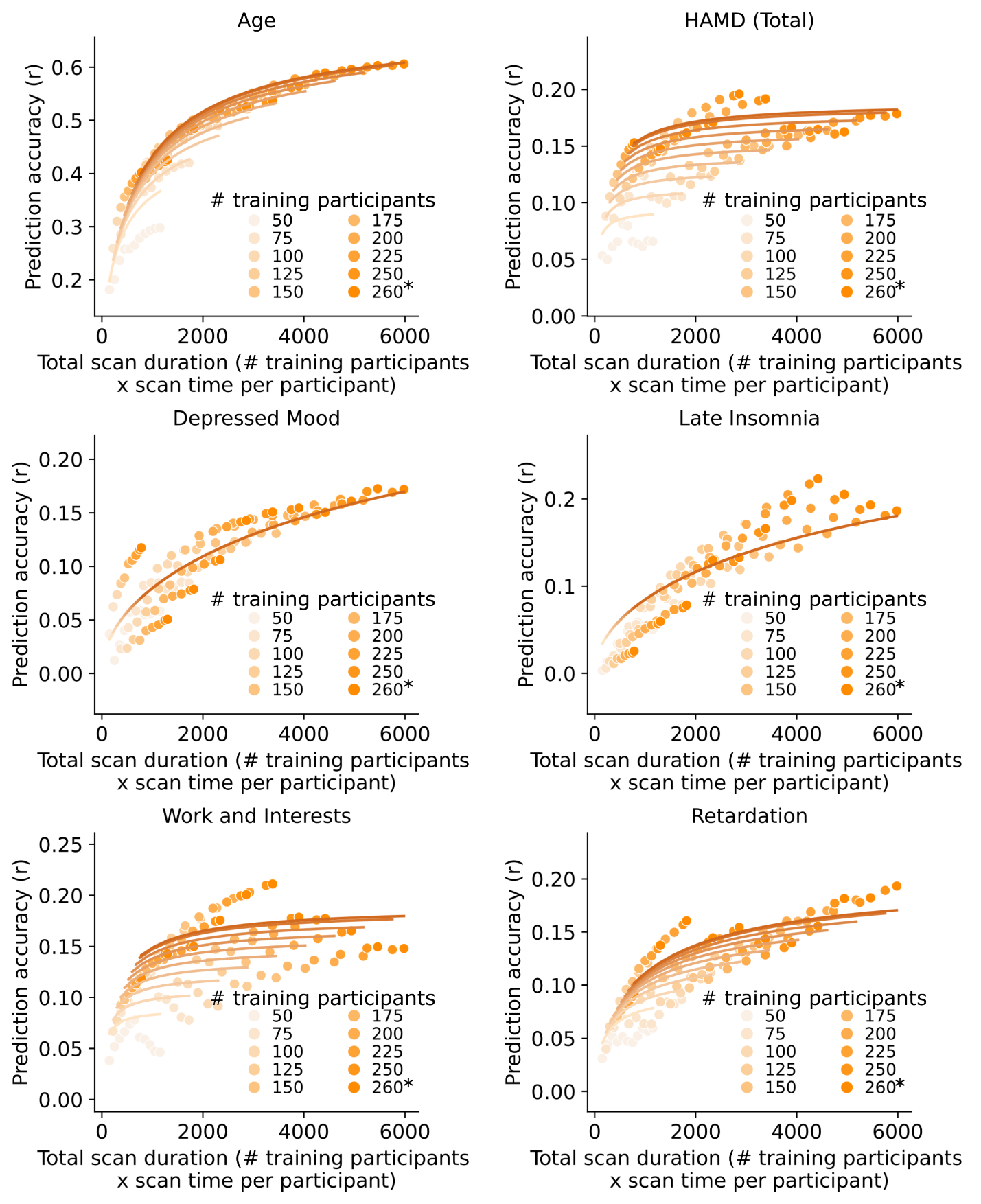
Supplementary Fig. 15.1 | Same as Fig. 3b except showing the scatter plots and the fit of the theoretical model for 6 of 7 phenotypic measures in the MDD dataset. The curves were obtained by fitting the theoretical model to the prediction accuracies of the phenotype. The * in the figures indicates that all available participants were used, therefore the sample size will be close to, but not exactly the number shown.


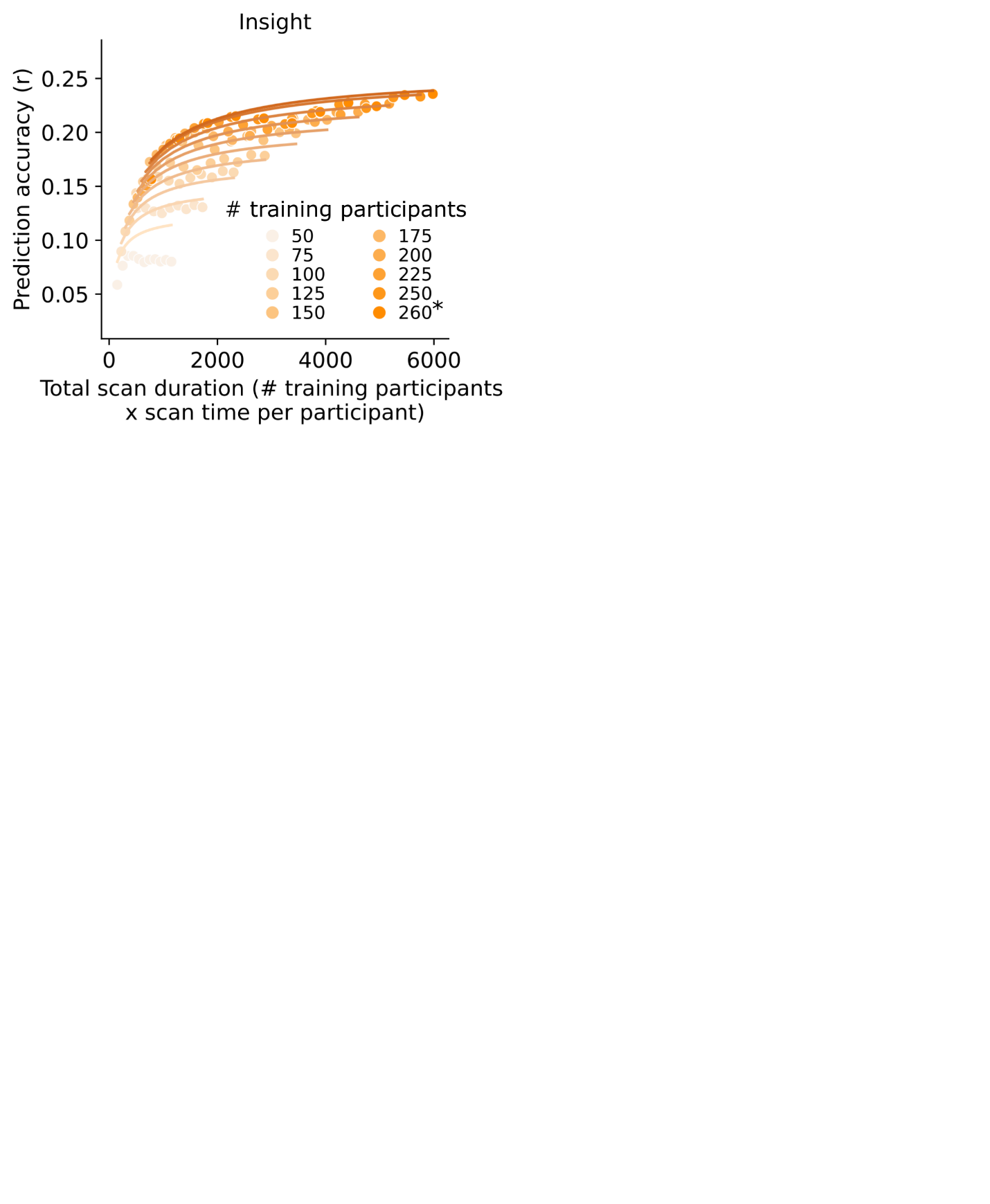
Supplementary Fig. 15.2 | Same as Fig. 3b except showing the scatter plots and the fit of the theoretical model for 1 of 7 phenotypic measures in the MDD dataset. The curves were obtained by fitting the theoretical model to the prediction accuracies of the phenotype. The * in the figures indicates that all available participants were used, therefore the sample size will be close to, but not exactly the number shown.


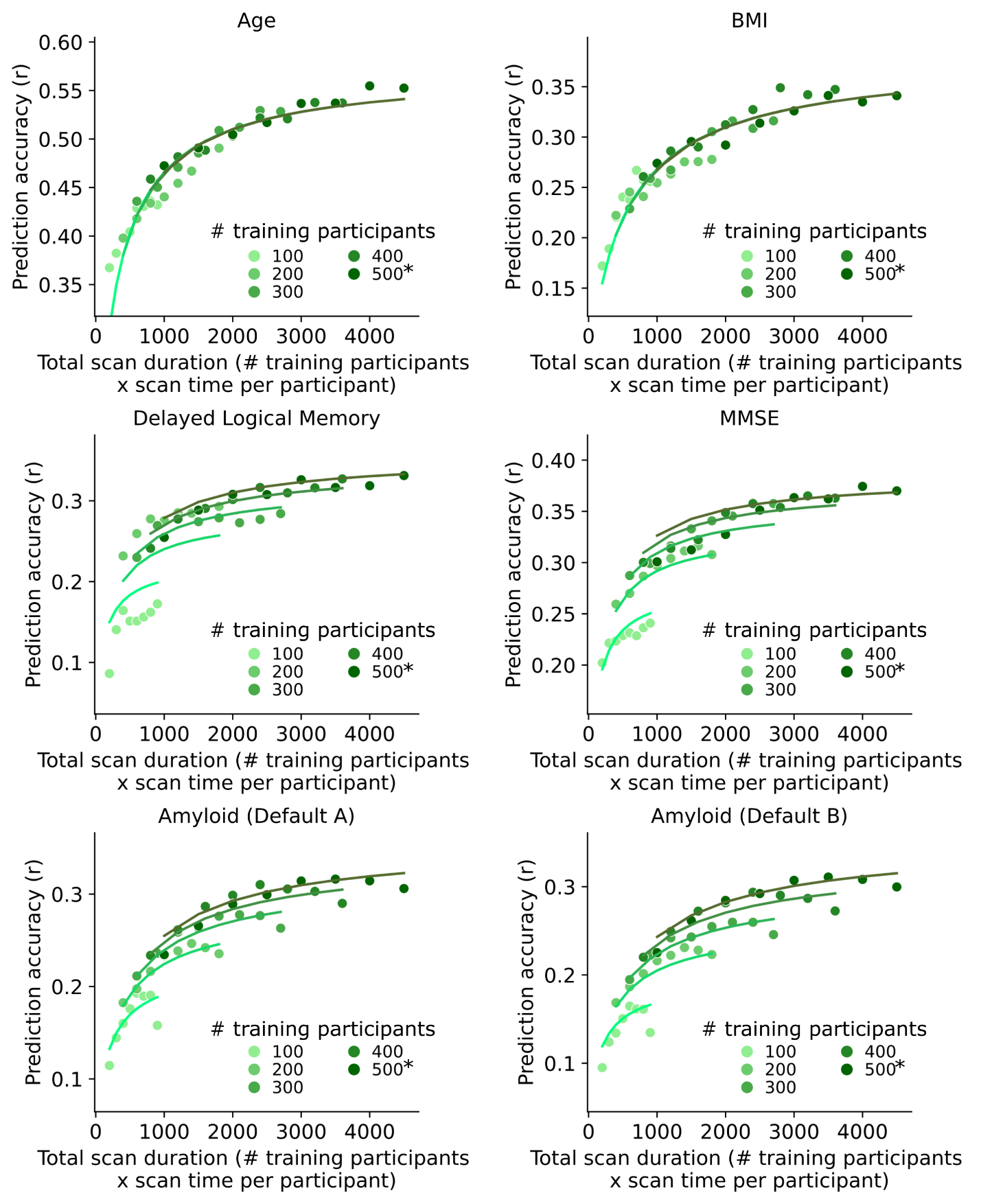


### Supplementary Fig. 16 | Theoretical model fit for 6 phenotypic measures in the ADNI dataset.

Same as Fig. 3b except showing the scatter plots and the fit of the theoretical model for 6 of 6 phenotypic measures in the Alzheimer’s Disease Neuroimaging Initiative (ADNI) dataset. The curves were obtained by fitting the theoretical model to the prediction accuracies of the phenotype. The * in the figures indicates that all available participants were used, therefore the sample size will be close to, but not exactly the number shown.

Supplementary Fig. 17.1-17.3 | Theoretical model fit for 16 phenotypic measures using the ABCD MID task data.


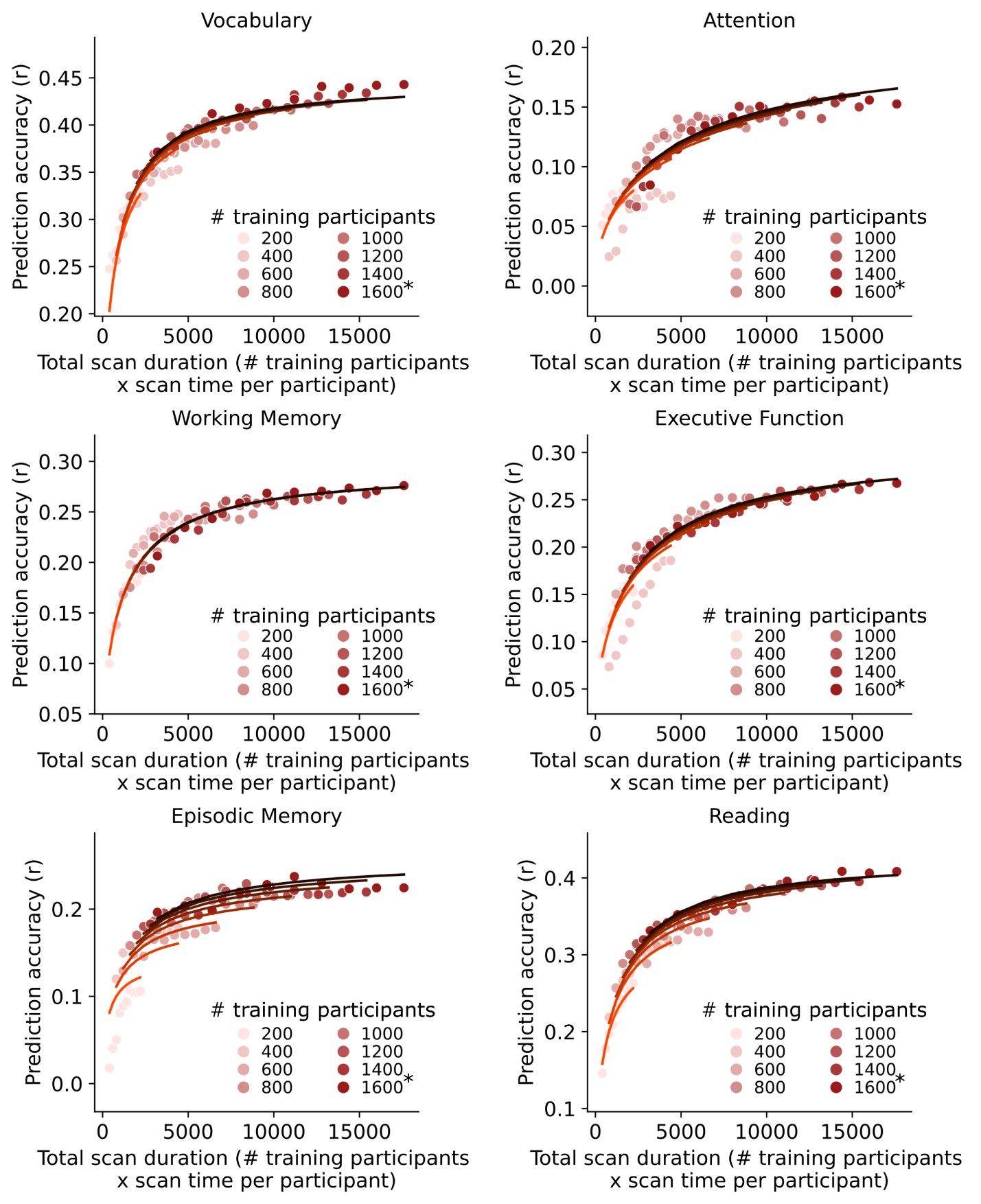
Supplementary Fig. 17.1 | Same as Fig. 3b except showing the scatter plots and the fit of the theoretical model for 6 of 16 phenotypic measures using Monetary Incentive Delay (MID) task-FC in the ABCD dataset. The curves were obtained by fitting the theoretical model to the prediction accuracies of the phenotype. The * in the figures indicates that all available participants were used, therefore the sample size will be close to, but not exactly the number shown.


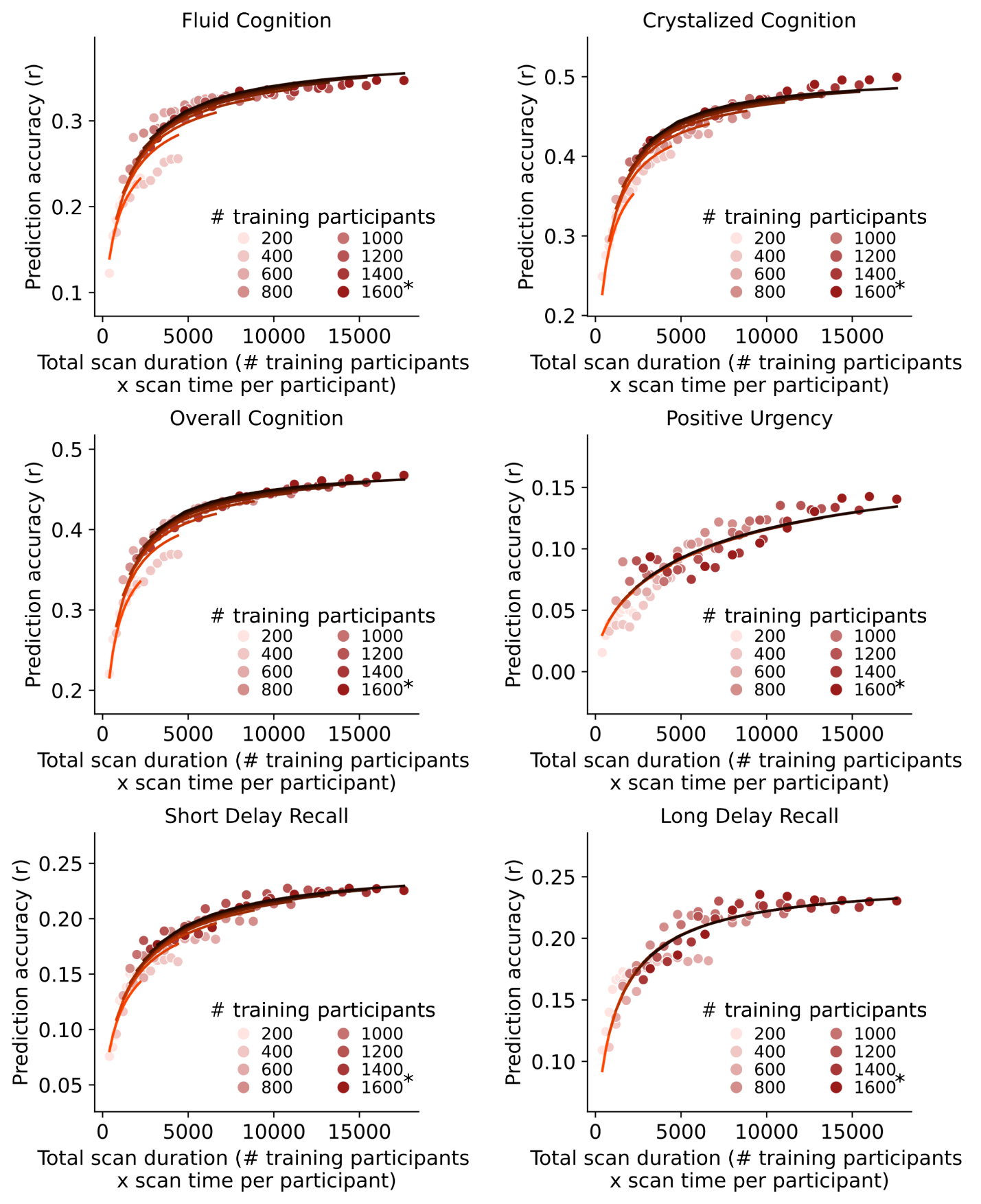
Supplementary Fig. 17.2 | Same as Fig. 3b except showing the scatter plots and the fit of the theoretical model for 6 of 16 phenotypic measures using Monetary Incentive Delay (MID) task-FC in the ABCD dataset. The curves were obtained by fitting the theoretical model to the prediction accuracies of the phenotype. The * in the figures indicates that all available participants were used, therefore the sample size will be close to, but not exactly the number shown.


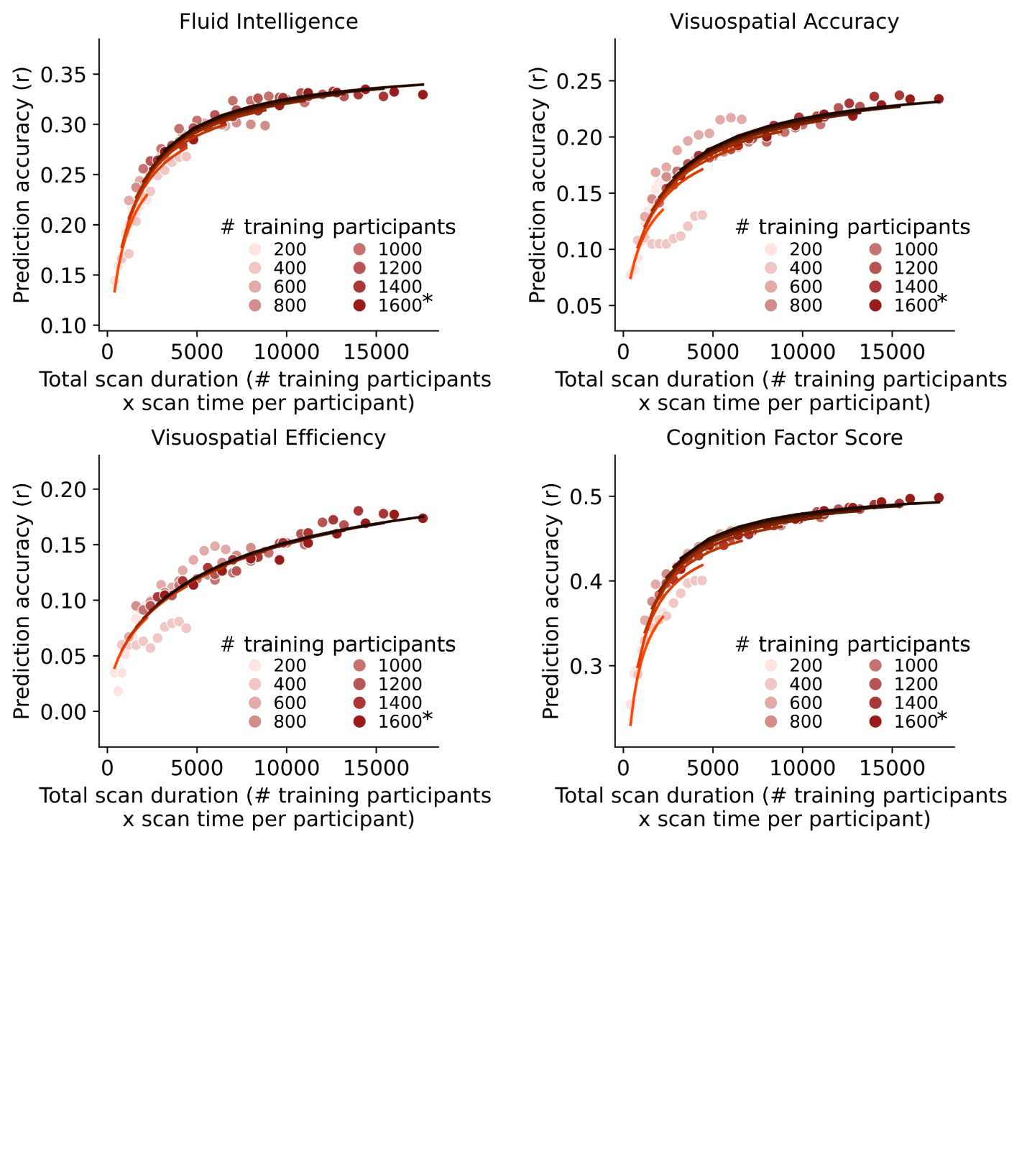


Supplementary Fig. 17.3 | Same as Fig. 3b except showing the scatter plots and the fit of the theoretical model for 4 of 16 phenotypic measures using Monetary Incentive Delay (MID) task-FC in the ABCD dataset. The curves were obtained by fitting the theoretical model to the prediction accuracies of the phenotype. The * in the figures indicates that all available participants were used, therefore the sample size will be close to, but not exactly the number shown.

Supplementary Fig. 18.1-18.4 | Theoretical model fit for 19 phenotypic measures using the ABCD N-back task data.


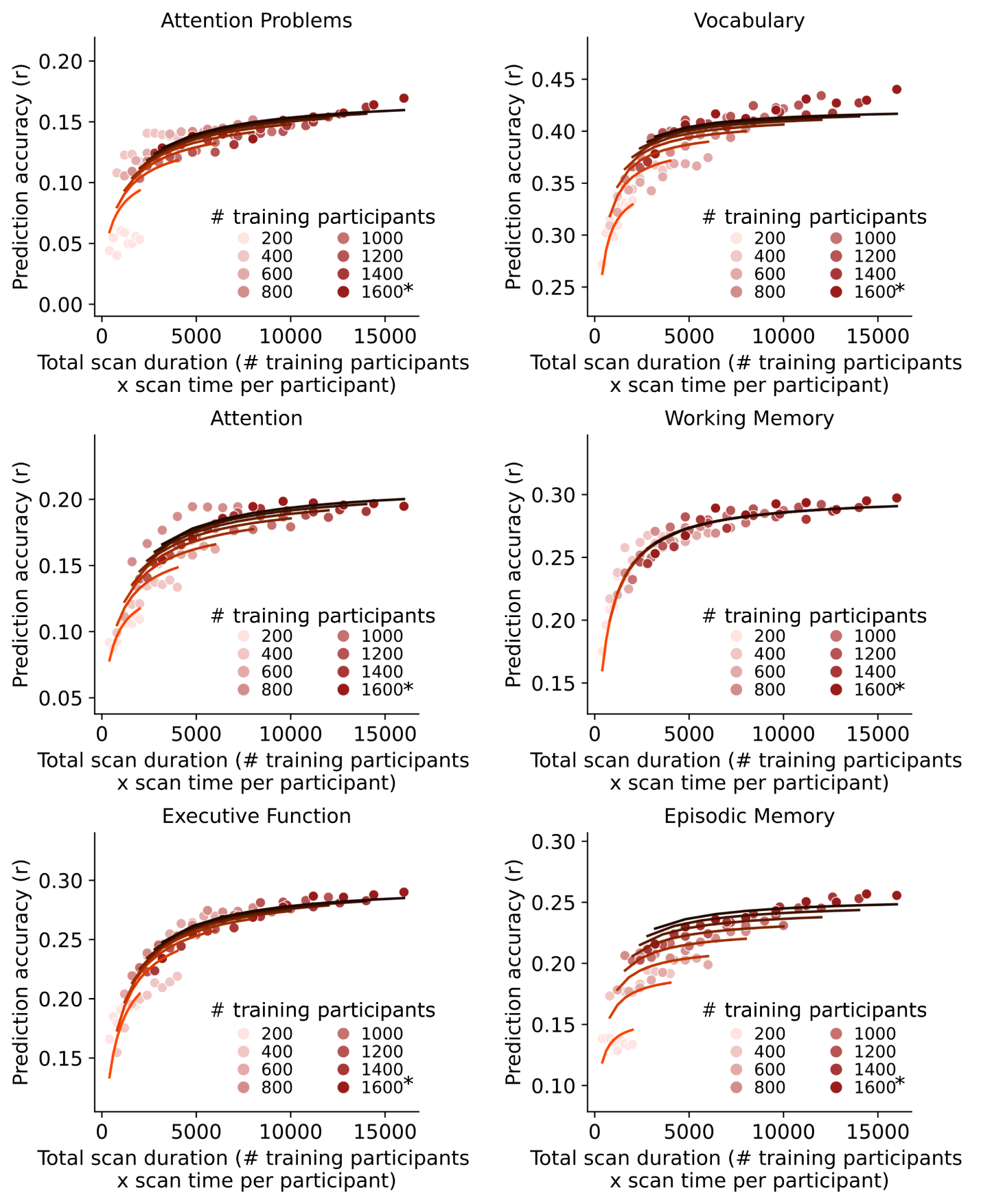
Supplementary Fig. 18.1 | Same as Fig. 3b except showing the scatter plots and the fit of the theoretical model for 6 of 19 phenotypic measures for the N-Back Task in the ABCD dataset. The curves were obtained by fitting the theoretical model to the prediction accuracies of the phenotype. The * in the figures indicates that all available participants were used, therefore the sample size will be close to, but not exactly the number shown.


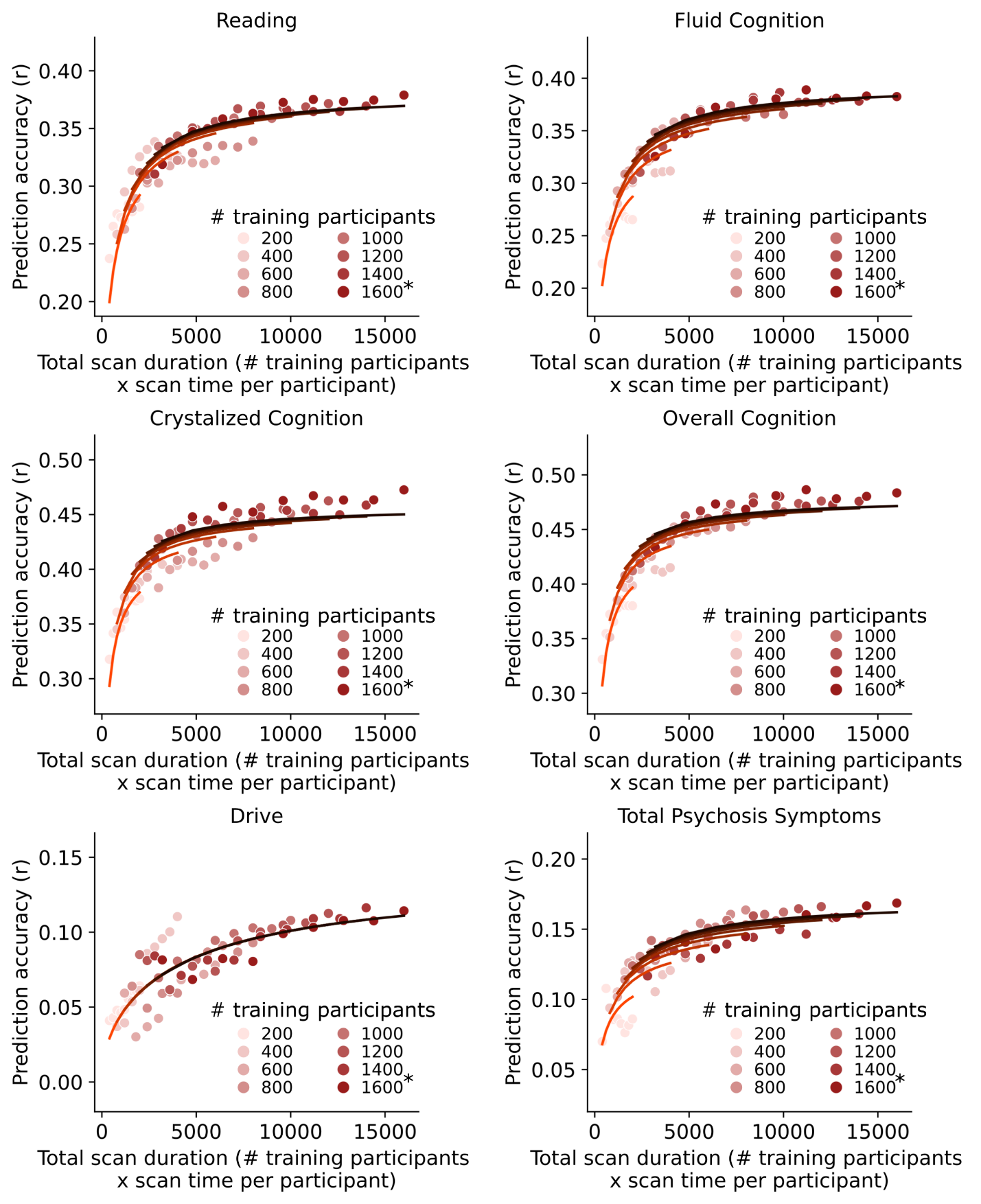


Supplementary Fig. 18.2 | Same as Fig. 3b except showing the scatter plots and the fit of the theoretical model for 6 of 19 phenotypic measures for the N-Back Task in the ABCD dataset. The curves were obtained by fitting the theoretical model to the prediction accuracies of the phenotype. The * in the figures indicates that all available participants were used, therefore the sample size will be close to, but not exactly the number shown.


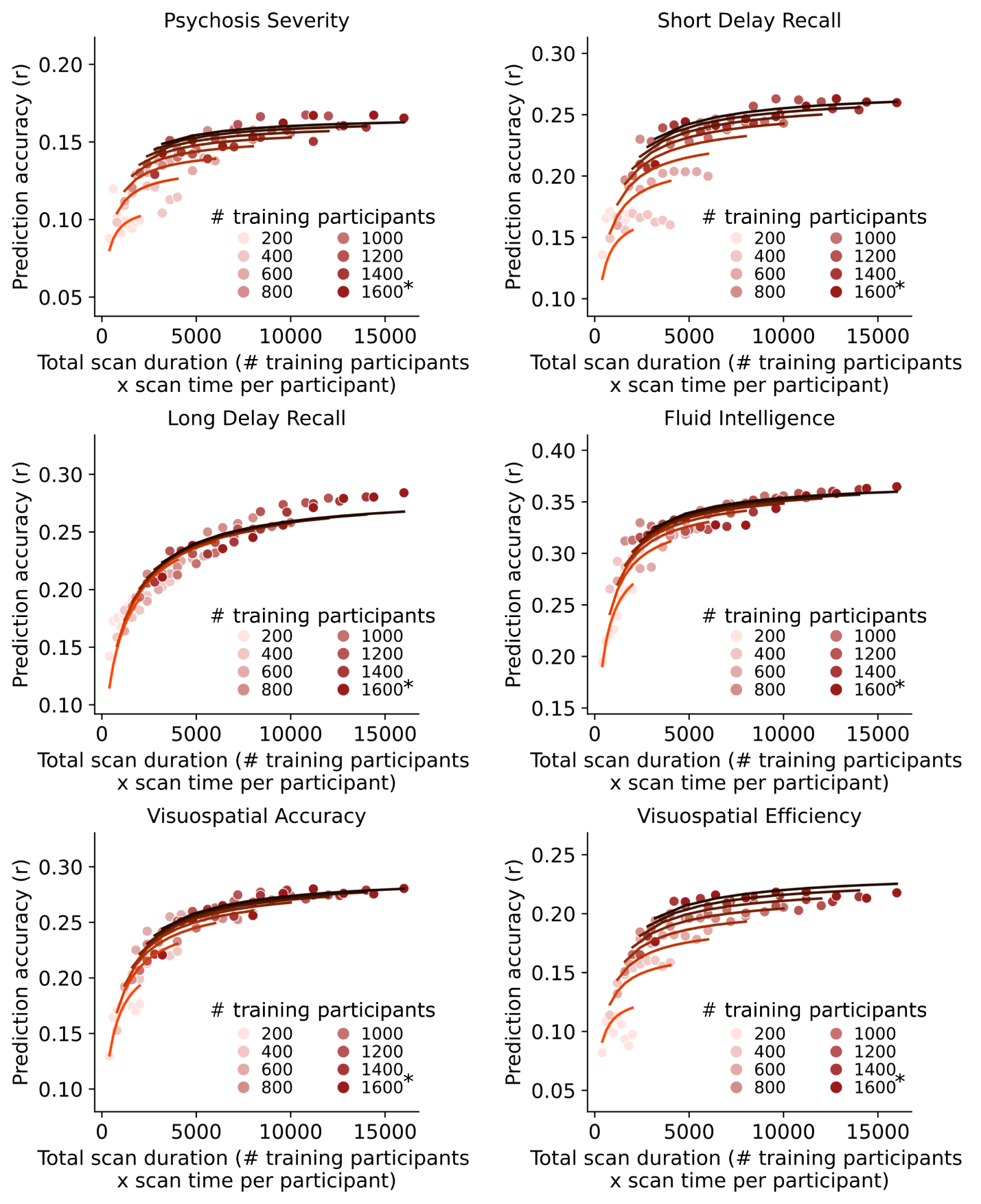


Supplementary Fig. 18.3 | Same as Fig. 3b except showing the scatter plots and the fit of the theoretical model for 6 of 19 phenotypic measures for the N-Back Task in the ABCD dataset. The curves were obtained by fitting the theoretical model to the prediction accuracies of the phenotype. The * in the figures indicates that all available participants were used, therefore the sample size will be close to, but not exactly the number shown.


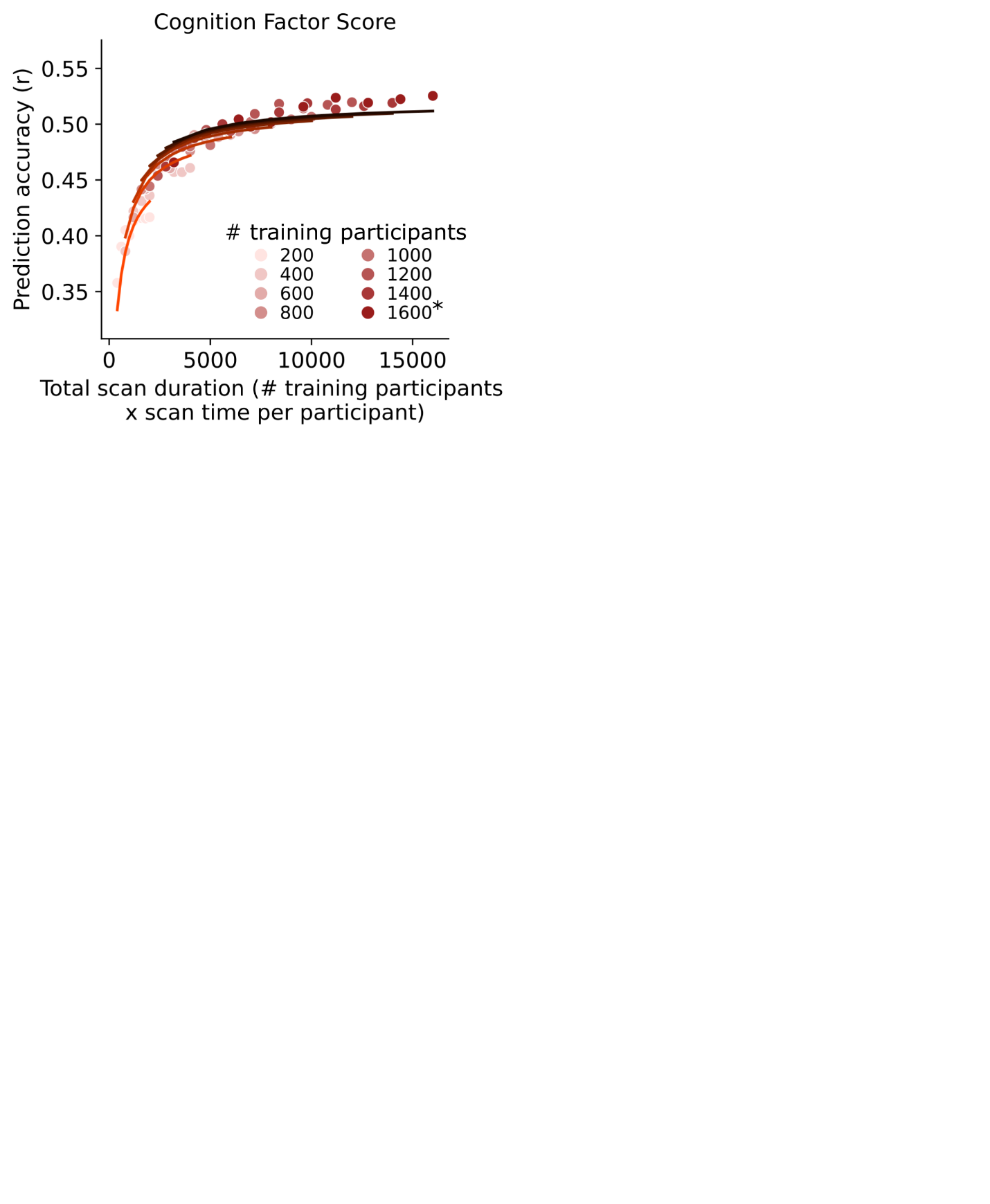


Supplementary Fig. 18.4 | Same as Fig. 3b except showing the scatter plots and the fit of the theoretical model for 1 of 19 phenotypic measures for the N-Back Task in the ABCD dataset. The curves were obtained by fitting the theoretical model to the prediction accuracies of the phenotype. The * in the figures indicates that all available participants were used, therefore the sample size will be close to, but not exactly the number shown.

Supplementary Fig. 19.1-19.3 | Theoretical model fit for 18 phenotypic measures using the ABCD SST task data.


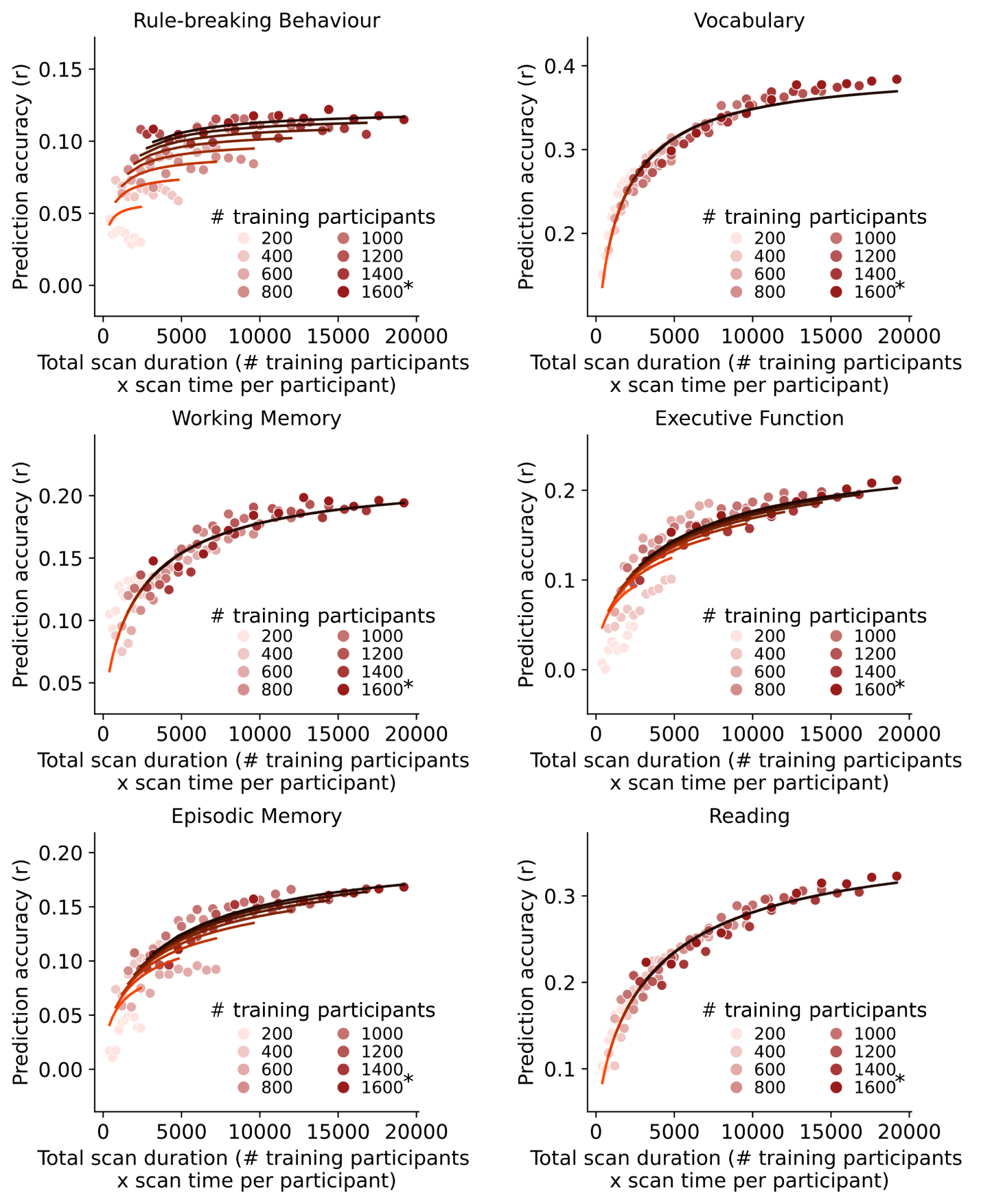
Supplementary Fig. 19.1 | Same as Fig. 3b except showing the scatter plots and the fit of the theoretical model for 6 of 18 phenotypic measures for the Stop Signal Task (SST) in the ABCD dataset. The curves were obtained by fitting the theoretical model to the prediction accuracies of the phenotype. We only showed phenotypes that exhibited good fit with the theoretical model (Supplementary Table 2). The * in the figures indicates that all available participants were used, therefore the sample size will be close to, but not exactly the number shown.


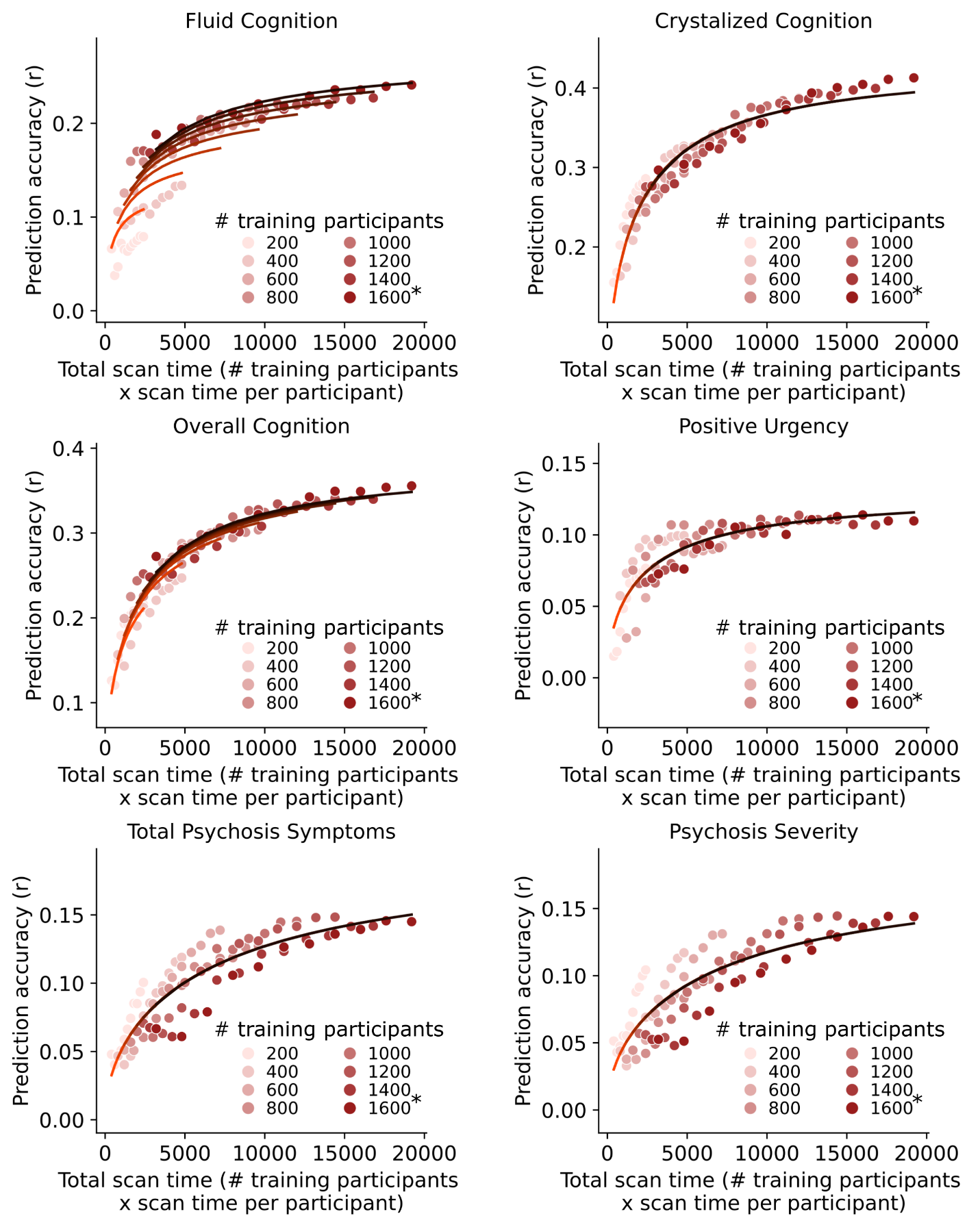
Supplementary Fig. 19.2 | Same as Fig. 3b except showing the scatter plots and the fit of the theoretical model for 6 of 18 phenotypic measures for the Stop Signal Task (SST) in the ABCD dataset. The curves were obtained by fitting the theoretical model to the prediction accuracies of the phenotype. The * in the figures indicates that all available participants were used, therefore the sample size will be close to, but not exactly the number shown.


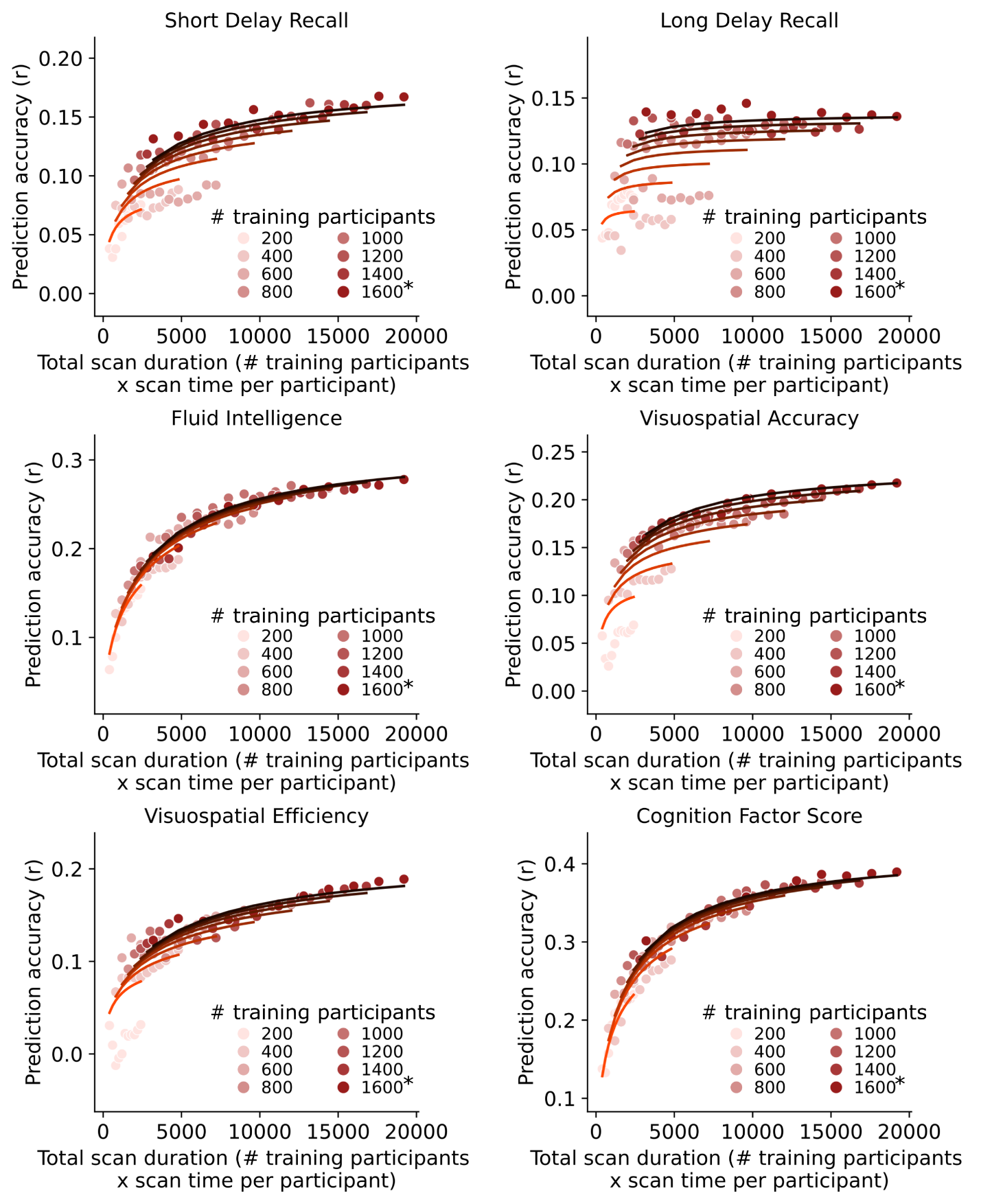
Supplementary Fig. 19.3 | Same as Fig. 3b except showing the scatter plots and the fit of the theoretical model for 6 of 18 phenotypic measures for the Stop Signal Task (SST) in the ABCD dataset. The curves were obtained by fitting the theoretical model to the prediction accuracies of the phenotype. The * in the figures indicates that all available participants were used, therefore the sample size will be close to, but not exactly the number shown.


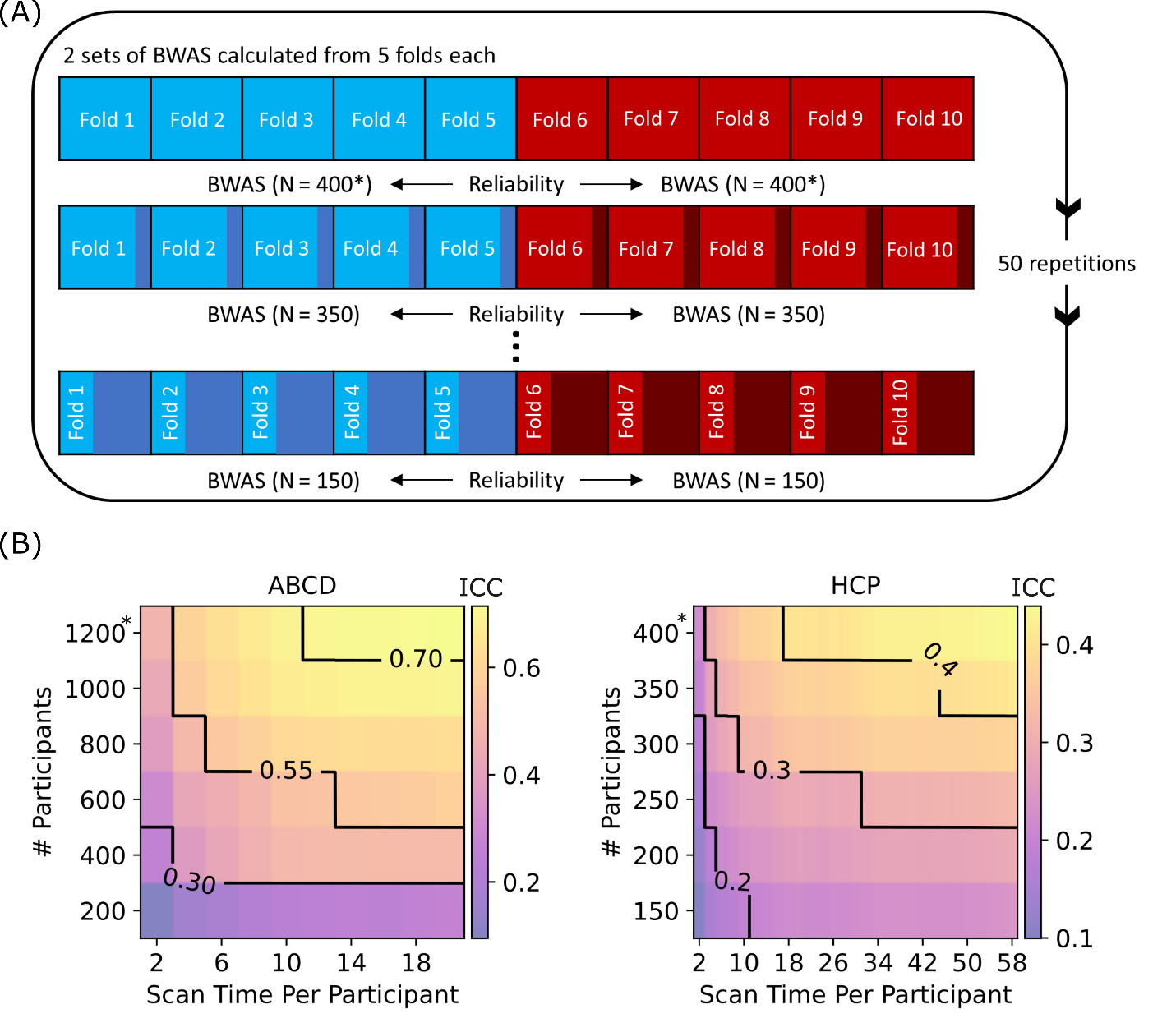


### Supplementary Fig. 20 | Reliability split-half procedure.

a. Reliability analysis workflow for the HCP dataset. The participants were split into 2 sets. Univariate brain-wide association (BWAS) was performed on each set and the agreement (i.e., split-half reliability) between the two sets was computed based on the intra-class correlation metric (see Methods). To vary sample size, each set was subsampled and the whole procedure was repeated. Finally, the procedure was repeated with different amount of fMRI data *T* (not shown in panel) and 50 times for stability. A similar workflow was used in the ABCD dataset. Similar to the prediction analysis (Extended Data Fig. 1), in the case of HCP, care was taken so siblings were not split across sets, while in the case of ABCD, participants from the same site were not split across sets. See Methods for details. b. Contour plot of univariate brain-wide association analyses (BWAS) reliability (intra-class correlation) of the cognitive factor score as a function of the scan time used to generate the functional connectivity matrix (x-axis), and the number of training participants used to train the predictive model (y-axis) in the ABCD and HCP datasets. Increasing training participants and scan time both led to increases in split-half reliability. The * in both figures indicates that all available participants were used, therefore the sample size will be close to, but not exactly the number shown.


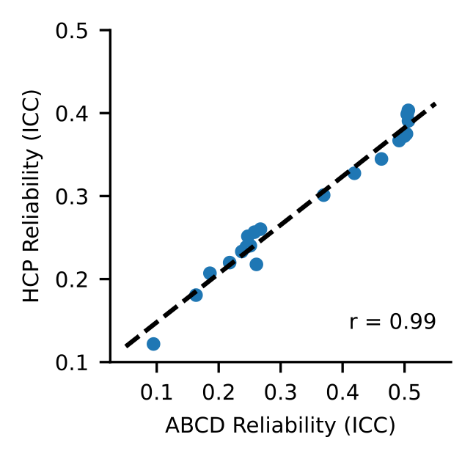


### Supplementary Fig. 21 | Correlation between univariate BWAS reliability of ABCD and HCP cognition factor scores.

Scatter plot of the cognition factor univariate reliability (ICC) in the ABCD (x-axis) and HCP (y-axis) datasets. Each dot represents the univariate reliability for each dataset with the same sample size and scan time per participant (extracted from Supplementary Fig. 20b). Although the cognitive factor score is not comparable across datasets, we observed a strong correlation between the two datasets (r = 0.99).


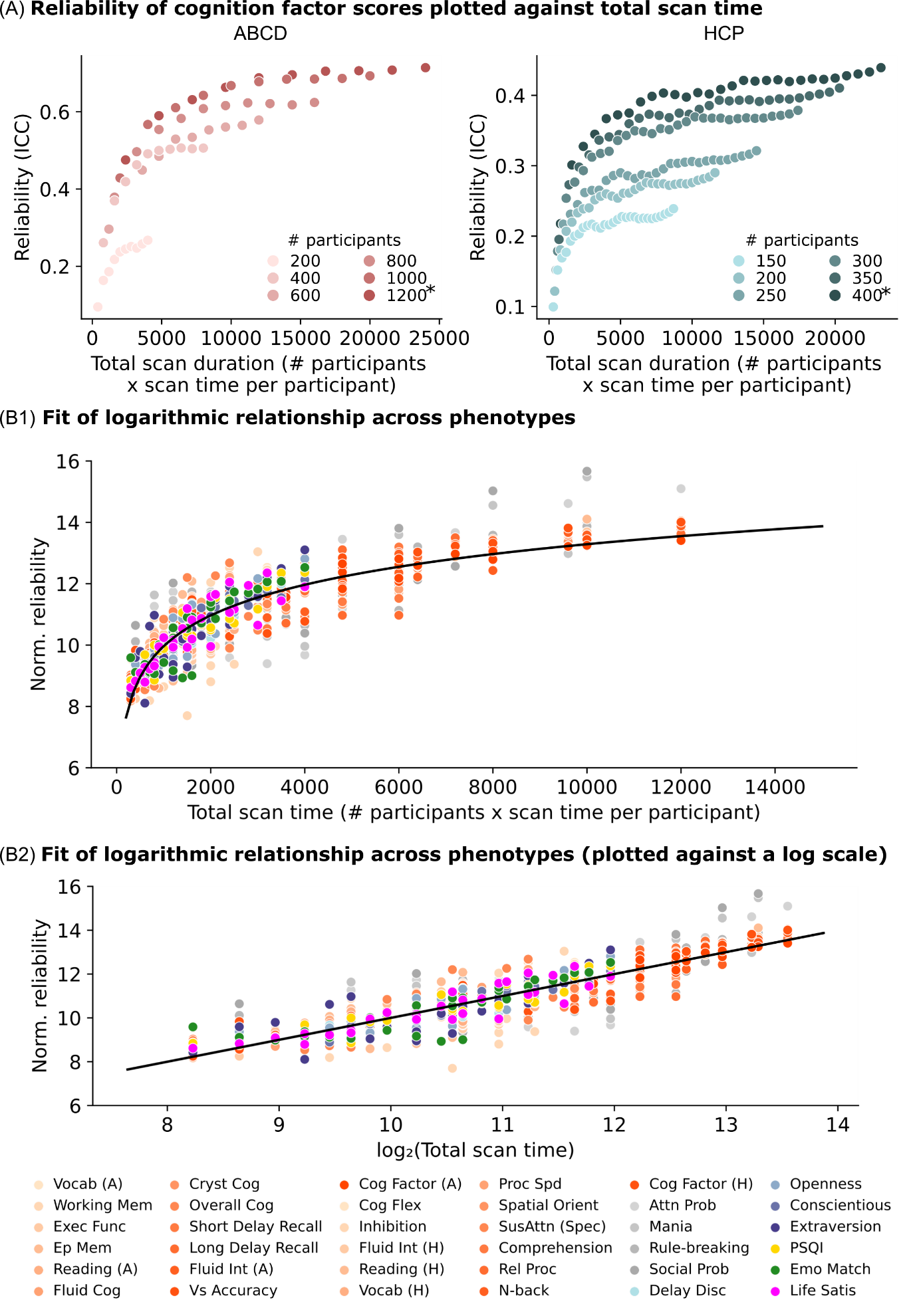


### Supplementary Fig. 22 | Logarithmic relationship to univariate BWAS.

a. Scatter plot showing reliability of univariate brain-wide association (intra-class correlation) of the cognitive factor as a function of total scan duration (defined as # training participants x scan time per participant). Each color represents a different number of total participants used to train the prediction algorithm. Plots were repeated for ABCD and HCP datasets. The * indicates that all available participants were used, therefore the sample size will be close to, but not exactly the number shown. We observed diminishing returns of scan time (relative to sample size) when scan time per participant reached approximately 10 minutes in the ABCD and HCP datasets. b1. By plotting total scan duration (number of participants × scan time per participant) against univariate BWAS reliability for each phenotype, we observed that for most phenotypes, scanning beyond 10 minutes per participant yielded diminishing marginal returns to reliability. Therefore, we performed the same logarithmic curve fitting procedure as before, but using only up to 10 minutes of scan time per participant. Scatter plot showing normalized reliability of the cognitive factor scores and 34 other phenotypes versus total scan duration ignoring data beyond 10 minutes of scan time. Blue and red dots represent results from the HCP and the ABCD datasets respectively. The logarithmic black curve suggests that total scan duration explained reliability well across phenotypic domains and datasets. b2. Same as panel b1, except the horizontal axis (total scan duration) is plotted on a logarithm scale. The linear black line suggests that the logarithm of total scan duration explained prediction performance well across phenotypic domains and datasets.

###
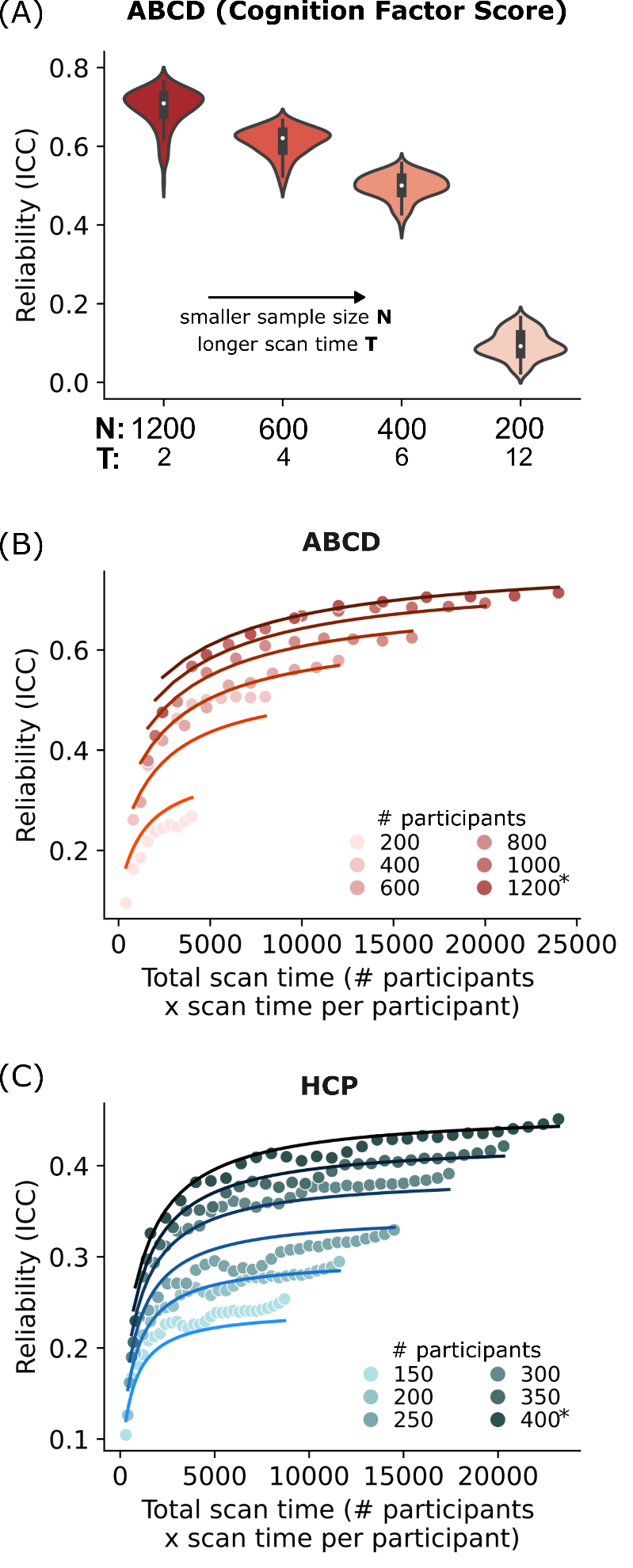
Supplementary Fig. 23 | Theoretical model fit to univariate brain-wide reliability.

Sample size and scan time are not 1-to-1 interchangeable a. Each violin shows the distribution of univariate brain-wide association analyses (BWAS) split-half reliability for the Adolescent Brain and Cognitive Development (ABCD) cognition factor score across 126 unique site combinations for a given set of scan parameters. All violins have the same total scan duration of 2400 minutes. Having a larger sample size is more beneficial for reliability than scan time. b. Scatter plot of reliability against total scan duration in the ABCD dataset. The curves were obtained by fitting a theoretical model to the reliabilities of the cognitive factor score that explains the contribution of sample size and scan time (see Supplementary Methods 1.3). We fitted the function ${Rel}_{p}=\frac{K_{0,p}}{K_{0,p}+\frac{1}{\frac{N}{2}}\left( 1-2K_{1,p}\left( \frac{1}{1+\frac{K_{2,p}}{T}} \right) \right)}$ to the data, where ${Rel}_{p}$ was the univariate split-half reliability (in terms of ICC) for phenotypic measure $p$, $N$ is the sample size and $T$ is the scan time per participant. $K_{0,p}, K_{1,p}$ and $K_{2,p}$ were estimated from data through a gradient descent, minimizing the mean squared error for each phenotypic measure. The theoretical model was able to explain why sample size is more important than scan time. c. Same as panel b but for the Human Connectome Project (HCP) dataset.


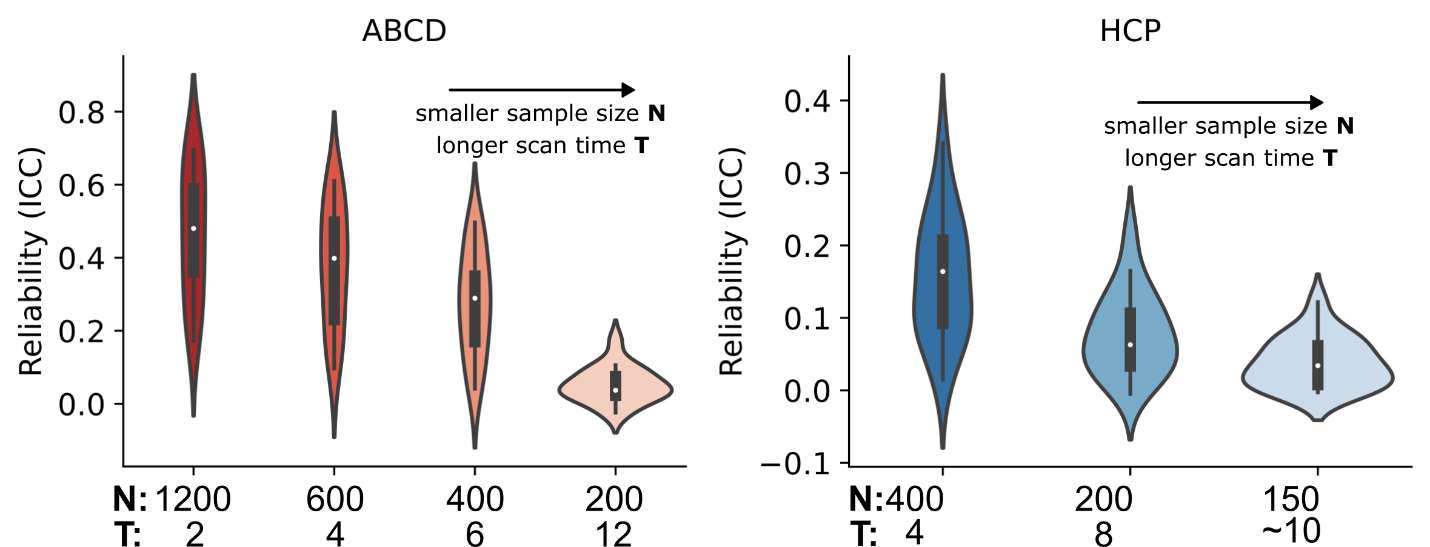


### Supplementary Fig. 24 | Interchangeability of sample size and scan time for reliability.

Same as Supplementary Fig. 23a, except in the left panel, each violin shows the distribution of average univariate brain-wide association analyses (BWAS) split-half reliability across 17 phenotypic measures in the ABCD dataset. Each violin has a total scan duration of 2400 mins (left panel). In the right panel, each violin shows the distribution average BWAS split-half reliability of the 19 HCP phenotypic measures. Each violin contains 19 data points and has a total scan duration of ~1600 mins (right panel).

Supplementary Fig. 25.1-25.3 | Reliability theoretical model fit for 17 phenotypic measures in the ABCD dataset.


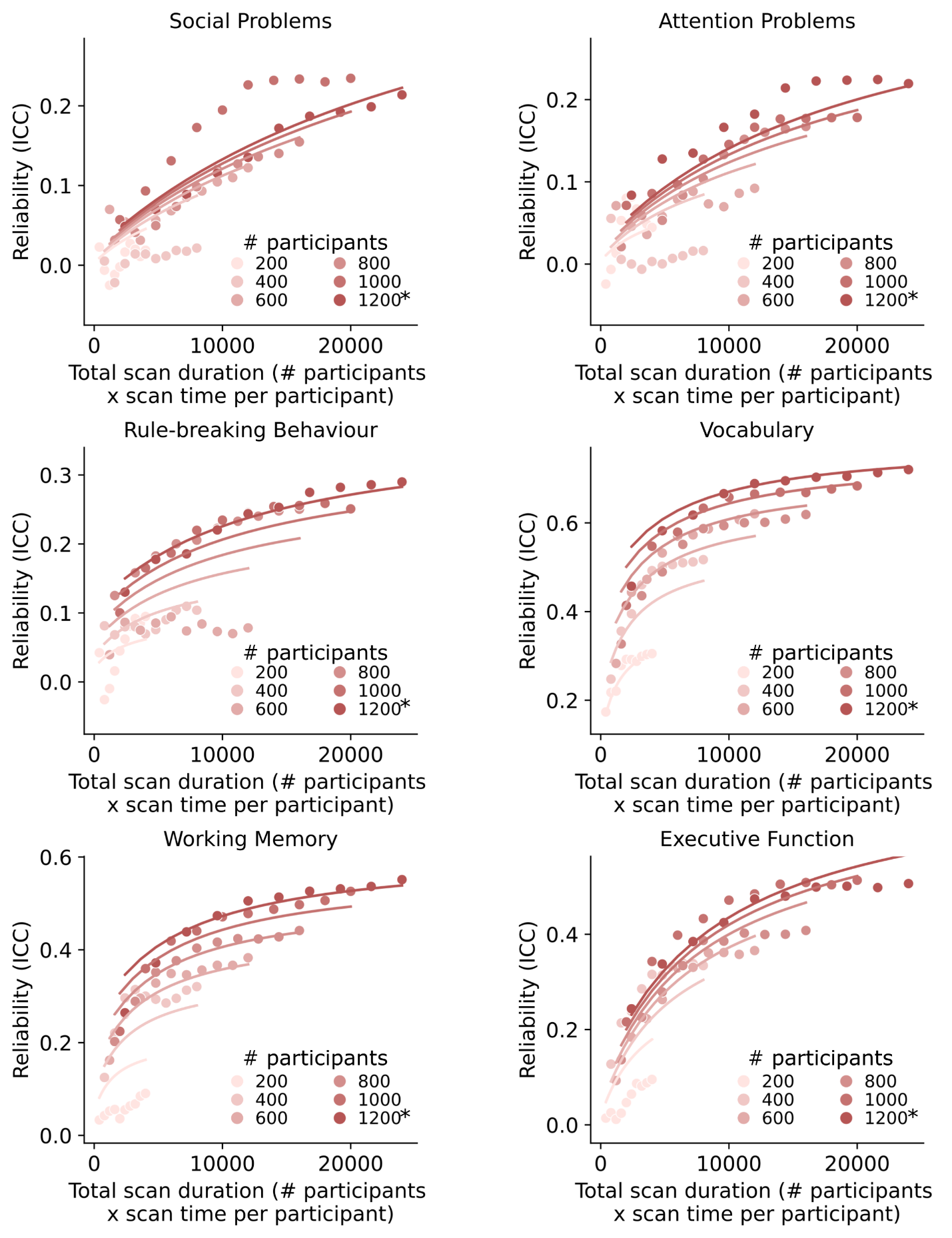
Supplementary Fig. 25.1 | Same as Supplementary Fig. 23b except showing the scatter plots and the fit of reliability theoretical model for 6 of 17 phenotypic measures in the ABCD dataset that visually follow a logarithmic pattern for prediction accuracy. Scatter plot of split-half univariate brain-wise association analyses reliability (intra-class correlation) against total scan duration in the ABCD dataset. The curves were obtained by fitting the theoretical model to the reliabilities of the phenotype. The * in the figures indicates that all available participants were used, therefore the sample size will be close to, but not exactly the number shown.


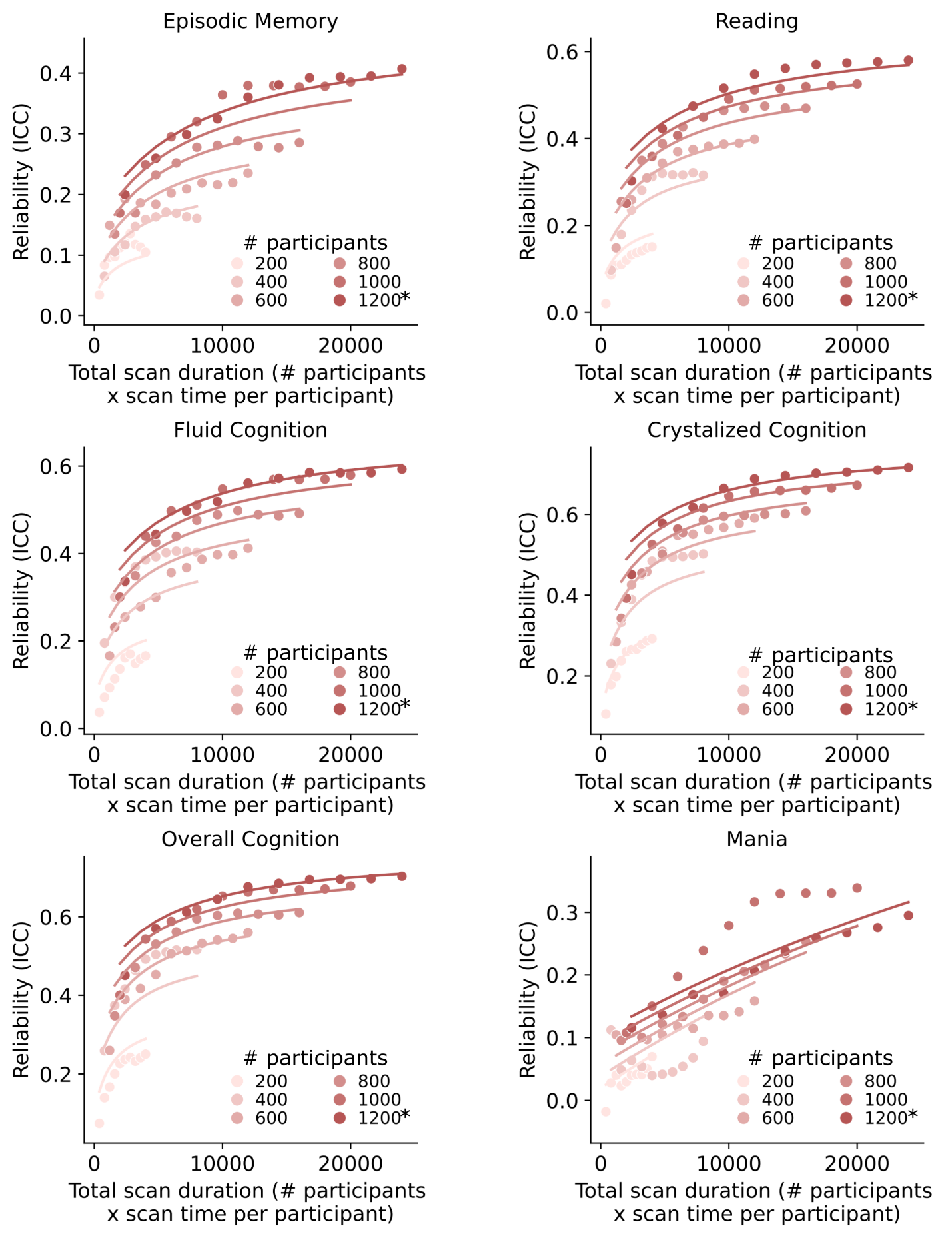


Supplementary Fig. 25.2 | Same as Supplementary Fig. 23b except showing the scatter plots and the fit of reliability theoretical model for 6 of 17 phenotypic measures in the ABCD dataset that visually follow a logarithmic pattern for prediction accuracy. Scatter plot of split-half univariate brain-wise association analyses reliability (intra-class correlation) against total scan duration in the ABCD dataset. The curves were obtained by fitting the theoretical model to the reliabilities of the phenotype. The * in the figures indicates that all available participants were used, therefore the sample size will be close to, but not exactly the number shown.


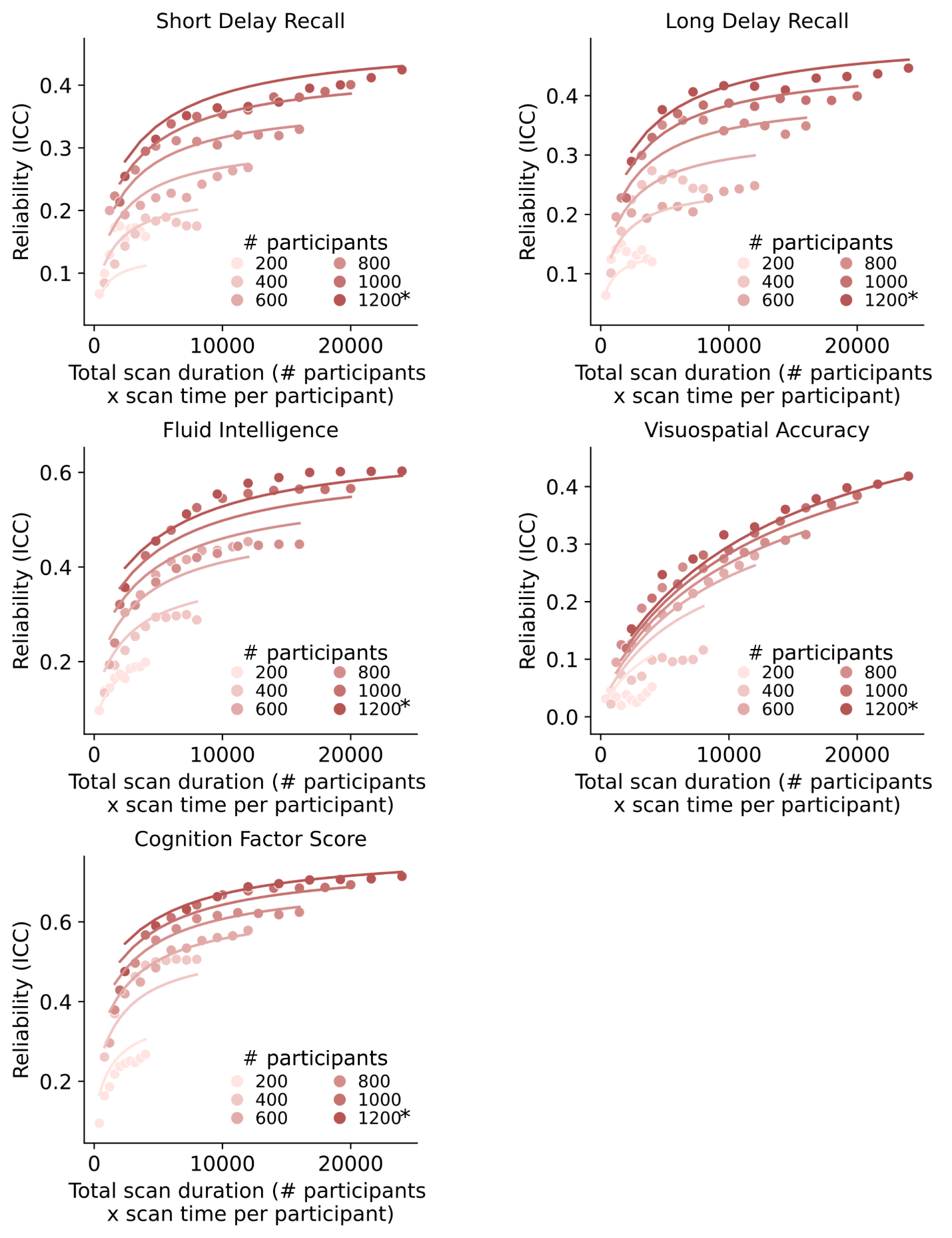


Supplementary Fig. 25.3 | Same as Supplementary Fig. 23b except showing the scatter plots and the fit of reliability theoretical model for 5 of 17 phenotypic measures in the ABCD dataset that visually follow a logarithmic pattern for prediction accuracy. Scatter plot of split-half univariate brain-wise association analyses reliability (intra-class correlation) against total scan duration in the ABCD dataset. The curves were obtained by fitting the theoretical model to the reliabilities of the phenotype. The * in the figures indicates that all available participants were used, therefore the sample size will be close to, but not exactly the number shown.

Supplementary Fig. 26.1-26.4 | Reliability theoretical model fit for 19 phenotypic measures in the HCP dataset.


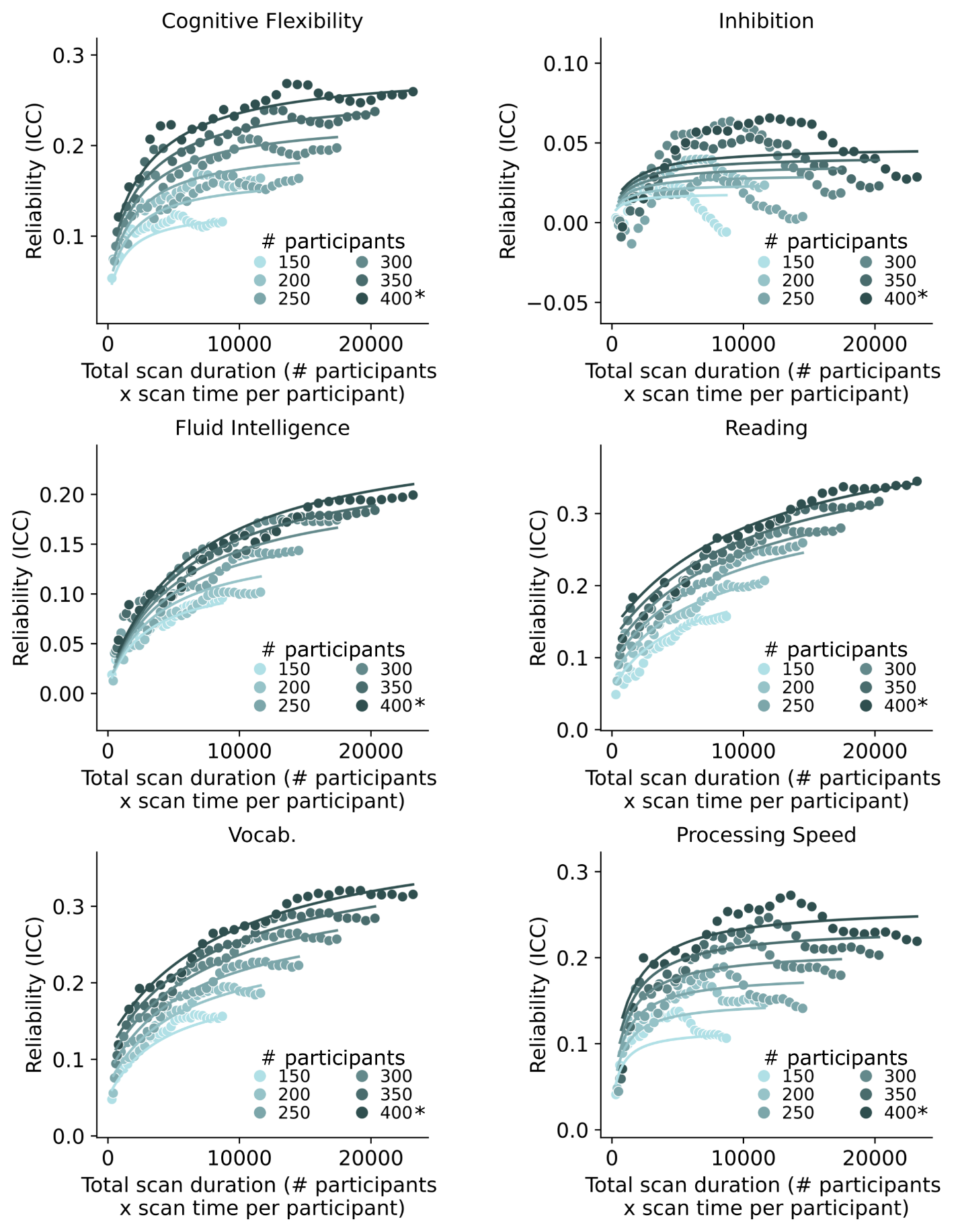
Supplementary Fig. 26.1 | Same as Supplementary Fig. 23b except showing the scatter plots and the fit of reliability theoretical model for 6 of 19 phenotypic measures in the HCP dataset that visually follow a logarithmic pattern for prediction accuracy. Scatter plot of split-half univariate brain-wise association analyses reliability (intra-class correlation) against total scan duration in the HCP dataset. The curves were obtained by fitting the theoretical model to the reliabilities of the phenotype. The * in the figures indicates that all available participants were used, therefore the sample size will be close to, but not exactly the number shown.


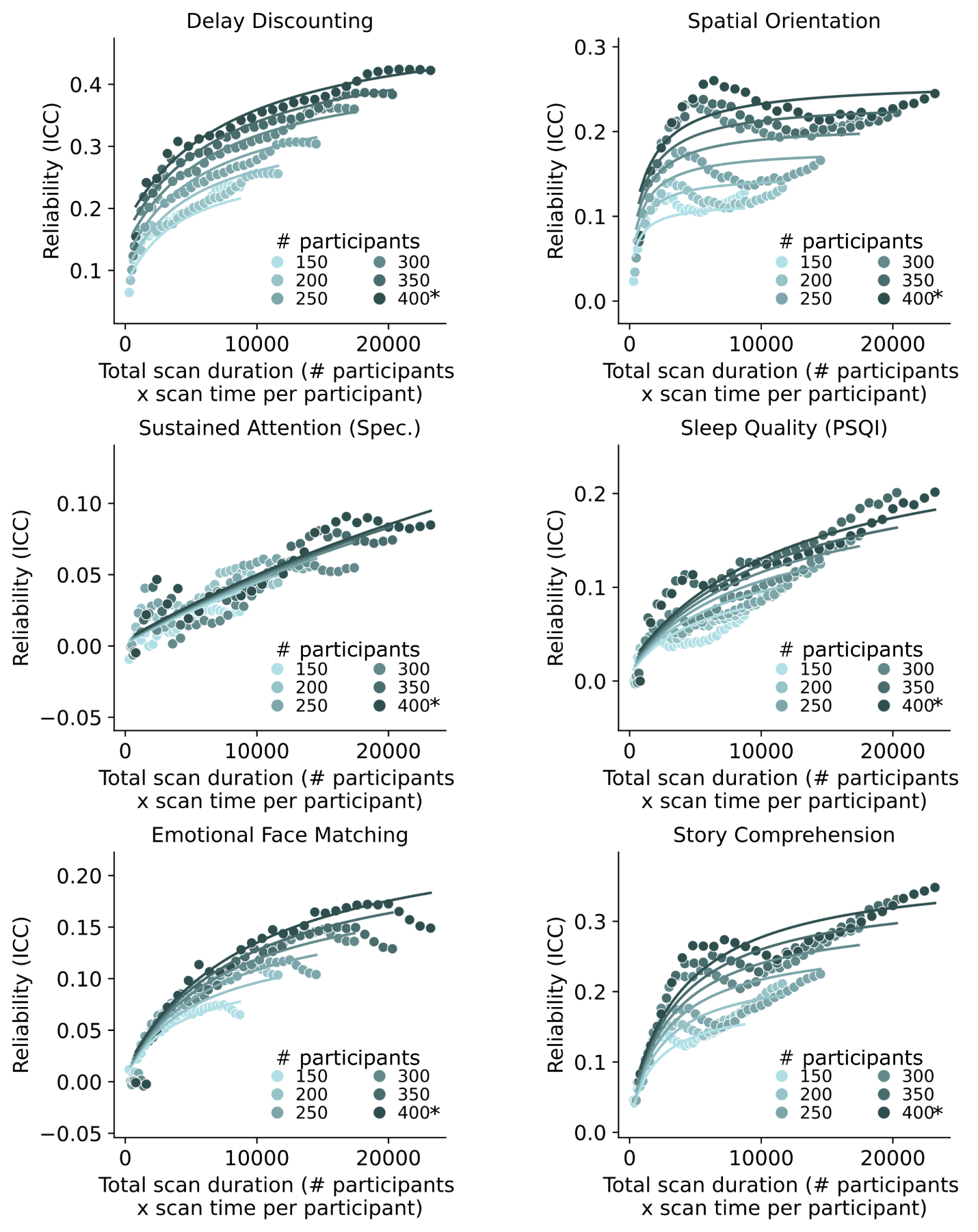
Supplementary Fig. 26.2 | Same as Supplementary Fig. 23b except showing the scatter plots and the fit of reliability theoretical model for 6 of 19 phenotypic measures in the HCP dataset that visually follow a logarithmic pattern for prediction accuracy. Scatter plot of split-half univariate brain-wise association analyses reliability (intra-class correlation) against total scan duration in the HCP dataset. The curves were obtained by fitting the theoretical model to the reliabilities of the phenotype. The * in the figures indicates that all available participants were used, therefore the sample size will be close to, but not exactly the number shown.


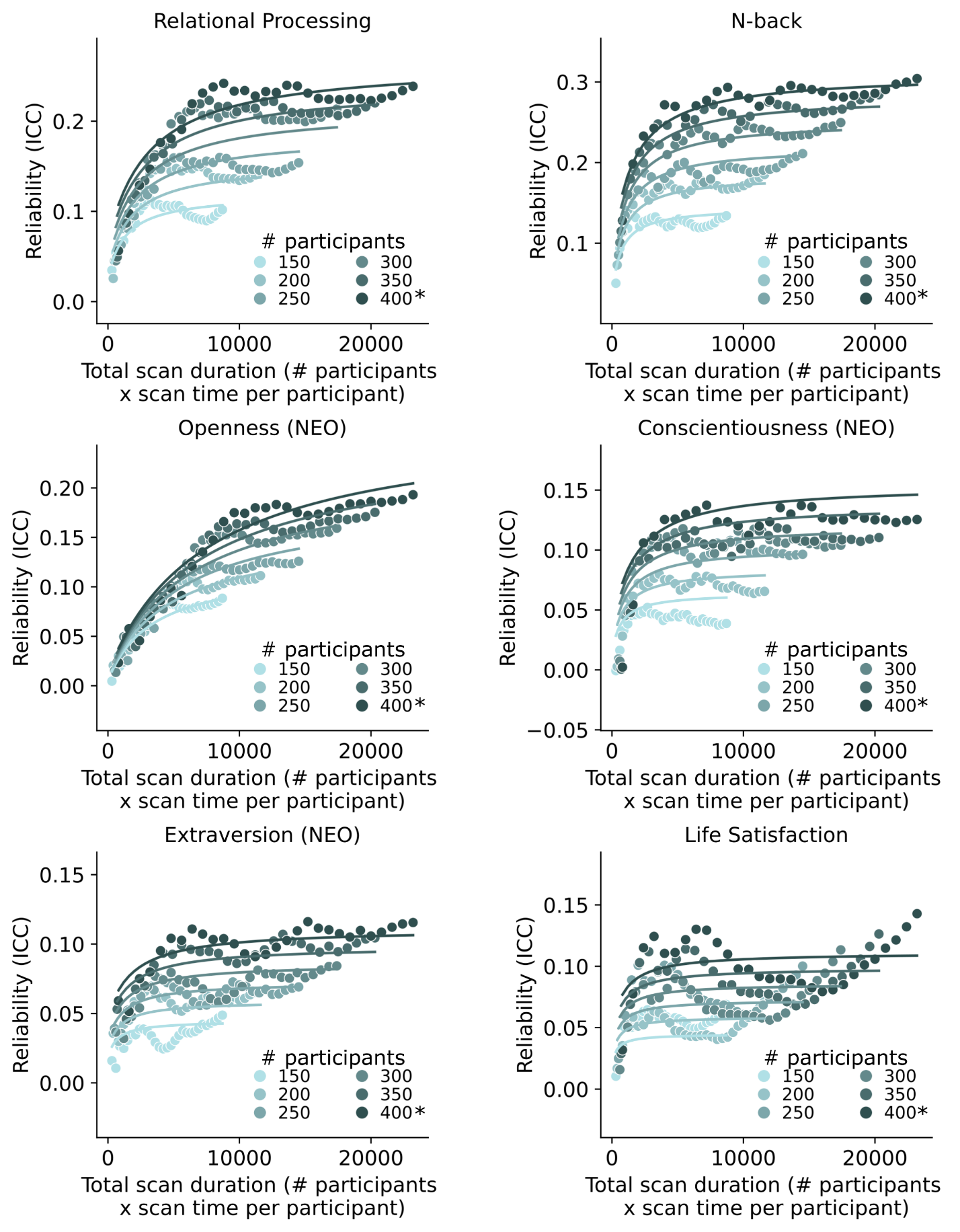


Supplementary Fig. 26.3 | Same as Supplementary Fig. 23b except showing the scatter plots and the fit of reliability theoretical model for 6 of 19 phenotypic measures in the HCP dataset that visually follow a logarithmic pattern for prediction accuracy. Scatter plot of split-half univariate brain-wise association analyses reliability (intra-class correlation) against total scan duration in the HCP dataset. The curves were obtained by fitting the theoretical model to the reliabilities of the phenotype. The * in the figures indicates that all available participants were used, therefore the sample size will be close to, but not exactly the number shown.


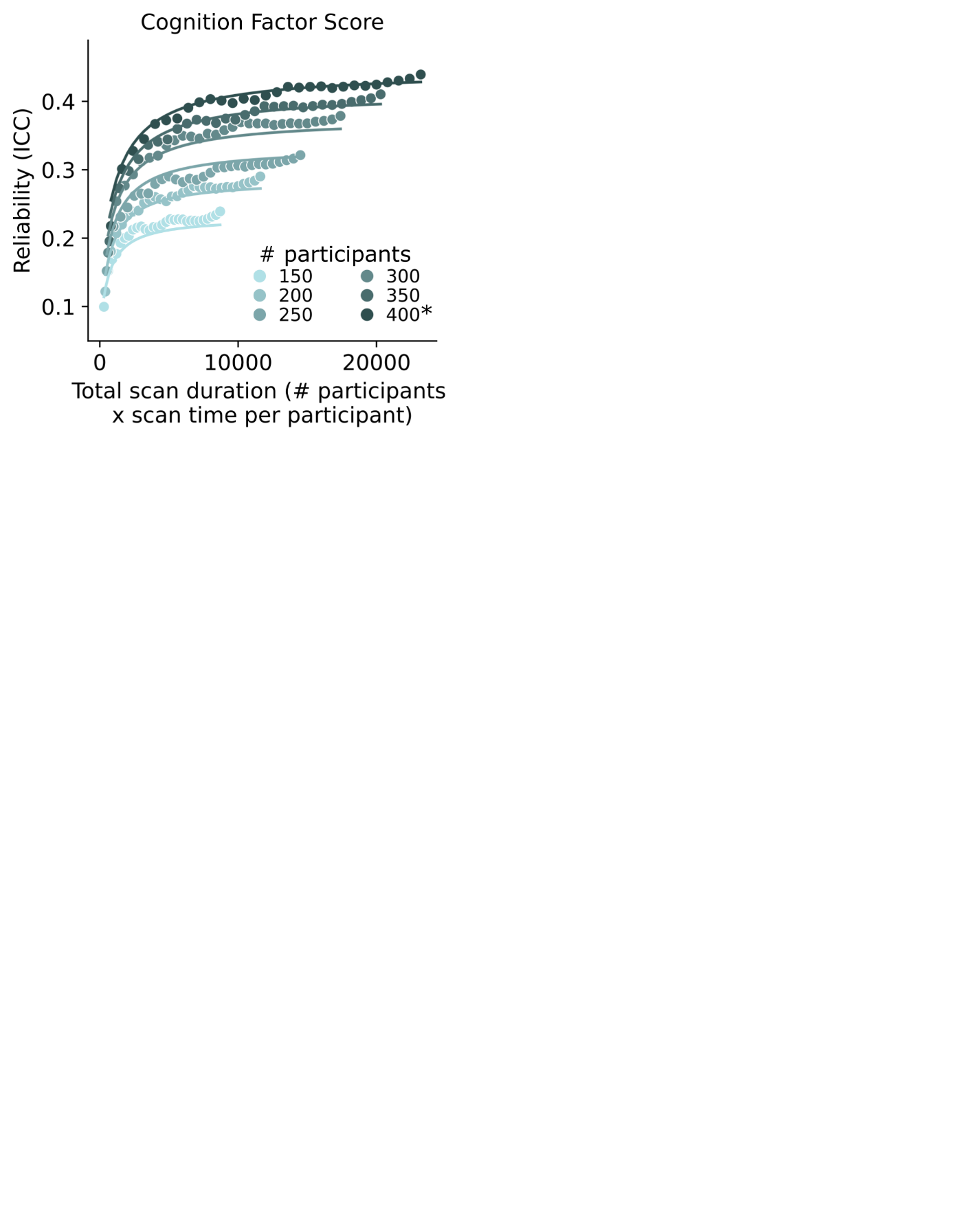


Supplementary Fig. 26.4. Same as Supplementary Fig. 23b except showing the scatter plots and the fit of reliability theoretical model for 1 of 19 phenotypic measures in the HCP dataset that visually follow a logarithmic pattern for prediction accuracy. Scatter plot of split-half univariate brain-wise association analyses reliability (intra-class correlation) against total scan duration in the HCP dataset. The curves were obtained by fitting the theoretical model to the reliabilities of the phenotype. The * in the figures indicates that all available participants were used, therefore the sample size will be close to, but not exactly the number shown.


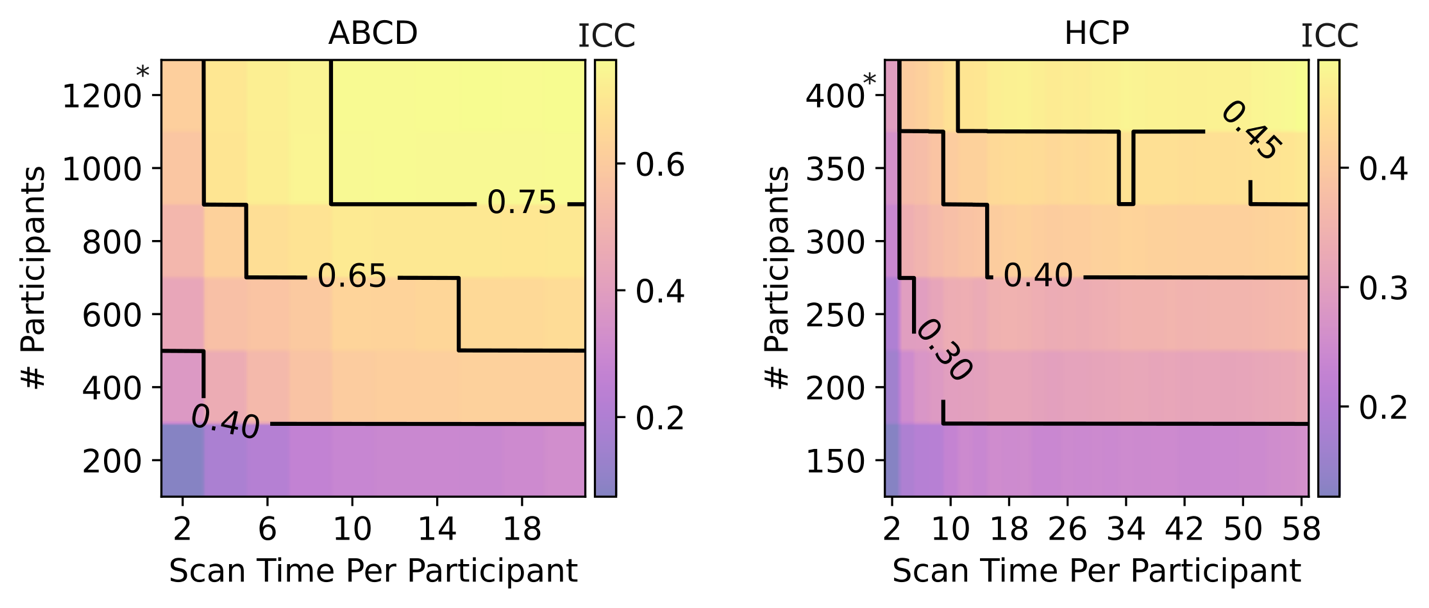


### Supplementary Fig. 27 | Contour plot of ABCD & HCP multivariate BWAS reliability.

Same as Supplementary Fig. 20b, except showing contour plot of multivariate BWAS reliability (intra-class correlation) of the cognitive factor score as a function of the scan time used to generate the functional connectivity matrix (x-axis), and the number of training participants used to train the predictive model (y-axis) in the Adolescent Brain and Cognitive Development (ABCD) and Human Connectome Project (HCP) datasets. Increasing training participants and scan time both led to increases in split-half reliability. The * in both figures indicates that all available participants were used, therefore the sample size will be close to, but not exactly the number shown.


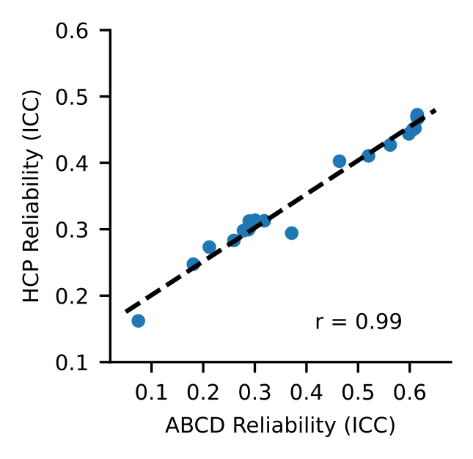


### Supplementary Fig. 28 | Correlation between multivariate BWAS reliability of ABCD and HCP cognition factor scores.

Scatter plot of the cognition factor multivariate reliability (ICC) in the ABCD (x-axis) and HCP (y-axis) datasets. Each dot represents the prediction accuracy for each dataset with the same sample size and scan time per participant (extracted from Supplementary Fig. 27). Although the cognitive factor score is not comparable across datasets, we observed a strong correlation between the two datasets (r = 0.99).


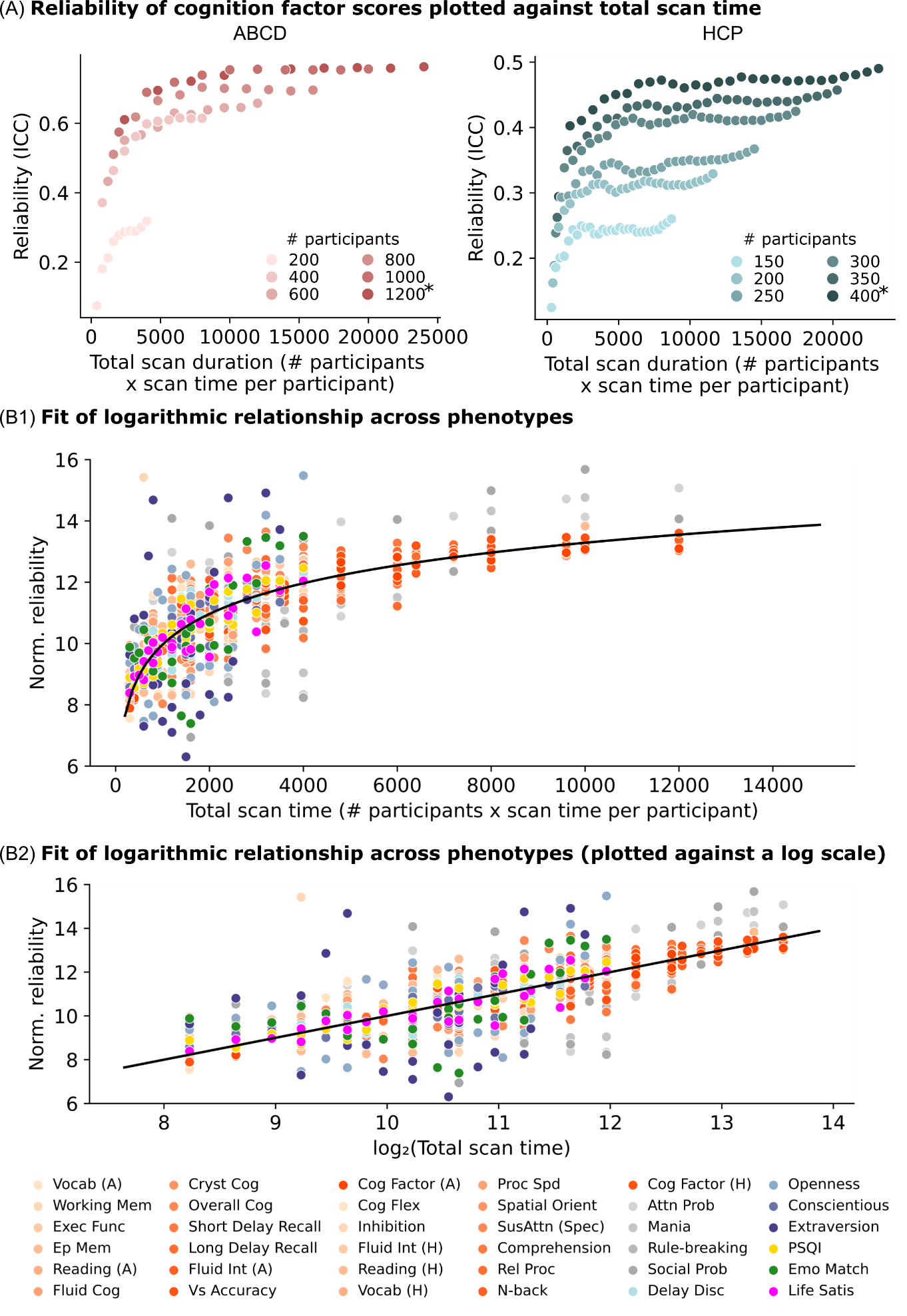


### Supplementary Fig. 29 | Logarithmic relationship to multivariate BWAS.

a. Same as Supplementary Fig. 22, except showing reliability of multivariate brain-wide association (intra-class correlation) of the cognitive factor as a function of total scan duration (defined as # participants x scan time per participant). Each color represents a different number of total participants used to train the prediction algorithm. Plots were repeated for the ABCD and HCP datasets. The * indicates that all available participants were used, therefore the sample size will be close to, but not exactly the number shown. We observed diminishing returns of scan time (relative to sample size) when scan time per participant reached approximately 10 minutes in the ABCD and HCP datasets. b1. Scatter plot showing normalized reliability of the cognitive factor scores and 34 other phenotypes versus total scan duration ignoring data beyond 10 minutes of scan time. Blue and red dots represent results from the ABCD and HCP datasets respectively. The logarithmic black curve suggests that total scan duration explained reliability well across phenotypic domains and datasets. b2. Same as panel b1, except the horizontal axis (total scan duration) is plotted on a logarithm scale. The linear black line suggests that the logarithm of total scan The linear black line suggests that the logarithm of total scan duration explained prediction performance well across phenotypic domains and datasets.

## **List of Consortium Contributors Alzheimer’s Disease Neuroimaging Initiative**

Michael Weiner³⁶, Paul Aisen³⁷, Ronald Petersen³⁸, Clifford R. Jack Jr.³⁸, William Jagust³⁹, Susan Landau³⁹, Monica Rivera-Mindt⁴⁰, Ozioma Okonkwo⁴¹, Leslie M. Shaw⁴², Edward B. Lee⁴², Arthur W. Toga⁴³, Laurel Beckett⁴⁴, Danielle Harvey⁴⁴, Robert C. Green⁴⁵, Andrew J. Saykin⁴⁶, Kwangsik Nho⁴⁶, Richard J. Perrin⁴⁷, Duygu Tosun³⁶, Pallavi Sachdev⁴⁸, Erin Drake⁴⁹, Tom Montine⁵⁰, Cat Conti⁵¹, Rachel Nosheny³⁶, Diana Truran Sacrey⁵¹, Juliet Fockler³⁶, Melanie J. Miller⁵¹, Catherine (Cat) Conti⁵¹, Winnie Kwang³⁶, Chengshi Jin³⁶, Adam Diaz⁵¹, Miriam Ashford⁵¹, Derek Flenniken⁵¹, Adrienne Kormos⁵¹, Michael Rafii³⁷, Rema Raman³⁷, Gustavo Jimenez³⁷, Michael Donohue³⁷, Jennifer Salazar³⁷, Andrea Fidell³⁷, Virginia Boatwright³⁷, Justin Robison³⁷, Caileigh Zimmerman³⁷, Yuliana Cabrera³⁷, Sarah Walter³⁷, Taylor Clanton³⁷, Elizabeth Shaffer³⁷, Caitlin Webb³⁷, Lindsey Hergesheimer³⁷, Stephanie Smith³⁷, Sheila Ogwang³⁷, Olusegun Adegoke³⁷, Payam Mahboubi³⁷, Jeremy Pizzola³⁷, Cecily Jenkins³⁷, Lisa Silbert⁵², Jeffrey Kaye⁵², Sylvia White (Salazar)⁵², Aimee Pierce⁵², Amy Thomas⁵², Tera Clay⁵², Daniel Schwartz⁵², Gillian Devereux⁵², Janet "Janae" Taylor⁵², Jennifer Ryan⁵², Mike Nguyen⁵², Madison DeCapo⁵², Yanan Shang⁵², Lon Schneider³⁷, Cynthia Munoz³⁷, Diana Ferman³⁷, Carlota Conant³⁷, Katherin Martin³⁷, Kristin Oleary³⁷, Sonia Pawluczyk³⁷, Elizabeth Trejo³⁷, Karen Dagerman³⁷, Liberty Teodoro³⁷, Mauricio Becerra³⁷, Madiha Fairooz³⁷, Sonia Garrison³⁷, Julia Boudreau³⁷, Yair Avila³⁷, James Brewer⁵⁴, Aaron Jacobson⁵⁴, Antonio Gama⁵⁴, Chi Kim⁵⁴, Emily Little⁵⁴, Jennifer Frascino⁵⁴, Nichol Ferng⁵⁴, Socorro Trujillo⁵⁴, Judith Heidebrink⁵⁵, Robert Koeppe⁵⁵, Steven MacDonald⁵⁵, Dariya Malyarenko⁵⁵, Jaimie Ziolkowski⁵⁵, James O'Connor⁵⁵, Nicole Robert⁵⁵, Suzan Lowe⁵⁵, Virginia Rogers⁵⁵, Barbara Hackenmiller³⁸, Bradley Boeve³⁸, Colleen Albers³⁸, Connie Kreuger³⁸, David Jones³⁸, David Knopman³⁸, Hugo Botha³⁸, Jessica Magnuson³⁸, Jonathan Graff-Radford³⁸, Kerry Crawley³⁸, Michael Schumacher³⁸, Sanna McKinzie³⁸, Steven Smith³⁸, Tascha Helland³⁸, Val Lowe³⁸, Vijay Ramanan³⁸, Valory Pavlik⁵⁷, Jacob Faircloth⁵⁷, Jeffrey Bishop⁵⁷, Jessica Nath⁵⁷, Maria Chaudhary⁵⁷, Maria Kataki⁵⁷, Melissa Yu⁵⁷, Nathiel Pacini⁵⁷, Randall Barker⁵⁷, Regan Brooks⁵⁷, Ruchi Aggarwal⁵⁷, Lawrence Honig⁵⁸, Yaakov Stern⁵⁸, Akiva Mintz⁵⁸, Jonathan Cordona⁵⁸, Michelle Hernandez⁵⁸, Justin Long⁴⁷, Abbey Arnold⁴⁷, Alex Groves⁴⁷, Anna Middleton⁴⁷, Blake Vogler⁴⁷, Cierra McCurry⁴⁷, Connie Mayo⁴⁷, Cyrus Raji⁴⁷, Fatima S. Amtashar⁴⁷, Heather Klemp⁴⁷, Heather Nicole Elmore⁴⁷, James Ruszkiewicz⁴⁷, Jasmina Kusuran⁴⁷, Jasmine Stewart⁴⁷, Jennifer Horenkamp⁴⁷, Julia Greeson⁴⁷, Kara Wever⁴⁷, Katie Vo⁴⁷, Kelly Larkin⁴⁷, Lesley Rao⁴⁷, Lisa Schoolcraft⁴⁷, Lora Gallagher⁴⁷, Madeline Paczynski⁴⁷, Maureen McMillan⁴⁷, Michael Holt⁴⁷, Nicole Gagliano⁴⁷, Rachel Henson⁴⁷, Renee LaBarge⁴⁷, Robert Swarm⁴⁷, Sarah Munie⁴⁷, Serena Cepeda⁴⁷, Stacey Winterton⁴⁷, Stephen Hegedus⁴⁷, TaNisha Wilson⁴⁷, Tanya Harte⁴⁷, Zach Bonacorsi⁴⁷, David Geldmacher⁶⁰, Amber Watkins⁶⁰, Brandi Barger⁶⁰, Bryan Smelser⁶⁰, Charna Bates⁶⁰, Cynthia Stover⁶⁰, Emily McKinley⁶⁰, Gregory Ikner⁶⁰, Haley Hendrix⁶⁰, Harold Matthew Cooper⁶⁰, Jennifer Mahaffey⁶⁰, Lindsey Booth Robbins⁶⁰, Loren Brown Ashley⁶⁰, Marissa Natelson-Love⁶⁰, Princess Carter⁶⁰, Veronika Solomon⁶⁰, Hillel Grossman⁶¹, Alexandra Groome⁶¹, Allison Ardolino⁶¹, Anthony Kaplan⁶¹, Faye Sheppard⁶¹, Genesis Burgos-Rivera⁶¹, Gina Garcia-Camilo⁶¹, Joanne Lim⁶¹, Judith Neugroschl⁶¹, Kimberly Jackson⁶¹, Kirsten Evans⁶¹, Laili Soleimani⁶¹, Mary Sano⁶¹, Nasrin Ghesani⁶¹, Sarah Binder⁶¹, Xiomara Mendoza Apuango⁶¹, Ajay Sood⁶², Amelia Troutman⁶², Kimberly Blanchard⁶², Arlene Richards⁶², Grace Nelson⁶², Kirsten Hendrickson⁶², Erin Yurko⁶², Jamie Plenge⁶², Victoria Rufo⁶², Raj Shah⁶², Ranjan Duara⁶³, Brendan Lynch⁶³, Cesar Chirinos⁶³, Christine Dittrich⁶³, Debbie Campbell⁶³, Diego Mejia⁶³, Gilberto Perez⁶³, Helena Colvee⁶³, Joanna Gonzalez⁶³, Josalen Gondrez⁶³, Joshua Knaack⁶³, Mara Acevedo⁶³, Maria Cereijo⁶³, Maria Greig-Custo⁶³, Michelle Villar⁶³, Morris Wishnia⁶³, Sheryl Detling⁶³, Warren Barker⁶³, Marilyn Albert⁶⁴, Abhay Moghekar⁶⁴, Barbara Rodzon⁶⁴, Corey Demsky⁶⁴, Gregory Pontone⁶⁴, Jim Pekar⁶⁴, Leonie Farrington⁶⁴, Martin Pomper⁶⁴, Nicole Johnson⁶⁴, Tolulope Alo⁶⁴, Martin Sadowski⁶⁵, Anaztasia Ulysse⁶⁵, Arjun Masurkar⁶⁵, Brittany Marti⁶⁵, David Mossa⁶⁵, Emilie Geesey⁶⁵, Emily Petrocca⁶⁵, Evan Schulze⁶⁵, Jennifer Wong⁶⁵, Joseph Boonsiri⁶⁵, Sunnie Kenowsky⁶⁵, Tatianne Martinez⁶⁵, Veronica Briglall⁶⁵, P. Murali Doraiswamy⁶⁶, Adaora Nwosu⁶⁶, Alisa Adhikari⁶⁶, Cammie Hellegers⁶⁶, Jeffrey Petrella⁶⁶, Olga James⁶⁶, Terence Wong⁶⁶, Thomas Hawk⁶⁶, Sanjeev Vaishnavi⁴², Hannah McCoubrey⁴², Ilya Nasrallah⁴², Rachel Rovere⁴², Jeffrey Maneval⁴², Elizabeth Robinson⁴², Francisco Rivera⁴², Jade Uffelman⁴², Martha Combs⁴², Patricia O'Donnell⁴², Sara Manning⁴², Richard King⁶⁸, Alayne Nieto⁶⁸, Amanda Glueck⁶⁸, Anjana Mandal⁶⁸, Audrie Swain⁶⁸, Bethanie Gamble⁶⁸, Beverly Meacham⁶⁸, Denece Forenback⁶⁸, Dorothy Ross⁶⁸, Elizabeth Cheatham⁶⁸, Ellen Hartman⁶⁸, Gary Cornell⁶⁸, Jordan Harp⁶⁸, Laura Ashe⁶⁸, Laura Goins⁶⁸, Linda Watts⁶⁸, Morgan Yazell⁶⁸, Prabin Mandal⁶⁸, Regan Buckler⁶⁸, Sylvia Vincent⁶⁸, Triana Rudd⁶⁸, Oscar Lopez⁶⁹, Ann Arlene Malia⁶⁹, Caitlin Chiado⁶⁹, Cary Zik⁶⁹, James Ruszkiewicz⁴⁷, Kathleen Savage⁶⁹, Linda Fenice⁶⁹, MaryAnn Oakley⁶⁹, Paige C Tacey⁶⁹, Sarah Berman⁶⁹, Sarah Bowser⁶⁹, Stephen Hegedus⁴⁷, Xanthia Saganis⁶⁹, Anton Porsteinsson⁷⁰, Abigail Mathewson⁷⁰, Asa Widman⁷⁰, Bridget Holvey⁷⁰, Emily Clark⁷⁰, Esmeralda Morales⁷⁰, Iris Young⁷⁰, Kevin Hopkins⁷⁰, Kimberly Martin⁷⁰, Nancy Kowalski⁷⁰, Rebecca Hunt⁷⁰, Roberta Calzavara⁷⁰, Russell Kurvach⁷⁰, Stephen D'Ambrosio⁷⁰, Gaby Thai⁷¹, Beatriz Vides⁷¹, Brigit Lieb⁷¹, Catherine McAdams-Ortiz⁷¹, Cyndy Toso⁷¹, Ivan Mares⁷¹, Kathryn Moorlach⁷¹, Luter Liu⁷¹, Maria Corona⁷¹, Mary Nguyen⁷¹, Melanie Tallakson⁷¹, Michelle McDonnell⁷¹, Milagros Rangel⁷¹, Neetha Basheer⁷¹, Patricia Place⁷¹, Romina Romero⁷¹, Steven Tam⁷¹, Trung Nguyen⁷², Abey Thomas⁷², Alexander (Alex) Frolov⁷², Alka Khera⁷², Amy Browning⁷², Brendan Kelley⁷², Courtney Dawson⁷², Dana Mathews⁷², Elaine Most⁷², Elizeva (Ellie) Phillips⁷², Lynn Nguyen⁷², Maribel Nunez⁷², Matalin Miller⁷², Matthew R. Jones⁷², Natalie Martinez⁷², Rebecca Logan⁷², Roderick McColl⁷², Sari Pham⁷², Tiffani Fox⁷², Tracey Moore⁷², Allan Levey⁷³, Abby Brown⁷³, Andrea Kippels⁷³, Ashton Ellison⁷³, Casie Lyons⁷³, Chadwick Hales⁷³, Cindy Parry⁷³, Courtney Williams⁷³, Elizabeth McCorkle⁷³, Guy Harris⁷³, Heather Rose⁷³, Inara Jooma⁷³, Jahmila Al-Amin⁷³, James Lah⁷³, James Webster⁷³, Jessica Swiniarski⁷³, Latasha Chapman⁷³, Laura Donnelly⁷³, Lauren Mariotti⁷³, Mary Locke⁷³, Phyllis Vaughn⁷³, Rachael Penn⁷³, Sallie Carpentier⁷³, Samira Yeboah⁷³, Sarah Basadre⁷³, Sarah Malakauskas⁷³, Stefka Lyron⁷³, Tara Villinger⁷³, Terra Burney⁷³, Jeffrey Burns⁷⁴, Ala Abusalim⁷⁴, Alexandra Dahlgren⁷⁴, Alexandria Montero⁷⁴, Anne Arthur⁷⁴, Heather Dooly⁷⁴, Katelynn Kreszyn⁷⁴, Katherine Berner⁷⁴, Lindsey Gillen⁷⁴, Maria Scanlan⁷⁴, Mercedes Madison⁷⁴, Nicole Mathis⁷⁴, Phyllis Switzer⁷⁴, Ryan Townley⁷⁴, Samantha Fikru⁷⁴, Samantha Sullivan⁷⁴, Ella Wright⁷⁴, Maryam Beigi⁴³, Anthony Daley⁴³, Ashley Ko⁴³, Brittney Luong⁴³, Glen Nyborg⁴³, Jessica Morales⁴³, Kelly Durbin⁴³, Lauren Garcia⁴³, Leila Parand⁴³, Lorena Macias⁴³, Lorena Monserratt⁴³, Maya Farchi⁴³, Pauline Wu⁴³, Robert Hernandez⁴³, Thao Rodriguez⁴³, Neill Graff-Radford⁷⁶, Allana Marolt⁷⁶, Anton Thomas⁷⁶, Deborah Aloszka⁷⁶, Ereilia Moncayo⁷⁶, Erin Westerhold⁷⁶, Gregory Day⁷⁶, Kandise Chrestensen⁷⁶, Mary Imhansiemhonchi⁷⁶, Sanna McKinzie³⁸, Sochenda Stephens⁷⁶, Sylvia Grant⁷⁶, Jared Brosch⁴⁶, Amy Perkins⁴⁶, Aubree Saunders⁴⁶, Debra Silberberg Kovac⁴⁶, Heather Polson⁴⁶, Isabell Mwaura⁴⁶, Kassandra Mejia⁴⁶, Katherine Britt⁴⁶, Kathy King⁴⁶, Kayla Nichols⁴⁶, Kayley Lawrence⁴⁶, Lisa Rankin⁴⁶, Martin Farlow⁴⁶, Patricia Wiesenauer⁴⁶, Robert Bryant⁴⁶, Scott Herring⁴⁶, Sheryl Lynch⁴⁶, Skylar Wilson⁴⁶, Traci Day⁴⁶, William Korst⁴⁶, Christopher van Dyck⁷⁸, Adam Mecca⁷⁸, Alyssa Miller⁷⁸, Amanda Brennan⁷⁸, Amber Khan⁷⁸, Audrey Ruan⁷⁸, Carol Gunnoud⁷⁸, Chelsea Mendonca⁷⁸, Danielle Raynes-Goldfinger⁷⁸, Elaheh Salardini⁷⁸, Elisa Hidalgo⁷⁸, Emma Cooper⁷⁸, Erawadi Singh⁷⁸, Erin Murphy⁷⁸, Jeanine May⁷⁸, Jesse Stanhope⁷⁸, Jessica Lam⁷⁸, Julia Waszak⁷⁸, Kimberly Nelsen⁷⁸, Kimberly Sacaza⁷⁸, Mayer Joshua Hasbani⁷⁸, Meghan Donahue⁷⁸, Ming-Kai Chen⁷⁸, Nicole Barcelos⁷⁸, Paul Eigenberger⁷⁸, Robin Bonomi⁷⁸, Ryan O'Dell⁷⁸, Sarah Jefferson⁷⁸, Siddharth Khasnavis⁷⁸, Stephen Smilowitz⁷⁸, Susan DeStefano⁷⁸, Susan Good⁷⁸, Terry Camarro⁷⁸, Vanessa Clayton⁷⁸, Yanis Cayrel⁷⁸, YuQuan "Oliver" Lu⁷⁸, Howard Chertkow⁷⁹, Howard Bergman⁷⁹, Chris Hosein⁷⁹, Sandra Black⁸⁰, Anish Kapadia⁸⁰, Aparna Bhan⁸⁰, Benjamin Lam⁸⁰, Christopher Scott⁸⁰, Gillian Gabriel⁸⁰, Jennifer Bray⁸⁰, Ljubica Zotovic⁸⁰, Maria Samira Gutierrez⁸⁰, Mario Masellis⁸⁰, Marjan Farshadi⁸⁰, Maurylette Gui⁸⁰, Meghan Mitchell⁸⁰, Rebecca Taylor⁸⁰, Ruby Endre⁸⁰, Zhala Taghi-Zada⁸⁰, Robin Hsiung⁸¹, Carolyn English⁸¹, Ellen Kim⁸¹, Eugene Yau⁸¹, Haley Tong⁸¹, Laura Barlow⁸¹, Lauren Jennings⁸¹, Michele Assaly⁸¹, Paula Nunes⁸¹, Tahlee Marian⁸¹, Andrew Kertesz⁸², John Rogers⁸², Dick Trost⁸², Dylan Wint⁸³, Charles Bernick⁸³, Donna Munic⁸³, Ian Grant⁸⁴, Aaliyah Korkoyah⁸⁴, Ali Raja⁸⁴, Allison Lapins⁸⁴, Caila Ryan⁸⁴, Jelena Pejic⁸⁴, Kailey Basham⁸⁴, Leena Lukose⁸⁴, Loreece Haddad⁸⁴, Lucas Quinlan⁸⁴, Nathaniel Houghtaling⁸⁴, Carl Sadowsky⁸⁵, Walter Martinez⁸⁵, Teresa Villena⁸⁵, Brigid Reynolds⁸⁶, Angelica Forero⁸⁶, Carolyn Ward⁸⁶, Emma Brennan⁸⁶, Esteban Figueroa⁸⁶, Giuseppe Esposito⁸⁶, Jessica Mallory⁸⁶, Kathleen Johnson⁸⁶, Kathryn Turner⁸⁶, Katie Seidenberg⁸⁶, Kelly McCann⁸⁶, Margaret Bassett⁸⁶, Melanie Chadwick⁸⁶, Raymond Scott Turner⁸⁶, Robin Bean⁸⁶, Saurabh Sharma⁸⁶, Gad Marshall⁸⁷, Aferdita Haviari⁸⁷, Alison Pietras⁸⁷, Bradley Wallace⁸⁷, Catherine Munro⁸⁷, Gladiliz Rivera-Delpin⁸⁷, Hadley Hustead⁸⁷, Isabella Levesque⁸⁷, Jennifer Ramirez⁸⁷, Karen Nolan⁸⁷, Kirsten Glennon⁸⁷, Mariana Palou⁸⁷, Michael Erkkinen⁸⁷, Nicole DaSilva⁸⁷, Pamela Friedman⁸⁷, Regina M. Silver⁸⁷, Ricardo Salazar⁸⁷, Roxxanne Polleys⁸⁷, Scott McGinnis⁸⁷, Seth Gale⁸⁷, Tia Hall⁸⁷, Tuan Luu⁸⁷, Steven Chao⁸⁸, Emmeline Lin⁸⁸, Jaila Coleman⁸⁸, Kevin Epperson⁸⁸, Minal Vasanawala⁸⁸, Alireza Atri⁸⁹, Amy Rangel⁸⁹, Brittani Evans⁸⁹, Candy Monarrez⁸⁹, Carol Cline⁸⁹, Carolyn Liebsack⁸⁹, Daniel Bandy⁸⁹, Danielle Goldfarb⁸⁹, Debbie Intorcia⁸⁹, Jennifer Olgin⁸⁹, Kelly Clark⁸⁹, Kelsey King⁸⁹, Kylee York⁸⁹, Marina Reade⁸⁹, Michael Callan⁸⁹, Michael Glass⁸⁹, Michaela Johnson⁸⁹, Michele Gutierrez⁸⁹, Molly Goddard⁸⁹, Nadira Tmcic⁸⁹, Parichita Choudhury⁸⁹, Priscilla Reyes⁸⁹, Serena Lowery⁸⁹, Shaundra Hall⁸⁹, Sonia Olgin⁸⁹, Stephanie de Santiago⁸⁹, Michael Alosco⁴⁵, Alyssa Ton⁴⁵, Amanda Jimenez⁴⁵, Andrew Ellison⁴⁵, Anh Tran⁴⁵, Brandon Anderson⁴⁵, Della Carter⁴⁵, Donna Veronelli⁴⁵, Steven Lenio⁴⁵, Eric Steinberg⁴⁵, Jesse Mez⁴⁵, Jason Weller⁴⁵, Jennifer Johns⁴⁵, Jessica Harkins⁴⁵, Alexa Pulcio⁴⁵, Ina Hoti⁴⁵, Jane Mwicigi⁴⁵, Mona Lauture⁴⁵, Ridiane Denis⁴⁵, Ronald Killiany⁴⁵, Sarah Singh⁴⁵, Wendy Qiu⁴⁵, Year Devis⁴⁵, Thomas Obisesan⁹¹, Andrew Stone⁹¹, Debra Ordor⁹¹, Ifreke Udodong⁹¹, Immaculata Okonkwo⁹¹, Javed Khan⁹¹, Jillian Turner⁹¹, Kyliah Hughes⁹¹, Oshoze Kadiri⁹¹, Charles Duffy⁹², Ariana Moss⁹², Katherine Stapleton⁹², Maria Toth⁹², Marianne Sanders⁹², Martin Ayres⁹², Melissa Hamski⁹², Parianne Fatica⁹², Paula Ogrocki⁹², Sarah Ash⁹², Stacy Pot⁹², Doris Chen⁴⁴, Andres Soto⁴⁴, Costin Tanase⁴⁴, David Bissig⁴⁴, Hafsanoor Vanya⁴⁴, Heather Russell⁴⁴, Hitesh Patel⁴⁴, Hongzheng Zhang⁴⁴, Kelly Wallace⁴⁴, Kristi Ayers⁴⁴, Maria Gallegos⁴⁴, Martha Forloines⁴⁴, Meghan Sim⁴⁴, Queennie Majorie S Kahulugan⁴⁴, Richard Isip⁴⁴, Sandra Calderon⁴⁴, Talia Hamm⁴⁴, Michael Borrie⁹⁴, T-Y Lee⁹⁴, Rob Bartha⁹⁴, Sterling Johnson⁴¹, Sanjay Asthana⁴¹, Cynthia M. Carlsson⁴¹, Allison Perrin⁹⁶, Pierre Tariot⁹⁶, Adam Fleisher⁹⁶, Stephanie Reeder⁹⁶, Horacio Capote⁹⁷, Allison Emborsky⁹⁷, Anna Mattle⁹⁷, Bela Ajtai⁹⁷, Benjamin Wagner⁹⁷, Bennett Myers⁹⁷, Daryn Slazyk⁹⁷, Delaney Fragale⁹⁷, Erin Fransen⁹⁷, Heather Macnamara⁹⁷, Jonathan Falletta⁹⁷, Joseph Hirtreiter⁹⁷, Laszlo Mechtler⁹⁷, Megan King⁹⁷, Michael Asbach⁹⁷, Michelle Rainka⁹⁷, Richard Zawislak⁹⁷, Scott Wisniewski⁹⁷, Stephanie O'Malley⁹⁷, Tatiana Jimenez-Knight⁹⁷, Todd Peehler⁹⁷, Traci Aladeen⁹⁷, Vernice Bates⁹⁷, Violet Wenner⁹⁷, Wisam Elmalik⁹⁷, Douglas W. Scharre⁹⁸, Arun Ramamurthy⁹⁸, Soumya Bouchachi⁹⁸, Maria Kataki⁵⁷, Rawan Tarawneh⁹⁸, Brendan Kelley⁷², Dzintra Celmins⁹⁹, Alicia Leader⁹⁹, Chris Figueroa⁹⁹, Heather Bauerle⁹⁹, Katlynn Patterson⁹⁹, Michael Reposa⁹⁹, Steven Presto⁹⁹, Tuba Ahmed⁹⁹, Wendy Stewart⁹⁹, Godfrey D. Pearlson¹⁰⁰, Karen Blank¹⁰⁰, Karen Anderson¹⁰⁰, Robert B. Santulli¹⁰¹, Eben S. Schwartz¹⁰¹, Jeff Williamson¹⁰², Alicia Jessup¹⁰², Andrea Williams¹⁰², Crystal Duncan¹⁰², Abigail O'Connell¹⁰², Karen Gagnon¹⁰², Ezequiel Zamora¹⁰², James Bateman¹⁰², Freda Crawford¹⁰², Deb Thompson¹⁰², Eboni Walker¹⁰², Jennifer Rowell¹⁰², Mikell White¹⁰², Susan Henkle¹⁰², Joseph Bottoms¹⁰², Lena Moretz¹⁰², Bevan Hoover¹⁰², Michael Shannon¹⁰², Samantha Rogers¹⁰², Wendy Baker¹⁰², William Harrison¹⁰², Chuang-Kuo Wu¹⁰³, Alexis DeMarco¹⁰³, Ava Stipanovich¹⁰³, Daniel Arcuri¹⁰³, Jan Clark¹⁰³, Jennifer Davis¹⁰³, Kerstin Doyon¹⁰³, Marie Amoyaw¹⁰³, Mauro Veras Acosta¹⁰³, Ronald Bailey¹⁰³, Scott Warren¹⁰³, Terry Fogerty¹⁰³, Victoria Sanborn¹⁰³, Meghan Riddle¹⁰⁴, Stephen Salloway¹⁰⁴, Paul Malloy¹⁰⁴, Stephen Correia¹⁰⁴, Charles Window³⁶, Morgan Blackburn³⁶, Howard J. Rosen³⁶, Bruce L. Miller³⁶, Amanda Smith¹⁰⁶, Ijeoma Mba¹⁰⁶, Jenny Echevarria¹⁰⁶, Juris Janavs¹⁰⁶, Emily Roglaski¹⁰⁷, Meagan Yong¹⁰⁷, Rebecca Devine¹⁰⁷, Hamid Okhravi¹⁰⁸, Edgardo Rivera¹⁰⁹, Teresa Kalowsky¹⁰⁹, Caroline Smith¹⁰⁹, Christina Rosario¹⁰⁹, Joseph Masdeu¹¹⁰, Richard Le¹¹⁰, Maushami Gurung¹¹⁰, Marwan Sabbagh¹¹¹, Angelica Garcia¹¹¹, Micah Ellis Slaughter¹¹¹, Nadeen Elayan¹¹¹, Skieff Acothley¹¹¹, Nunzio Pomara¹¹², Raymundo Hernando¹¹², Vita Pomara¹¹², Chelsea Reichert¹¹², Olga Brawman-Mintzer¹¹³, Allison Acree¹¹³, Arthur Williams¹¹³, Campbell Long¹¹³, Rebecca Long¹¹³, Paul Newhouse¹¹⁴, Sydni Jene Hill¹¹⁴, Amy Boegel¹¹⁴, Sudha Seshadri¹¹⁵, Amy Saklad¹¹⁵, Floyd Jones¹¹⁵, William Hu¹¹⁶, V. Sotelo¹¹⁶, Yaneicy Gonzalez Rojas¹¹⁷, Jacobo Mintzer¹¹⁸, Crystal Flynn Longmire¹¹⁸, Kenneth Spicer¹¹⁸

## **Author affiliations for the Alzheimer’s Disease Neuroimaging Initiative**

³⁶University of California, San Francisco, ³⁷University of Southern California, ³⁸Mayo Clinic, Rochester, ³⁹University of California, Berkeley, ⁴⁰Fordham University; Mt. Sinai Medical Center, ⁴¹University of Wisconsin, ⁴²University of Pennsylvania, ⁴³University of California, Los Angeles, ⁴⁴University of California, Davis, ⁴⁵Boston University, ⁴⁶Indiana University, ⁴⁷Washington University St. Louis, ⁴⁸Eisai, ⁴⁹Harvard University, ⁵⁰University of Washington, ⁵¹Northern California Institute for Research and Education, ⁵²Oregon Health and Science University, ⁵⁴University of California–San Diego, ⁵⁵University of Michigan, ⁵⁷Baylor College of Medicine, ⁵⁸Columbia University Medical Center, ⁶⁰University of Alabama Birmingham, ⁶¹Mount Sinai School of Medicine, ⁶²Rush University Medical Center, ⁶³Wein Center, ⁶⁴Johns Hopkins University, ⁶⁵New York University, ⁶⁶Duke University Medical Center, ⁶⁸University of Kentucky, ⁶⁹University of Pittsburgh, ⁷⁰University of Rochester Medical Center, ⁷¹University of California, Irvine, ⁷²University of Texas Southwestern Medical School, ⁷³Emory University, ⁷⁴University of Kansas, Medical Center, ⁷⁶Mayo Clinic, Jacksonville, ⁷⁸Yale University School of Medicine, ⁷⁹McGill University, Montreal-Jewish General Hospital, ⁸⁰Sunnybrook Health Sciences, Ontario, ⁸¹University of British Columbia Clinic for AD & Related Disorders, ⁸²Cognitive Neurology St. Joseph's Ontario, ⁸³Cleveland Clinic Lou Ruvo Center for Brain Health, ⁸⁴Northwestern University, ⁸⁵Premiere Research Inst (Palm Beach Neurology), ⁸⁶Georgetown University Medical Center, ⁸⁷Brigham and Women's Hospital, ⁸⁸Stanford University, ⁸⁹Banner Sun Health Research Institute, ⁹¹Howard University, ⁹²Case Western Reserve University, ⁹³University of California, Davis Sacramento, ⁹⁴Parkwood Hospital, ⁹⁶Banner Alzheimer's Institute, ⁹⁷Dent Neurologie Institute, ⁹⁸Ohio State University, ⁹⁹Albany Medical College, ¹⁰⁰Hartford Hosp, Olin Neuropsychiatry Research Center, ¹⁰¹Dartmouth-Hitchcock Medical Center, ¹⁰²Wake Forest University Health Sciences, ¹⁰³Rhode Island Hospital, ¹⁰⁴Butler Hospital, ¹⁰⁶University of South Florida, Byrd Institute, ¹⁰⁷University of Chicago, ¹⁰⁸Eastern Virginia Medical School, ¹⁰⁹Charter Health Research Services, ¹¹⁰Houston Methodist Neurological Institute, ¹¹¹Barrow Neurological Institute, ¹¹²Nathan Kline Institute, ¹¹³Ralph Johnson Veterans Administration Health Care Services, ¹¹⁴Vanderbilt University Medical Center, ¹¹⁵University of Texas Health, San Antonio, ¹¹⁶Rutgers University, ¹¹⁷Gonzalez & Aswad Health Services, ¹¹⁸Medical University South Carolina
