# Supplementary material for: Longer scans boost prediction and cut costs in brain-wide association studies
Source: Nature. 2025 Jul 16;644(8077):731–40. doi: 10.1038/s41586-025-09250-1 (PMC12367542; doi:10.1038/s41586-025-09250-1)
Supplement: Supplementary file 2 — Reporting Summary [file 41586_2025_9250_MOESM2_ESM.pdf]

nature portfolio

Corresponding author(s): B. T. Thomas Yeo

Last updated by author(s): May 2, 2025

# Reporting Summary

Nature Portfolio wishes to improve the reproducibility of the work that we publish. This form provides structure for consistency and transparency in reporting. For further information on Nature Portfolio policies, see our Editorial Policies and the Editorial Policy Checklist.

## Statistics

For all statistical analyses, confirm that the following items are present in the figure legend, table legend, main text, or Methods section.

| n/a | Confirmed | |
|---|---|---|
| ☐ | ☒ | The exact sample size ($n$) for each experimental group/condition, given as a discrete number and unit of measurement |
| ☐ | ☒ | A statement on whether measurements were taken from distinct samples or whether the same sample was measured repeatedly |
| ☐ | ☒ | The statistical test(s) used AND whether they are one- or two-sided<br>*Only common tests should be described solely by name; describe more complex techniques in the Methods section.* |
| ☐ | ☒ | A description of all covariates tested |
| ☐ | ☒ | A description of any assumptions or corrections, such as tests of normality and adjustment for multiple comparisons |
| ☐ | ☒ | A full description of the statistical parameters including central tendency (e.g. means) or other basic estimates (e.g. regression coefficient) AND variation (e.g. standard deviation) or associated estimates of uncertainty (e.g. confidence intervals) |
| ☐ | ☒ | For null hypothesis testing, the test statistic (e.g. $F$, $t$, $r$) with confidence intervals, effect sizes, degrees of freedom and $P$ value noted<br>*Give P values as exact values whenever suitable.* |
| ☒ | ☐ | For Bayesian analysis, information on the choice of priors and Markov chain Monte Carlo settings |
| ☒ | ☐ | For hierarchical and complex designs, identification of the appropriate level for tests and full reporting of outcomes |
| ☐ | ☒ | Estimates of effect sizes (e.g. Cohen's $d$, Pearson's $r$), indicating how they were calculated |

*Our web collection on statistics for biologists contains articles on many of the points above.*

## Software and code

Policy information about availability of computer code

| | |
|---|---|
| Data collection | No software was used for data collection. |
| Data analysis | FreeSurfer 5.3.0; FSL 5.0.8; MATLAB (2018b); Python 3.7. Code for this study is publicly available in the GitHub repository maintained by the Computational Brain Imaging Group (https://github.com/ThomasYeoLab/CBIG). Processing pipelines of the fMRI data can be found here (https://github.com/ThomasYeoLab/CBIG/tree/master/stable_projects/preprocessing/CBIG_fMRI_Preproc2016). Code specific to the analyses in this study can be found here (https://github.com/ThomasYeoLab/CBIG/tree/master/stable_projects/predict_phenotypes/Ooi2024_ME). |

For manuscripts utilizing custom algorithms or software that are central to the research but not yet described in published literature, software must be made available to editors and reviewers. We strongly encourage code deposition in a community repository (e.g. GitHub). See the Nature Portfolio guidelines for submitting code & software for further information.

## Data

Policy information about availability of data

All manuscripts must include a data availability statement. This statement should provide the following information, where applicable:

- Accession codes, unique identifiers, or web links for publicly available datasets
- A description of any restrictions on data availability
- For clinical datasets or third party data, please ensure that the statement adheres to our policy

The raw data for HCP (https://www.humanconnectome.org/), ABCD (https://abcdstudy.org/), TCP (https://openneuro.org/datasets/ds005237 and https://

nda.nih.gov/edit_collection.html?id=3552) and ADNI (https://ida.loni.usc.edu/) are publicly available. The ADNI user agreement does not allow us to share the ADNI derivatives. The SINGER dataset can be obtained via a data-transfer agreement (https://medicine.nus.edu.sg/macc/projects/singer/). The MDD dataset is available upon request to co-author HL (hesheng@biopic.pku.edu.cn).

The prediction accuracies for each phenotype, sample size N, and scan time T in all six datasets are publicly available (https://github.com/ThomasYeoLab/CBIG/tree/master/stable_projects/predict_phenotypes/Ooi2024_ME). ABCD parcellated time courses can be found on NDA (dx.doi.org/10.15154/1528763). HCP and TCP parcellated time courses can be found on Zenodo (dx.doi.org/10.5281/zenodo.15300607).

## Research involving human participants, their data, or biological material

Policy information about studies with human participants or human data. See also policy information about sex, gender (identity/presentation), and sexual orientation and race, ethnicity and racism.

**Reporting on sex and gender**

HCP sex distribution: 371 Male / 421 Female
ABCD sex distribution: 1251 Male / 1314 Female
SINGER sex distribution: 309 Male / 333 Female
TCP sex distribution: 81 Male / 110 Female / 3 Self declared
MDD sex distribution: 101 Male / 186 Female
ADNI sex distribution: 278 Male / 308 Female

**Reporting on race, ethnicity, or other socially relevant groupings**

Information related to race and ethnicity was not used in this study.

**Population characteristics**

HCP: 792 young adult (ages 22-35) participants were recruited from families with twins and non-twin siblings.
ABCD: 2565 children (ages 9-10)
SINGER: 642 adults aged 60-80 at risk of cognitive impairment and dementia
TCP: 194 adults (ages 18-70) meeting diagnostic criteria for a broad range of psychiatric illnesses and a healthy comparison group
MDD: 287 participants who meet the diagnostic criteria of DSM-5(Diagnostic and Statistical Manual of Mental Disorders, Fifth Edition) for depression disorder without psychotic symptoms, and currently experiencing a recurrence episode.
ADNI: 586 participants aged 55-90 years, consisting of cognitively normal individuals, those with mild cognitive impairment (MCI), and Alzheimer's disease patients, undergoing extensive neuroimaging and cognitive assessments.

**Recruitment**

Recruitment was carried out by the respective studies.

**Ethics oversight**

The HCP data collection was approved by a consortium of institutional review boards (IRBs) in the United States and Europe, led by Washington University in St Louis and the University of Minnesota (WU-Minn HCP Consortium).

Most ABCD research sites relied on a central IRB at the University of California, San Diego for the ethical review and approval of the research protocol, with a few sites obtaining local IRB approval.

The SINGER study has been approved by the National Healthcare Group Domain-Specific Review Board and is registered under ClinicalTrials.gov (ID: NCT05007353) with written informed consent obtained from all participants before enrolment into the study.

Participants from the TCP study were provided written informed consent following guidelines established by the Yale University and McLean Hospital (Partners Healthcare) IRBs.

The MDD dataset was collected from multiple rTMS clinical trials, and all data were obtained at the pretreatment stage. These trials include ChiCTR2300067671 (approved by the Institutional Review Boards of Beijing Anding Hospital, Henan Provincial People's Hospital, and Tianjin Medical University General Hospital); NCT05842278, NCT05842291, and NCT06166082 (all approved by the IRB of Beijing HuiLongGuan Hospital); and NCT06095778 (approved by the IRB of the Affiliated Brain Hospital of Guangzhou Medical University).

The ADNI study was approved by the IRBs of all participating institutions with informed written consent from all participants at each site.

Note that full information on the approval of the study protocol must also be provided in the manuscript.

# Field-specific reporting

Please select the one below that is the best fit for your research. If you are not sure, read the appropriate sections before making your selection.

☐ Life sciences ☒ Behavioural & social sciences ☐ Ecological, evolutionary & environmental sciences

For a reference copy of the document with all sections, see nature.com/documents/nr-reporting-summary-flat.pdf

# Behavioural & social sciences study design

All studies must disclose on these points even when the disclosure is negative.

| | |
|---|---|
| Study description | Quantitative cross-sectional study where we train and test predictive models to predict behavioral outcomes from neuroimaging data |
| Research sample | To evaluate the robustness of our theoretical model, we considered a diverse collection of datasets (HCP, ABCD, SINGER, TCP, MDD and ADNI) that span multiple fMRI sequences (single-echo single-band, single-echo multi-band, multi-echo multi-band), coordinate systems (fsLR, fsaverage, MNI152), racial groups (Western and Asian populations), mental disorders (healthy, neurological and psychiatric) and age groups (children, young adults and elderly). |
| Sampling strategy | For each dataset, we exclude participants who meet the exclusion criteria (See 'Data exclusions' section below). As a result, the current sample reflects the maximum available sample size for this study. |
| Data collection | All neuroimaging data were collected through various MRI scanners available at different scanning sites. Behavioral measures were collected based on procedures that were specific to each behavioral test. |
| Timing | HCP: The Human Connectome Project S1200 release started in 2012 and finished collecting data in 2016.<br>ABCD: The data collection for this public dataset is still on-going (however, our lab is not involved in the collection in any form). We first accessed the data in 2018.<br>SINGER: The data collection for this dataset is still on-going (however, our lab is not involved in the collection in any form). We first accessed the data in 2023.<br>TCP: The data collection for this dataset began in 2018 and ended in 2024.<br>MDD: The data collection for this dataset is still on-going (however, our lab is not involved in the collection in any form). We first accessed the data in 2024.<br>ADNI: The data collection for this dataset is still on-going (however, our lab is not involved in the collection in any form). We first accessed the data in 2023. |
| Data exclusions | HCP: We excluded 161 participants who did not have at least 40 minutes of uncensored data or did not have the full set of the 59 non-brain-imaging phenotypes from an initial sample of 953 participants.<br>ABCD: We excluded 2695 participants who did not have at least 15 minutes of uncensored data or did not have the full set of the 37 non-brain-imaging phenotypes from an initial sample of 5260 participants.<br>SINGER: We excluded 117 participants who did not have at least 10 minutes of uncensored data or did not have the full set of the 19 non-brain-imaging phenotypes from an initial sample of 759 participants.<br>TCP: We excluded 47 participants who did not have at least 26 minutes of uncensored data or did not have the full set of the 19 non-brain-imaging phenotypes from an initial sample of 241 participants.<br>MDD: We excluded 19 participants who did not have at least 23 minutes of uncensored data or did not have the full set of the 20 non-brain-imaging phenotypes from an initial sample of 306 participants.<br>ADNI: We excluded 182 participants who did not have at least 9 minutes of uncensored data or did not have the full set of the 6 non-brain-imaging phenotypes from an initial sample of 768 participants.<br><br>More details can be found in the Methods section of the paper (Datasets, phenotypes & participants). |
| Non-participation | We are not aware of how many participants may have dropped out as we are not involved in the data collection process. No data was collected for the current study. |
| Randomization | We did not allocate participants into different experimental groups because they all underwent the same experimental conditions. |

# Reporting for specific materials, systems and methods

We require information from authors about some types of materials, experimental systems and methods used in many studies. Here, indicate whether each material, system or method listed is relevant to your study. If you are not sure if a list item applies to your research, read the appropriate section before selecting a response.

## Materials & experimental systems

| n/a | Involved in the study | |
|---|---|---|
| ☒ | ☐ | Antibodies |
| ☒ | ☐ | Eukaryotic cell lines |
| ☒ | ☐ | Palaeontology and archaeology |
| ☒ | ☐ | Animals and other organisms |
| ☒ | ☐ | Clinical data |
| ☒ | ☐ | Dual use research of concern |
| ☒ | ☐ | Plants |

## Methods

| n/a | Involved in the study | |
|---|---|---|
| ☒ | ☐ | ChIP-seq |
| ☒ | ☐ | Flow cytometry |
| ☐ | ☒ | MRI-based neuroimaging |

# Plants

| | |
|---|---|
| Seed stocks | n/a |
| Novel plant genotypes | n/a |
| Authentication | n/a |

# Magnetic resonance imaging

## Experimental design

| | |
|---|---|
| Design type | Resting-state functional imaging |
| Design specifications | HCP: Each participant underwent 4 runs of resting-state functional imaging with a duration of 14.4 minutes.<br>ABCD: Each participant underwent 4 runs of resting-state functional imaging with a duration of 5 minutes.<br>SINGER: Each participant underwent 1 run of resting-state functional imaging with a duration of 10 minutes.<br>TCP: Each participant underwent 4 runs of resting-state functional imaging with a duration of 6.5 minutes.<br>MDD: Each participant underwent 4 runs of resting-state functional imaging with a duration of 6 minutes.<br>ADNI: Each participant underwent 1 run of resting-state functional imaging with a duration of 9 minutes. |
| Behavioral performance measures | No behavioral performance measures are collected during resting-state functional imaging. |

## Acquisition

| | |
|---|---|
| Imaging type(s) | Functional |
| Field strength | 3T |
| Sequence & imaging parameters | HCP: All participants were scanned on a customized Siemens 3T Skyra using a multi-band sequence. Each fMRI run was acquired with a repetition time (TR) of 0.72s at 2mm isotropic resolution and lasted for 14.4 min.<br>ABCD: Multiple scanners of different makes were used, so the sequences were variable, but each fMRI scan was in 2.4 mm isotropic resolution with a TR of 800 ms.<br>SINGER: The following scanning parameters were used: TR=1000, TE=12/29.75/47.5, voxel size=3×3×3mm.<br>TCP: The following scanning parameters were used: TR = 800 milliseconds, TE = 37 milliseconds, flip angle = 52°, and voxel size =2mm. A multi-band acceleration factor of 8 was applied. An auto-align pulse sequence protocol was used to align the acquisition slices of the functional scans parallel to the anterior commissure-posterior commissure (AC-PC) plane of the MPRAGE and centered on the brain.<br>MDD: Rs-fMRI was acquired with an echo planar imaging (EPI) pulse sequence (TR=3000ms, TE=30ms, flip angle=90°, FOV=240×240, 80×80 matrix, 50 slices, voxel size=3×3×3mm).<br>ADNI: Multiple scanners of different makes were used, so the sequences were variable |
| Area of acquisition | Whole-brain coverage |

Diffusion MRI ☐ Used ☒ Not used

## Preprocessing

| | |
|---|---|
| Preprocessing software | FreeSurfer 5.3.0; FSL 5.0.8 |
| Normalization | Non-linear volumetric and surface projection |
| Normalization template | HCP: fsLR surface<br>ABCD/SINGER/MDD/ADNI: FreeSurfer fsaverage6 surface<br>TCP: MNI152 |
| Noise and artifact removal | HCP: Denoising was done by ICA-FIX, we additionally regressed out the global signal<br>ABCD: We regressed out the global signal, six motion correction parameters, averaged ventricular signal, averaged white matter signal, and their temporal derivatives (18 regressors in total)<br>SINGER:We regressed out the global signal, six motion correction parameters, averaged ventricular signal, averaged white matter signal, and their temporal derivatives (18 regressors in total)<br>TCP: Denoising was done by ICA-FIX, we additionally regressed out the global signal<br>MDD: We regressed out the global signal, six motion correction parameters, averaged ventricular signal, averaged white matter signal, and their temporal derivatives (18 regressors in total) |

| | |
|---|---|
| | ADNI:We regressed out the global signal, six motion correction parameters, averaged ventricular signal, averaged white matter signal, and their temporal derivatives (18 regressors in total) |
| Volume censoring | HCP: Motion outlier frames (FD > 0.2mm, DVARS > 75), along with one volume before and two volumes after, were marked as outliers and subsequently censored.<br>ABCD: Motion outlier frames ( FD > 0.3 mm, DVARS > 50), along with one volume before and two volumes after, were marked as outliers and subsequently censored.<br>SINGER:Motion outlier frames ( FD > 0.3 mm, DVARS > 60), along with one volume before and two volumes after, were marked as outliers and subsequently censored.<br>TCP: No censoring<br>MDD: No censoring<br>ADNI: No censoring |

## Statistical modeling & inference

| | |
|---|---|
| Model type and settings | Predictive |
| Effect(s) tested | Prediction of behavioral outcomes from functional connectivity derived from resting-state fMRI. |

Specify type of analysis: ☒ Whole brain ☐ ROI-based ☐ Both

| | |
|---|---|
| Statistic type for inference<br>(See Eklund et al. 2016) | Cluster-wise statistics are not applicable for our study as our analyses only utilize functional connectivity measures. |
| Correction | Multiple comparisons were corrected using the Benjamini–Yekutieli false discovery rate (FDR) procedure with $q < 0.05$. |

## Models & analysis

| n/a | Involved in the study |
|---|---|
| ☐ | ☒ Functional and/or effective connectivity |
| ☒ | ☐ Graph analysis |
| ☐ | ☒ Multivariate modeling or predictive analysis |

| | |
|---|---|
| Functional and/or effective connectivity | Pearson's correlation |
| Multivariate modeling and predictive analysis | We used kernel ridge regression and linear ridge regression. Model performance was evaluated with Pearson's correlation and coefficient of determination. |
